# Synthesis and biological evaluation of novel *N*-α-haloacylated homoserine lactones as quorum sensing modulators

**DOI:** 10.3762/bjoc.10.265

**Published:** 2014-10-30

**Authors:** Michail Syrpas, Ewout Ruysbergh, Christian V Stevens, Norbert De Kimpe, Sven Mangelinckx

**Affiliations:** 1 Department of Sustainable Organic Chemistry and Technology, Ghent University, Coupure Links 653, B-9000 Ghent, Belgium

**Keywords:** halogenated fatty acids, halogenation, *N*-acylated homoserine lactones, *N*-α-haloacylated homoserine lactones, quorum sensing

## Abstract

Novel *N*-α-haloacylated homoserine lactones, in which a halogen atom was introduced at the α-position of the carbonyl function of the *N*-acyl chain, have been studied as quorum sensing (QS) modulators and compared with a library of natural *N*-acylated homoserine lactones (AHLs). The series of novel analogues consists of α-chloro, α-bromo and α-iodo AHL analogues. Furthermore, the biological QS activity of the synthetic AHL analogues compared to the natural AHLs was evaluated. Halogenated analogues demonstrated a reduced activity in the *Escherichia coli* JB523 bioassay, with the α-iodo lactones being the less active ones and the α-chloro AHLs the most potent QS agonists. Most of the α-haloacylated analogues did not exhibit a significant reduction when tested in the QS inhibition test. Therefore, these novel analogues could be utilized as chemical probes for QS structure–activity studies.

## Introduction

Quorum sensing (QS) is the communication system used by bacteria allowing them to adapt to their environment [1]. Bacteria relying on QS secrete signal molecules, called autoinducers, in order to control the expression of specific target genes important for phenotype expression (e.g., biofilm formation, bioluminescence, virulence expression, etc.) in a population density dependent manner [2]. The importance of QS for virulence development in pathogenic bacteria nowadays is obvious [3]. Therefore, quorum sensing modulation is seen as a new anti-infective strategy [4]. Although the described QS systems can regulate virulence, it is believed that they do not affect the viability of bacteria and it could be expected that disruption of QS will not likely create a selective pressure towards resistant organisms [5]. Despite recent reports supporting this theory, it has to be noted that this view point has been challenged and is still under debate among scientists [6–7]. Most Gram-negative bacteria use *N*-acylated homoserine lactones (AHLs) as signal molecules [8]. The general structure of these compounds consist of a lactone ring with an acyl side chain [9]. The length of the acyl chain is usually between 4–18 carbons and the functionality at the β-carbon position can vary. The QS mechanism in these bacteria relies generally upon two components: a LuxI like protein which is a synthase that produces the autoinducer and a transcriptional activator, i.e, a LuxR like protein which binds to the autoinducer [10]. Different bacterial strains were shown to respond to and produce different but specific autoinducers [11]. Furthermore, recent studies reveal that AHLs facilitate both intra-species communication and inter-kingdom interactions [12].

**Figure 1:**
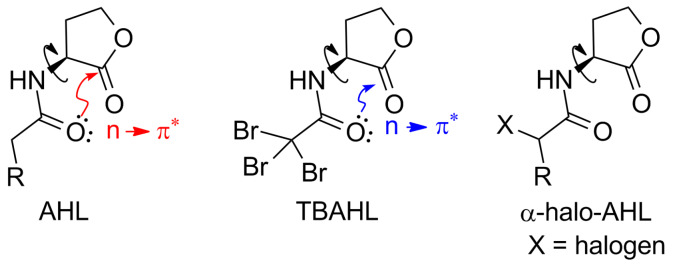
Structure of *N*-acyl homoserine lactone (AHL) and putative n→π* interaction. Attenuation of n→π* interaction in *N*-tribromoacetyl homoserine lactone (TBAHL) as suggested by Newberry and Raines [18]. General structure of compounds used in this study (α-halo-AHL).

The strong impact of AHLs on bacterial behaviour has gained the attention of scientists towards designing AHL mimics that can modulate QS [13–14]. However, the high specificity and affinity that most of the receptors show towards their natural ligands does not allow the design of analogues with high structural deviation from the parent autoinducer [15]. In biological processes, covalent binding and non-covalent interactions are encountered in many protein-ligand interactions as well as in protein secondary structures and are of great significance. In the past years, it was shown that the proximity of carbonyl groups can influence the conformation of molecules by an n→π* interaction [16–17]. Newberry and Raines, very recently proposed that tailored small molecules, that could attenuate this n→π* interaction, in which the lone pair (n) of the *N*-acyl oxygen overlays with the π* orbital of the lactone carbonyl group, could increase the affinity of AHLs to their cognate receptors [18]. It was shown that introduction of halogens as electron-withdrawing groups in the *N*-acyl chain reduced the nucleophilicity of the donor oxygen and led to attenuation of the n→π* interaction (Figure 1). Moreover, inhibition of bacterial QS was recently demonstrated by a set of electrophilic probes (which included halogenated analogues of AHLs) that could covalently bind in the LasR binding pocket of *Pseudomonas aeruginosa* [15]. Therefore, designing analogues with small reactive moieties, such as halogenated carbon atoms, that could be involved in such interactions seem an interesting strategy to be explored.

Surprisingly, most of the studies focus on altering the fatty acid tail or the lactone ring of the AHL molecules [14,19], while modifications at the α-carbon position with respect to the carbonyl function of the *N*-acyl chain are scarce up to date. It was envisioned that modification of an AHL molecule by introduction of a halogen atom in α-position of the carbonyl group of the *N*-acyl chain could possibly generate a novel class of QS modulators. Halogenated fatty acids have been reported as minor components of microorganisms, algae, marine invertebrates, and some plants and animals [20–21]. Interest in these naturally occurring products is high as they often exhibit fascinating biological activities [22–24]. Furthermore, due to the reactivity of halogen substituents, this novel class of compounds can be regarded as building blocks for further elaborations towards new analogues of AHLs. Therefore this study aimed towards the synthesis and biological evaluation of a series of novel halogenated analogues, in which a chlorine, bromine or iodine atom was introduced. To further evaluate the influence of a halogen introduction, the bioactivity of these novel *N*-α-haloacylated homoserine lactones was directly compared with a broad library of naturally occurring AHLs.

## Results and Discussion

### Synthesis of *N*-acylated homoserine lactones and halogenated analogues

**Scheme 1:**
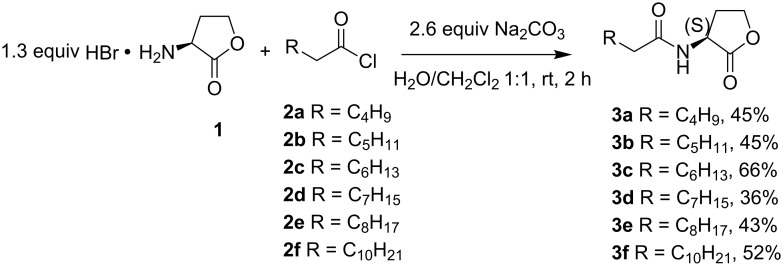
Synthesis of natural AHLs **3a**–**f**.

(*S*)-Homoserine lactone hydrobromide (**1**) was prepared as previously described by reaction of (*S*)-methionine with bromoacetic acid [25]. Reaction of homoserine lactone hydrobromide (**1**) with the corresponding acid chloride **2** under Schotten–Baumann conditions gave the desired *N*-acylated homoserine lactones **3a**–**f** (Scheme 1) [26]. These reaction conditions were used for the preparation of AHLs with unfunctionalized acyl chains in a convenient way. The brominated fatty acids **5**, except the commercially available α-bromohexanoic acid (**5a**), were prepared by a Hell–Volhard–Zelinsky bromination of the corresponding fatty acid **4** with molecular bromine and thionyl chloride [27]. These reaction conditions allowed the synthesis of α-brominated fatty acids **5** in a highly selective and efficient manner.

**Scheme 2:**
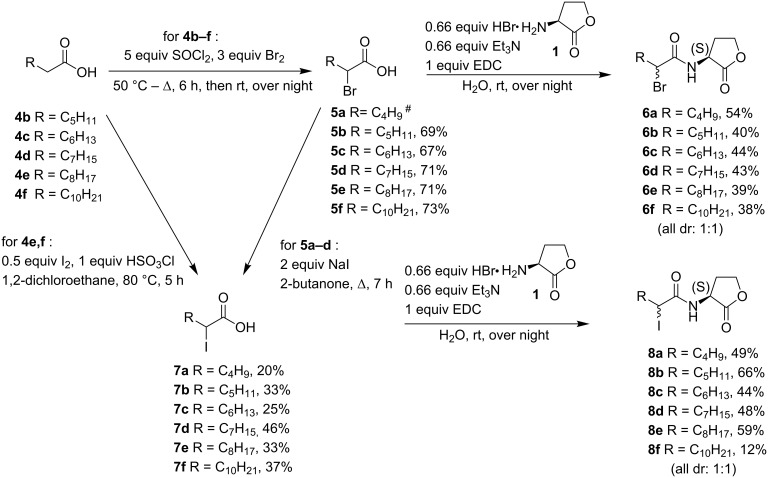
Synthesis of brominated AHLs **6a**-**f** and iodinated AHLs **8a**–**f** (^#^commercially available compound).

Brominated AHL analogues **6a–f** (dr 1:1) were prepared in acceptable yields by 1-ethyl-3-(3-dimethylaminopropyl)carbodiimide (EDC)-mediated coupling of the appropriate α-bromo fatty acid **5** with (*S*)-homoserine lactone **1** (Scheme 2).

For α-iodo fatty acids **7**, two different routes were evaluated. Iodinated fatty acids **7e**,**f** were prepared via reaction of the corresponding fatty acids **4e**,**f** with iodine and chlorosulfonic acid [28]. However, this transformation was not quantitative as it resulted in a mixture of both iodinated and non-iodinated fatty acids. Therefore, α-iodo fatty acids **7a**–**d** were prepared via direct substitution of the corresponding α-bromo fatty acids **5a**–**d** with sodium iodide in 2-butanone [27]. The iodinated AHL analogues **8a**–**f** (dr 1:1) were subsequently prepared under the same coupling conditions as for the brominated analogues **6** (Scheme 2). For compounds **8e**,**f**, the above mentioned mixtures of α-iodo fatty acids **7e**,**f** and fatty acids **4e**,**f** were used and the desired α-iodinated lactones **8e**,**f** could successfully be separated from the reaction mixture via column chromatography.

**Scheme 3:**
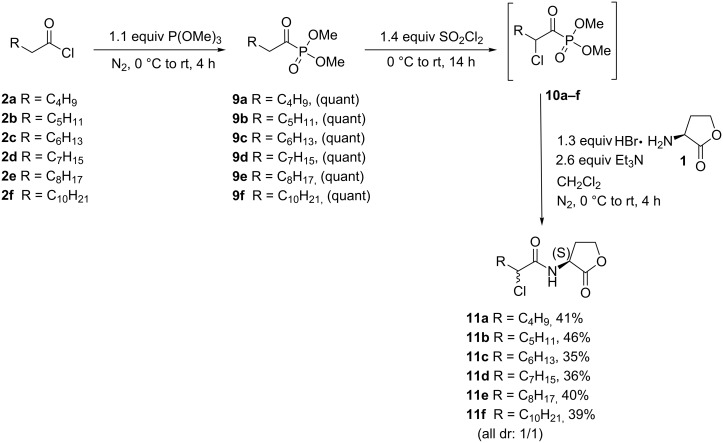
Synthesis of chlorinated AHL analogues **11a**–**f**.

Chlorinated AHL analogues **11a**–**f** were prepared via a one pot procedure (Scheme 3). In this route the acylphosphonates **9a**–**f** were prepared by an Arbuzov reaction and were subsequently chlorinated via reaction with sulfuryl chloride [29–30].

The phosphonate function is used as a strong activating group for the enolization of the carbonyl function facilitating α-monochlorination under mild conditions. α-Chlorinated acylphosphonates can be easily cleaved by a nucleophile with the expulsion of phosphite. Therefore, the α-chloro acylphosphonates **10a**–**f** were transformed to the corresponding chlorinated AHL analogues **11a**–**f** via reaction with *S*-homoserine lactone hydrobromide (**1**) and triethylamine (Scheme 3).

### Biological evaluation

The above mentioned natural AHLs **3** and their halogenated analogues **6**, **8** and **11** were first tested for their ability to induce fluorescence in the *Escherichia coli* JB523 biosensor (Table 1). *Escherichia coli* JB523 is a highly sensitive reporter strain that contains plasmid pJBA130 derived from the LuxR-P_luxI_ quorum sensing operon of *Vibrio fischeri* expressing the production of stable green fluorescent protein (GFP) in response to exogenous AHLs [31]. In addition, the ability of this biosensor to respond to a broad range of chain lengths and functionalization makes it a good choice for the study of structure–activity relationships of synthesized analogues.

**Table 1:** Quorum sensing-regulated GFP production by *Escherichia coli* JB523 induced by natural AHLs **3** and *α*-haloacylated analogues **6**, **8** and **11**^a^.

Compound			Concentration					

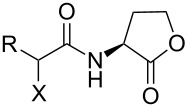	R	X	1000 nM	750 nM	500 nM	250 nM	100 nM	10 nM

**3a**	C_4_H_9_	H	98 ± 4.3	72.9 ± 5.3	58.2 ± 3.3	40 ± 3.6	34.8 ± 2.3	5.7 ± 1.4
**11a**	C_4_H_9_	Cl	48.1 ± 4.0	46.9 ± 6.7	42 ± 3.9	37.4 ± 1.5	25.7 ± 2.9	1.2 ± 0.9
**6a**	C_4_H_9_	Br	19.6 ± 2.1	21 ± 2.5	14.8 ± 3.4	7.3 ± 4.4	2.6 ± 1.3	0 ± 1.1
**8a**	C_4_H_9_	I	26.8 ± 6.0	16.9 ± 5.2	12.5 ± 6.9	7.5 ± 7.8	1.2 ± 2.1	0.1 ± 1.4
**3b**	C_5_H_11_	H	74.6 ± 6.1	91.6 ± 2.1	74.5 ± 3.8	81.1 ± 7.4	70.6 ± 6.0	29.1 ± 11.2
**11b**	C_5_H_11_	Cl	63 ± 3.3	69 ± 10.0	56.9 ± 3.1	54.2 ± 7.9	27.5 ± 5.2	3.2 ± 10.0
**6b**	C_5_H_11_	Br	24.2 ± 0.5	20 ± 2.6	14.4 ± 2.7	10.5 ± 3.7	4.4 ± 3.9	0.2 ± 2.9
**8b**	C_5_H_11_	I	4 ± 8.9	2.2 ± 3.3	1.6 ± 3.1	0.8 ± 1.9	0.3 ± 2.5	0.1 ± 1.3
**3c**	C_6_H_13_	H	57 ± 9.3	59.8 ± 6.3	45.4 ± 1.5	50.8 ± 7.2	39.1 ± 3.2	12.1 ± 2.9
**11c**	C_6_H_13_	Cl	31.4 ± 3.0	28.4 ± 5.3	24 ± 2.6	16.8 ± 3.6	7.7 ± 3.7	0.5 ± 1.4
**6c**	C_6_H_13_	Br	30.8 ± 42.4	21.8 ± 20.7	12.1 ± 33.1	13.4 ± 8.4	2.5 ± 29.2	1.4 ± 5.7
**8c**	C_6_H_13_	I	21.7 ± 37.3	30.9 ± 2.8	8.5 ± 30.0	7.1 ± 11.3	1 ± 18.0	0.6 ± 5.3
**3d**	C_7_H_15_	H	45 ± 5.7	51.5 ± 5.6	42.5 ± 8.3	41.2 ± 4.8	24.2 ± 3.8	1.1 ± 1.2
**11d**	C_7_H_15_	Cl	12.4 ± 5.9	9.8 ± 2.1	5.8 ± 3.1	2.9 ± 2.4	1.2 ± 2.9	0 ± 4.8
**6d**	C_7_H_15_	Br	2.4 ± 2.2	2.2 ± 20.2	0.6 ± 3.1	0.5 ± 15.1	0 ± 2.9	0 ± 6.7
**8d**	C_7_H_15_	I	0.2 ± 1.6	0.2 ± 15.3	0 ± 2.0	0 ± 10.2	0 ± 12.9	0 ± 9.3
**3e**	C_8_H_17_	H	61.7 ± 4.9	42 ± 4.4	27 ± 4.2	8.5 ± 3.0	1.9 ± 3.7	0 ± 3.6
**11e**	C_8_H_17_	Cl	50 ± 6.5	54.2 ± 5.0	47.9 ± 3.1	30.7 ± 2.4	10.9 ± 3.8	0.5 ± 2.9
**6e**	C_8_H_17_	Br	4.4 ± 13.9	2.2 ± 4.7	1.3 ± 3.1	0 ± 1.9	0 ± 2.1	0 ± 2.4
**8e**	C_8_H_17_	I	0 ± 8.9	0 ± 6.5	0 ± 12.1	0 ± 1.9	0 ± 3.5	0 ± 2.0
**3f**	C_10_H_21_	H	18.1 ± 2.7	7 ± 5.3	2.8 ± 3.4	1.4 ± 7.3	0.4 ± 2.9	0 ± 5.9
**11f**	C_10_H_21_	Cl	0.8 ± 2.0	0.5 ± 4.8	1.3 ± 36.9	0.3 ± 2.8	0.1 ± 1.6	0 ± 2.4
**6f**	C_10_H_21_	Br	1.3 ± 4.6	0.3 ± 2.5	0.4 ± 2.1	0.1 ± 1.5	3.6 ± 2.8	0 ± 1.9
**8f**	C_10_H_21_	I	2.9 ± 4.9	1.4 ± 2.5	0.8 ± 1.5	0.4 ± 2.8	0.3 ± 2.9	0 ± 2.9

^a^GFP production was determined by measuring specific fluorescence. GFP fluorescence was corrected for cell density of the reporter strain (fluorescence/OD600 nm). Phosphate buffered saline was used as control; the specific fluorescence observed for 50 nM of OHHL was set at 100% and the other values were normalized accordingly. Results are expressed as mean value ± standard deviation of six repetitions.

The ability of the natural AHLs **3** and their halogenated analogues **6**, **8** and **11** to induce fluorescence was expressed as percentage of relative fluorescence. The activity of these compounds was compared with fluorescence induced by 50 nM of *N*-3-oxohexanoyl homoserine lactone (OHHL), the most active autoinducer in the *Escherichia coli* bioassay. Most of the natural AHLs **3a**–**f** showed agonistic activity in the whole range of tested concentrations (1000–10 nM) (Table 1). In correspondence with previous studies this indicates that the 3-oxo moiety of the fatty acid chain is not an absolute requirement for activity in this biosensor [32].

**Figure 2:**
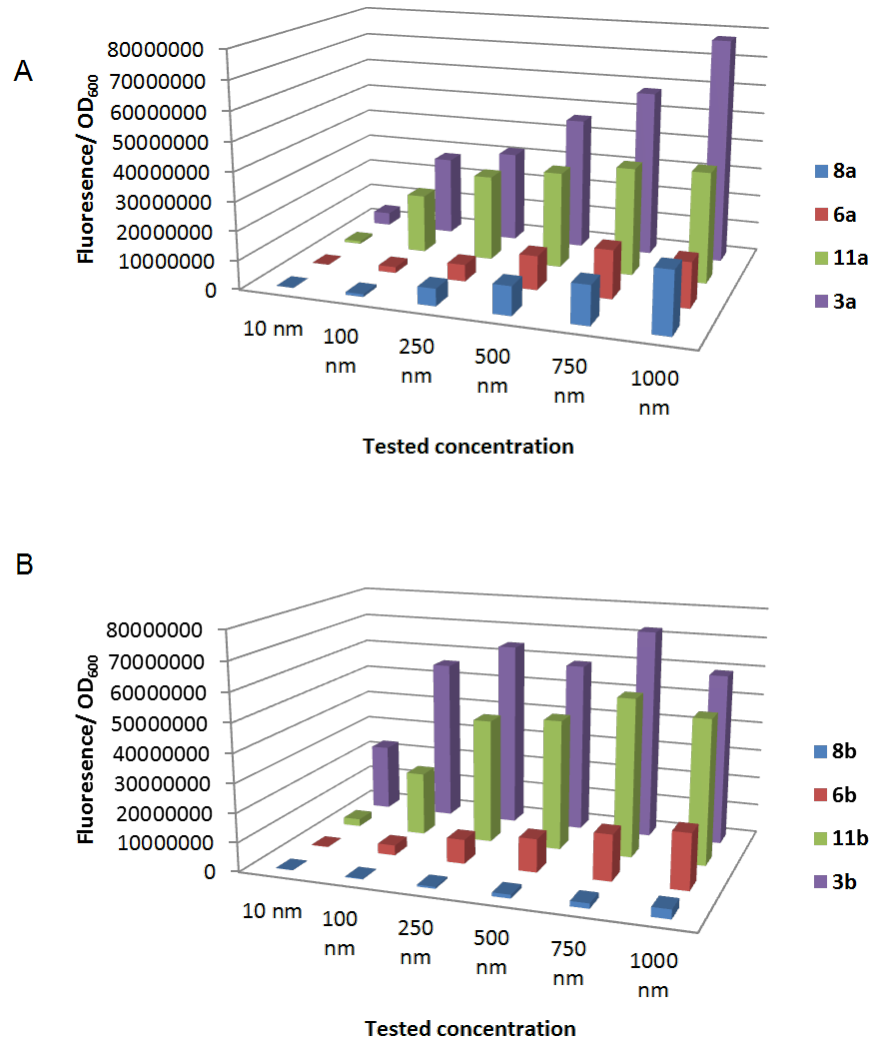
Normalized fluorescence values for natural AHLs **3a**,**b** and α-haloacylated analogues **6**, **8** and **11** tested in various concentrations. A; comparison of AHLs with chain length of 6 carbons. B; comparison of AHLs with chain length of 7 carbons. See Supporting Information File 1 for full experimental data.

As expected, the activity of the natural AHLs **3** decreased with increasing acyl chain length with *N*-hexanoyl and *N*-heptanoyl homoserine lactone **3a** and **3b** showing the highest activity. Introduction of a chlorine atom at the α-position of the acyl chain lead to a decrease of GFP production as can be seen for analogues **11a**,**b** (Figure 2).

In general chlorinated lactones **11a**–**f** were the most active among the α-haloacylated analogues. Nevertheless, this modification reduced the activity as compared to the natural AHLs **3a**–**f** with the same *N*-acyl chain length except for **11e**. Brominated analogues **6a**–**f** showed a further reduced activity when compared to their corresponding natural and chlorinated analogues. α-Bromo lactones **6a**–**b** showed a significantly lower activity (less than half of the activity of their corresponding natural AHLs **3a**,**b**) whereas **6d**–**f** showed practically no activity in the whole concentration range (1000–10 nM). Iodinated lactones **8a**–**f** demonstrated the lowest activity, with very low or no activity in the concentration range of 500 nM to 10 nM. Even at the highest concentration tested (1000 nM), introduction of iodine had a tremendous effect and reduced the activity with more than 75% as can be seen for analogues **8a**,**b** (Figure 2). Overall, these results demonstrate that the agonistic activity of the different halogenated analogues (Cl > Br > I) correlated with the electronegativity and possible n→π^*^ attenuation of the α-halogen (Cl > Br > I). The α-halogenated analogues **6**, **8** and **11** were then screened in an inhibition test competing with OHHL in the *Escherichia coli* JB523 bioassay to evaluate their ability to disrupt QS (Table 2). None of the α-chloro lactones **11** showed a significant reduction of QS regulated GFP-production in the tested concentration range (50–0.05 μΜ). Brominated analogues **6** demonstrated a small decrease in the activity. Among these analogues, compounds **6d** and **6a** inhibited QS the most in the concentration of 50 μΜ and 0.5 μΜ, respectively. α-Iodo lactones **8c**,**d** exhibited a 25% decrease in the 50 μΜ concentration.

**Table 2:** Inhibition of QS regulated GFP production in the *Escherichia coli* JB 523 bioassay by *α*-haloacylated analogues **6**, **8** and **11**^a^.

Compound			Concentration						

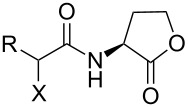	R	X	50 µM	25 µM	12.5 µM	5 µM	0.5 µM	0.05 µM	0 µM

**11a**	C_4_H_9_	Cl	77.9 ± 51.5	108 ± 2.3	97.6 ± 2.5	95.1 ± 1.8	91.1 ± 1.7	95.4 ± 2.5	100 ± 2.0
**6a**	C_4_H_9_	Br	97.9 ± 4.7	101 ± 5.0	90.9 ± 6.9	86.2 ± 7.4	71.6 ± 3.8	84.1 ± 2.0	100 ± 3.5
**8a**	C_4_H_9_	I	101 ± 7.1	97.5 ± 2.5	95.4 ± 3.7	91.8 ± 2.0	98.2 ± 1.5	97.7 ± 2.1	100 ± 2.4
**11b**	C_5_H_11_	Cl	105 ± 1.2	104 ± 2.3	105 ± 1.9	103 ± 1.9	99.8 ± 2.3	94.6 ± 2	100 ± 2.8
**6b**	C_5_H_11_	Br	82.5 ± 6.0	79 ± 2.0	78.9 ± 2.1	82.5 ± 2.9	88.1 ± 4.1	93.8 ± 4.0	100 ± 3.1
**8b**	C_5_H_11_	I	73.4 ± 2.9	69.4 ± 15.3	77.7 ± 3.4	83.9 ± 2.6	94.3 ± 3.5	97 ± 2.7	100 ± 2.6
**11c**	C_6_H_13_	Cl	98.6 ± 3.0	95.5 ± 2.3	91.4 ± 5.9	85.9 ± 2.6	93.2 ± 3.0	96.4 ± 2.9	100 ± 4.4
**6c**	C_6_H_13_	Br	85.2 ± 2.5	77.9 ± 3.0	79.8 ± 4.4	74.9 ± 1.4	91 ± 2.6	97 ± 3.7	100 ± 13.1
**8c**	C_6_H_13_	I	75.5 ± 1.7	79.3 ± 1.8	85.1 ± 4.2	88.8 ± 1.6	98.2 ± 2.0	94.1 ± 2.4	100 ± 2.7
**11d**	C_7_H_15_	Cl	84.7 ± 2.7	81.5 ± 1.5	81.7 ± 2.3	84.6 ± 3.0	94.2 ± 1.6	96.4 ± 0.9	100 ± 2.0
**6d**	C_7_H_15_	Br	68.2 ± 2.0	73.6 ± 3.5	77.7 ± 10.8	88.3 ± 2.0	95.6 ± 3.3	95.3 ± 1.5	100 ± 2.1
**8d**	C_7_H_15_	I	75.3 ± 1.5	83.1 ± 5.2	94.2 ± 3.1	93.5 ± 1.3	101 ± 2.2	98.2 ± 3.7	100 ± 2.4
**11e**	C_8_H_17_	Cl	84.3 ± 33.8	118 ± 7.4	93.2 ± 3.5	106 ± 5.0	93.9 ± 7.8	105 ± 4.0	100 ± 1.7
**6e**	C_8_H_17_	Br	86.9 ± 4.6	94.8 ± 2.3	90.9 ± 8.8	104 ± 2.3	105 ± 3.2	113 ± 9.6	100 ± 3.5
**8e**	C_8_H_17_	I	100 ± 6.6	108 ± 7.8	111 ± 2.5	101 ± 4.5	119 ± 14.6	120 ± 12.6	100 ± 16.1
**11f**	C_10_H_21_	Cl	86 ± 5.6	93.4 ± 4.4	90.6 ± 3.0	90.5 ± 3.1	90.6 ± 2.2	98.5 ± 4.1	100 ± 1.0
**6f**	C_10_H_21_	Br	77.9 ± 5.4	91.4 ± 11.0	81.4 ± 7.5	89.6 ± 3.6	78 ± 5.2	84.9 ± 2.6	100 ± 2.3
**8f**	C_10_H_21_	I	90.7 ± 2.0	90.1 ± 2.5	90.2 ± 1.8	93.8 ± 1.6	98.8 ± 1.7	96.9 ± 0.9	100 ± 2.0

^a^Inhibition of quorum sensing-regulated GFP production in the *Escherichia coli* JB523 bioassay by α-haloacylated analogues **6**, **8** and **11** in the presence of 50 nM of OHHL. GFP production was determined by measuring specific fluorescence. GFP fluorescence was corrected for cell density of the reporter strain (Fluorescence/OD600 nm). Phosphate buffer saline was used as control; the specific fluorescence observed for 50 nM of OHHL was set at 100% and the other values were normalized accordingly. Results are expressed as mean value ± standard deviation of six repetitions.

It could be expected that, due to attenuation of the n→π* interaction and the subsequent effect on the conformation of the molecules as it was recently suggested, introduction of electron-withdrawing groups in α-position of the carbonyl group could lead to higher affinity of these analogues towards the receptor [18]. However, under the tested conditions the halogenated analogues **6**, **8** and **11** did not demonstrate a significantly higher QS activity, although most α-chlorinated analogues **11** retained a significant amount of QS activity. It has been reported that the *trans* conformation, in which the dihedral angle between the carbonyl oxygen and the α-chloro atom is ~180°, is the most stable conformation of 2-chloroacetamide in solution. In this conformation the C–Cl bond dipole is aligned in parallel with the N–H bond dipole, promoting a favorable electrostatic interaction [33].

**Figure 3:**
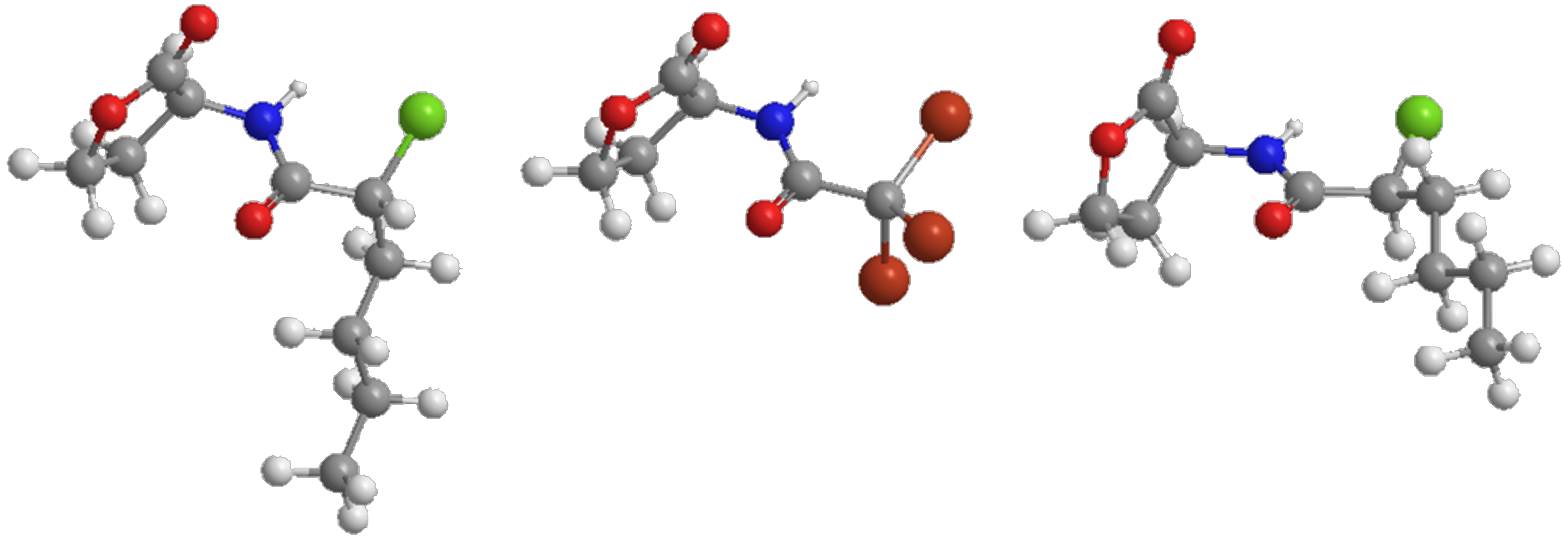
Proposed models (Chem3D Pro) of *N*-(2*R*)- (left) and *N*-(2*S*)-chlorohexanoyl-(*S*)-homoserine lactone (right) **11a** aligned with the optimized structure of *N*-tribromoacetyl homoserine lactone (middle) [18].

The occurrence of these favorable *trans* conformations would result in opposite face orientations of the alkyl chain of the different diastereoisomers of *N*-chloroacyl-(*S*)-homoserine lactones **11**. As can be seen from the proposed models (Chem3D Pro) in which *N*-(2*R*)- and *N*-(2*S*)-chlorohexanoyl-(*S*)-homoserine lactone **11a** (Figure 3) are aligned with the optimized structure of *N*-tribromoacetyl homoserine lactone [18], for the *N*-(2*R*)-chlorohexanoyl-(*S*)-homoserine lactone **11a** the alkyl group is in a position with more steric interactions between the alkyl chain and the lacton ring as compared to the conformation of *N*-(2*S*)-chlorohexanoyl-(*S*)-homoserine lactone **11a**. This possible steric hindrance could contribute to attenuation of the n→π* interaction. The influence of these possible conformational effects, combined with the electron-withdrawing effect of the halogens, will be subject of future theoretical calculations.

Due to the presence of the halogen as an electrophilic moiety, it could be possible that the reduced activity of the novel halogenated analogues **6**, **8** and **11** originates from a covalent binding of the halogenated analogues in the binding site of the target receptor [15]. The absence of antagonistic activity of these molecules in this biosensor however indicates that the reduction in QS activity results from the presence of the larger electron-withdrawing halogen substituents (covalent radius H: 0.37 Å; Cl: 0.99 Å; Br: 1.14 Å; I: 1.33 Å) which causes changes in the steric and electronic properties of the *N*-acyl chain. These results are in agreement with a previous study where it was demonstrated that the final cleavage of the acyl chain and not the halogenation itself leads to the deactivation of β-keto AHLs [34]. In summary, it can be suggested that some of these novel halogenated analogues **6**, **8** and especially **11** could be utilized as tools in QS research, e.g., with wild type bacteria or other reporter strains because of their partially retained activity and the absence of antagonistic effects presently demonstrated.

## Conclusion

In conclusion, new AHL analogues were designed by introduction of a halogen atom in α-position of the *N*-acyl chain and their bioactivity as QS modulators has been evaluated. α-Halogenated AHL analogues exhibited a reduction in activity as compared to the activity of the corresponding natural analogues with the same *N*-acyl chain in the *E. coli* JB523 bioassay. These results do not directly support the recent suggestion that modifications, i.e., introduction of halogens that weakens the n→π* interaction should increase the affinity of AHL analogues with the cognate receptor [18]. However, the activity of the novel compounds is modulated by the steric and electronic effects induced by the larger halo substituents. Furthermore, the absence of antagonistic activity of the novel analogues and the retained QS activity of most of the chlorinated analogues in comparison with the natural ligands encourages the utilization of these functionalized compounds as possible tools for further exploration of QS structure–activity studies.

## Experimental

### Chemistry

#### Synthesis of homoserine lactone hydrobromide **1**

As already described in [34] a mixture of (*S*)-methionine (15.2 g; 0.10 mol) and bromoacetic acid (1.1 equiv, 15.4 g, 0.11 mol) in 150 mL of a H_2_O–iPrOH–AcOH mixture (5:5:2 v:v:v) was stirred under reflux overnight. The solvent was then removed under reduced pressure. Subsequently, the orange sticky oil was partly dissolved in 50 mL of a 4:1 mixture (v:v) of iPrOH–HBr (30% in AcOH). The title compound was collected by filtration and the purification procedure was repeated starting by evaporation of the orange filtrate to dryness. Compound **1** was collected as a beige powder, mp 227–229 °C (mp 226–228 °C [25]). All other physical and analytical data were in agreement with those previously reported.

#### Synthesis of natural AHLs **3** using Schotten–Baumann conditions

The appropriate acid chloride **2** (4 mmol) was added dropwise at room temperature to a vigorously stirred mixture of (*S*)-homoserine lactone hydrobromide (**1**, 1.3 equiv, 0.95 g, 5.2 mmol) and Na_2_CO_3_ (2.6 equiv, 0.55 g, 10.4 mmol) in water (5 mL per mmol acid chloride) and CH_2_Cl_2_ (5 mL per mmol acid chloride). The mixture was stirred vigorously at room temperature for 2 h. CH_2_Cl_2_ was added, the aqueous and organic phases were separated and the aqueous phase was extracted with CH_2_Cl_2_ (2 × 20 mL). The combined organic extracts were washed with saturated aqueous NaHCO_3_ (2 × 40 mL), dried (MgSO_4_), filtered and the solvent removed under reduced pressure to yield the desired product **3**. The crude product was purified via column chromatography on silica gel (EtOAc/petroleum ether 4:1).

#### Synthesis of α-brominated fatty acids **5b–f**

To a flask equipped with a sodium hydroxide trap the corresponding fatty acid **4** (0.065 mol) and thionyl chloride (5 equiv, 40 mL, 0.325 mol) were added. The reaction mixture was stirred at 50 °C in a water bath. Then, liquid bromine (3 equiv, 5 mL, 0.195 mol) was added dropwise and the reaction mixture was stirred under reflux for 6 h and then at room temperature overnight. The excess of thionyl chloride was then removed by distillation. To a beaker containing distilled water in an ice bath, the dark red residue was slowly added and manually stirred. Afterwards, the solution was heated and subsequently cooled, resulting in the precipitation of a pale yellow solid for the longer chain fatty acids which was decanted, washed with distilled water, and once again submitted to the crystallization procedure.

#### Synthesis of α-iodinated fatty acids **7a–d**

The appropriate α-bromo fatty acid **5** (3 mmol) and sodium iodide (2 equiv, 0.90 g, 6 mmol) were dissolved in 10 mL of 2-butanone. The reaction mixture was stirred under reflux for 7 h. Subsequently the solvent was removed under reduced pressure and the brown residue was dissolved in distilled water and subsequently decolorized by dropwise addition of 10% aq NaHSO_3_. The solution was then acidified with 5% aq H_2_SO_4_, yielding a black oil which was separated from the aqueous phase by decantation. Distilled water was then added and the suspension was heated to boiling with the dropwise addition of 10% aq NaHSO_3_ until complete clearing of the oil. The aqueous phase was removed and the yellow oil was dissolved in a saturated aq NaHCO_3_ solution, the medium was acidified with 5% aq H_2_SO_4_ and heated. The dense yellow oil was extracted with ethyl acetate, dried over anhydrous magnesium sulfate, filtered and evaporated under reduced pressure.

#### Synthesis of α-iodinated fatty acids **7e,f**

As described in [28] fatty acid **4e** or **4f** (1.56 mmol), iodine (0.5 equiv, 0.20 g, 0.78 mmol) and chlorosulfonic acid (1 equiv, 0.1 mL, 1.56 mmol) were dissolved in 1.6 mL of dry 1,2-dichloroethane. The reaction mixture was heated at 80 °C for 5 h, after which it was diluted with 3 mL 1,2-dichloroethane and washed successively with water (2 × 5 mL) and an aqueous 0.1 M Na_2_S_2_O_3_ solution until the color changed from pink to white. The organic phase was dried with MgSO_4_, filtered and evaporated in vacuo.

#### Synthesis of α-bromo-acylated homoserine lactones **6a–f** and α-iodo acylated homoserine lactones **8a–f**

As described in [34] to a stirred solution of (*S*)-homoserine lactone hydrobromide (**1**, 0.36 g, 2 mmol) in 5 mL of water, triethylamine (1 equiv, 0.28 mL, 2 mmol) was added followed by the addition of the appropriate brominated or iodinated fatty acid **5** or **7** (1.5 equiv, 3 mmol) and 1-ethyl-3-(3-dimethylaminopropyl)carbodiimide (1.5 equiv, 0.58 g, 3 mmol). The mixture was stirred overnight at room temperature. The aqueous phase was extracted two times with ethyl acetate and the organic phase was washed with saturated aq NaHCO_3_ solution and brine. Drying with MgSO_4_, filtration and evaporation of the solvent gave the corresponding acylated homoserine lactones **6** and **8**. The crude product was purified via column chromatography on silica gel (EtOAc/petroleum ether 4:1).

#### Synthesis of α-chloro acylated homoserine lactones **11a–f**

In a flask containing the appropriate acid chloride **2** (5 mmol) which was kept under a nitrogen atmosphere at 0 °C, P(OMe)_3_ (1.16 equiv, 1.45 mL, 5.8 mmol) was added. The mixture was stirred for 4 h at room temperature. Then the excess of P(OMe)_3_ and MeCl were evaporated under reduced pressure. The flask was covered with aluminium foil and SO_2_Cl_2_ (1.3 equiv, 0.52 mL, 6.5 mmol) was added via a syringe at 0 °C. After stirring for 14 h at room temperature, the reaction was quenched by adding 5 mL of dry CH_2_Cl_2_ and bubbling nitrogen through the solution to remove excess of SO_2_Cl_2_ and HCl. The chlorinated acylphosphonates **10** were added at 0 °C to a bulb containing (*S*)-homoserine lactone **1** (1.3 equiv, 1.18 g, 6.5 mmol) and Et_3_N (2.6 equiv, 1.8 mL, 13 mmol) in 10 mL of dry CH_2_Cl_2_. This reaction mixture was stirred for 4 h at room temperature under nitrogen. Then the mixture was poured into 20 mL 0.1 M aq HCl and extracted with 20 mL of CH_2_Cl_2_. The organic phase was dried with MgSO_4_, filtered to remove the MgSO_4_ and the solvent was removed under reduced pressure. The crude product was purified by column chromatography on silica gel (EtOAc/petroleum ether 4:1) to give the desired product.

### Characterization of novel compounds

Representative characterization data for novel analogues. Characterization of all compounds can be found in the Supporting Information File 2 of this article.

#### 2-Bromo-*N*-[(3*S*)-tetrahydro-2-oxo-3-furanyl]hexanamide) (**6a**)

^1^H NMR (300 MHz, CDCl_3_) δ 0.92 (t, *J* = 7.0 Hz, 3H, C*H*_3isomer 1 and 2_), 1.30–1.53 (m, 4H, (C*H*_2_)_2_CH_3isomer 1 and 2_), 1.94–2.07 (m, 1H, C*H*H'CHBr_isomer 1 and 2_), 2.08–2.16 (m, 1H, CH*H*'CHBr_isomer 1 and 2_), 2.16–2.30 (m, 1H, OCH_2_C*H*H'_isomer 1 and 2)_, 2.79–2.90 (m, 1H, OCH_2_CH*H*'_isomer 1 and 2_), 4.22–4.36 (m, 2H, C*H*Br_isomer 1 and 2_ and OCH*H*'_isomer 1 and 2_), 4.50 (t, *J* = 9.1 Hz, 1H, OC*H*H'_isomer 1 and 2_), 4.52–4.61 (m, 1H, C*H*N_isomer 1 and 2_), 7.01 (d, *J* = 5.5 Hz, 1H, N*H*_,isomer 1 and 2_); ^13^C NMR (75 MHz, CDCl_3_) δ 13.9 (*C*H_3isomer 1 and 2_), 22.0 (*C*H_2_CH_3isomer 1 and 2_), 29.35 (*C*H_2_CH_2_CHBr_isomer 1_), 29.39 (*C*H_2_CH_2_CHBr_isomer 2_), 29.9 (*C*H_2_CHN_isomer 1 and 2_), 35.36 (*C*H_2_CHBr_isomer 1_), 35.41 (*C*H_2_CHBr_isomer 2_), 49.8 (*C*HN_isomer 1 and 2_), 50.3 (*C*HBr_isomer 1_), 50.4 (*C*HBr_isomer 2_), 66.17 (*C*H_2_O_isomer 1_), 66.20 (*C*H_2_O_isomer 2_), 169.8 (N*C*=O_isomer 1_), 169.9 (N*C*=O_isomer 2_), 174.9 (O*C*=O_isomer 1_), 175.0 (O*C*=O_isomer 2_); MS (ESI) *m*/*z* (%): 278/280 (M + H^+^, 100); HRMS calcd for C_10_H_16_BrNO_3_H^+^, 278.0386; found, 278.0378; IR (cm^−1^) ν_max_: 1008, 1178 (C-O), 1554 (HN-C=O), 1655 (HN-C=O), 1769 (C=O_lactone_), 2860 (CH), 2924 (CH), 2957 (CH), 3298 (NH); chromatography: EtOAc/PE 4:1 *R*_f_ 0.48; melting point: 148 °C; white powder; yield: 54%.

#### 2-Iodo-*N*-[(3*S*)-tetrahydro-2-oxo-3-furanyl]hexanamide) (**8a**)

^1^H NMR (300 MHz, CDCl_3_) δ 0.91 (t, *J =* 7.2 Hz, 3H, C*H*_3, isomer 1 and 2_), 1.21–1.50 (m, 4H, (C*H*_2_)_2_CH_3, isomer 1 and 2_), 1.99 (q, *J =*7.2 Hz, 2H, C*H*_2_CHI_isomer 1 and 2_), 2.11–2.29 (m, 1H, OCH_2_C*H*H'_isomer 1 and 2_), 2.82–2.94 (m, 1H, OCH_2_CH*H*'_isomer 1 and 2_), 4.26–4.36 (m, 1H, OCH*H*'_isomer 1 and 2_), 4.30 (t, *J =* 7.2 Hz, 1H, C*H*I_isomer 1 and 2_), 4.46–4.53 (m, 0.5H, C*H*N_isomer 1_), 4.51 (t, *J =* 8.8 Hz, 1H, OC*H*H'_isomer 1 and 2_), 4.60 (ddd, *J =* 11.8 Hz, 8.5 Hz, 6.1 Hz, 0.5H, C*H*N_isomer 2_), 6.43 (d, *J =* 6.1 Hz, 0.5H, N*H*_isomer 1_), 6.52 (d, *J =* 6.1 Hz, 0.5H, N*H*_isomer 2_); ^13^C NMR (75 MHz, CDCl_3_) δ 13.9 (*C*H_3, isomer 1 and 2_), 22.0 and 31.6 ((*C*H_2_)_2_CH_3, isomer 1 and 2_), 24.9 (*C*HI_isomer 1 and 2_), 29.6 (*C*H_2_CHN_isomer 1_), 29.7 (*C*H_2_CHN_isomer 2_), 36.2 (*C*H_2_CHI_isomer 1_), 36.3 (*C*H_2_CHI_isomer 2_), 49.6 (*C*HN_isomer 1_), 49.8 (*C*HN_isomer 2_), 66.3 (*C*H_2_O_isomer 1 and 2_), 171.4 (N*C*=O_isomer 1_ ), 171.5 (N*C*=O_isomer 2_), 175.3 (O*C*=O_isomer 1_), 175.4 (O*C*=O_isomer 2_); MS (ESI) *m*/*z* (%): 326 (M + H^+^, 100); HRMS calcd for C_10_H_16_INO_3_H^+^, 326.0253; found, 326.0244; IR (cm^−1^) ν_max_: 1016, 1176 (C-O), 1546 (HN-C=O), 1642 (HN-C=O), 1770 (C=O_lactone_), 2858 (CH), 2929 (CH), 2956 (CH), 3296 (NH); chromatography: EtOAc/PE 4:1 *R*_f_ 0.62; melting point: 152 °C; yellowish powder; yield: 49%.

#### 2-Chloro-*N*-[(3*S*)-tetrahydro-2-oxo-3-furanyl]hexanamide) (**11a**)

^1^H NMR (300 MHz, CDCl_3_) δ 0.92 (t, *J =* 7.2 Hz, 3H, C*H*_3, isomer 1 and 2_), 1.26–1.55 (m, 4H, (C*H*_2_)_2_CH_3isomer 1 and 2_), 1.87–2.01 (m, 1H, C*H*H'CHCl_isomer 1 and 2_), 2.05–2.18 (m, 1H, CH*H*'CHCl_isomer 1 and 2_), 2.26 (dddd, *J =* 11.8 Hz, 11.8 Hz, 11.7 Hz, 8.8 Hz, 1H, OCH_2_C*H*H'_isomer 1 and 2_), 2.74–2.84 (m, 1H, OCH_2_CH*H*’_isomer 1 and 2_), 4.27–4.40 (m, 2H, C*H*Cl_isomer 1 and 2_ and OCH*H*'_isomer 1 and 2_), 4.50 (t, *J =* 8.8 Hz, 1H, OC*H*H'_isomer 1 and 2_), 4.55–4.64 (m, 1H, C*H*N_isomer 1 and 2_), 7.22 (d, *J =* 6.1 Hz, 0.5H, N*H*_isomer 1_), 7.25 (d, *J =* 6.1 Hz, 0.5H, N*H*_isomer 2_); ^13^C NMR (75 MHz, CDCl_3_) δ 13.9 (*C*H_3, isomer 1 and 2_), 22.0 (*C*H_2_CH_3, isomer 1 and 2_), 27.9 (*C*H_2_CH_2_CHCl_isomer 1_), 28.0 (*C*H_2_CH_2_CHCl_isomer 2_), 29.7 (*C*H_2_CHN_isomer 1 and 2_), 35.2 (*C*H_2_CHCl_isomer 1 and 2_), 49.4 (*C*HN_isomer 1_), 49.5 (*C*HN_isomer 2_), 60.47 (*C*HCl_isomer 1_), 60.50 (*C*HCl_isomer 2_), 66.07 (*C*H_2_O_isomer 1_), 66.12 (*C*H_2_O_isomer 2_), 169.96 (N*C*=O_isomer 1_), 170.00 (N*C*=O_isomer 2_), 174.8 (O*C*=O_isomer 1_), 174.9 (O*C*=O_isomer 2_); MS (ESI) *m*/*z* (%): 234/236 (M + H^+^, 100); HRMS calcd for C_10_H_16_ClNO_3_H^+^, 234.0897; found, 234.0889; IR (cm^−1^) ν_max_: 1014, 1172 (C-O), 1553 (HN-C=O), 1656 (HN-C=O), 1771 (C=O_lactone_), 2860 (CH), 2930 (CH), 2958 (CH), 3298 (NH); chromatography: EtOAc/PE 4:1 *R*_f_ 0.48; melting point: 144 °C; white powder; yield: 42%.

### Biological evaluation

#### *Escherichia coli* JB523 green fluorescent protein (GFP) microplate assay

Strain JB523 was grown overnight at 28 °C in Luria–Bertani (LB) medium supplemented with 20 mg/L tetracycline until optical density (OD) reached approximately 1 at 550 nm. The bacteria were then diluted to an OD_600_ of 0.1. Then 100 μL of the diluted culture was mixed with 100 μL of the appropriate concentration of the tested compound. Phosphate buffered saline (PBS) was used to prepare the stock solutions for all concentrations. PBS was used as a negative control for activation tests. For the inhibition tests the procedure was the same with the exception of the addition of 50 nM of OHHL to the culture medium. The plate was incubated at 28 °C for 24 h. QS-regulated GFP production was then assessed by fluorescence measurements (excitation at 475 nm and emission at 515 nm) using a PerkinElmer VICTOR X multilabel plate reader. The fluorescence was normalised for cell density of the reporter strain.

## Supporting Information

File 1 Graphical representation of QS activity of natural and α-haloacylated analogues.

File 2 ^1^H and ^13^C NMR data and spectra, IR, HRMS, optical rotation and melting point data, chromatographic separation and yields of synthesized compounds.

## References

[1] Bassler B L (1999). Curr Opin Microbiol.

[2] de Kievit T R (2009). Environ Microbiol.

[3] Rutherford S T, Bassler B L (2012). Cold Spring Harbor Perspect Med.

[4] Defoirdt T, Boon N, Bossier P, Verstraete W (2004). Aquaculture.

[5] Bjarnsholt T, Givskov M (2007). Anal Bioanal Chem.

[6] Gerdt J P, Blackwell H E (2014). ACS Chem Biol.

[7] Maeda T, Garcia-Contreras R, Pu M, Sheng L, Garcia L R, Tomás M, Wood T K (2012). ISME J.

[8] Whitehead N A, Barnard A M L, Slater H, Simpson N J L, Salmond G P C (2001). FEMS Microbiol Rev.

[9] Decho A W, Frey R L, Ferry J L (2011). Chem Rev.

[10] Fuqua W C, Winans S C, Greenberg E P (1994). J Bacteriol.

[11] Taga M E, Bassler B L (2003). Proc Natl Acad Sci U S A.

[12] Williams P (2007). Microbiology.

[13] Geske G D, O'Neill J C, Blackwell H E (2008). Chem Soc Rev.

[14] Galloway W R J D, Hodgkinson J T, Bowden S D, Welch M, Spring D R (2011). Chem Rev.

[15] Amara N, Mashiach R, Amar D, Krief P, Spieser S A H, Bottomley M J, Aharoni A, Meijler M M (2009). J Am Chem Soc.

[16] Bartlett G J, Choudhary A, Raines R T, Woolfson D N (2010). Nat Chem Biol.

[17] Choudhary A, Fry C G, Raines R T (2010). ARKIVOC.

[18] Newberry R W, Raines R T (2014). ACS Chem Biol.

[19] Geske G D, O'Neill J C, Miller D M, Mattmann M E, Blackwell H E (2007). J Am Chem Soc.

[20] Dembitsky V M, Srebnik M (2002). Prog Lipid Res.

[21] Mu H, Wesén C, Sundin P (1997). TrAC, Trends Anal Chem.

[22] Parang K, Knaus E E, Wiebe L I, Sardari S, Daneshtalab M, Csizmadia F (1996). Arch Pharm.

[23] Anbukumar D S, Shornick L P, Albert C J, Steward M M, Zoeller R A, Neumann W L, Ford D A (2010). J Lipid Res.

[24] Hernanz D, Fabrias G, Camps F (1997). J Lipid Res.

[25] Persson T, Hansen T H, Rasmussen T B, Skindersø M E, Givskov M, Nielsen J (2005). Org Biomol Chem.

[26] Hodgkinson J T, Galloway W R J D, Casoli M, Keane H, Su X, Salmond G P C, Welch M, Spring D R (2011). Tetrahedron Lett.

[27] Pomini A M, Cruz P L R, Gai C, Araújo W L, Marsaioli A J (2009). J Nat Prod.

[28] Van den Bergen H, Daloze D, Braekman J-C (1999). J Braz Chem Soc.

[29] Stevens C V, Vanderhoydonck B (2001). Tetrahedron.

[30] Stevens C, De Buyck L, De Kimpe N (1998). Tetrahedron Lett.

[31] Andersen J B, Heydorn A, Hentzer M, Eberl L, Geisenberger O, Christensen B B, Molin S, Givskov M (2001). Appl Environ Microbiol.

[32] Olsen J A, Severinsen R, Rasmussen T B, Hentzer M, Givskov M, Nielsen J (2002). Bioorg Med Chem Lett.

[33] Pedersoli S, Tormena C F, Rittner R (2008). J Mol Struct.

[34] Syrpas M, Ruysbergh E, Blommaert L, Vanelslander B, Sabbe K, Vyverman W, De Kimpe N, Mangelinckx S (2014). Mar Drugs.

