# Supplementary material for: Synthesis and biological evaluation of novel N-α-haloacylated homoserine lactones as quorum sensing modulators
Source: Beilstein J Org Chem. 2014 Oct 30;10:2539–49. doi: 10.3762/bjoc.10.265 (PMC4222389; doi:10.3762/bjoc.10.265)
Supplement: File 1 — Graphical representation of QS activity of natural and α-haloacylated analogues. [file Beilstein_J_Org_Chem-10-2539-s001.pdf]

Supporting Information File 1

for

Synthesis and biological evaluation of novel *N*-α-haloacylated homoserine lactones as quorum sensing modulators

Michail Syrpas, Ewout Ruysbergh, Christian V. Stevens, Norbert De Kimpe and Sven Mangelinckx*

Address: [1]Department of Sustainable Organic Chemistry and Technology, Ghent University, Coupure Links 653, B-9000 Ghent, Belgium

Email: Sven Mangelinckx - Sven.Mangelinckx@Ugent.be

* Corresponding author

Graphical representation of QS activity of natural and α-haloacylated analogues

Comparison of natural AHLs **3c–f** with α-haloacylated analogues **6**, **8** and **11.**

A

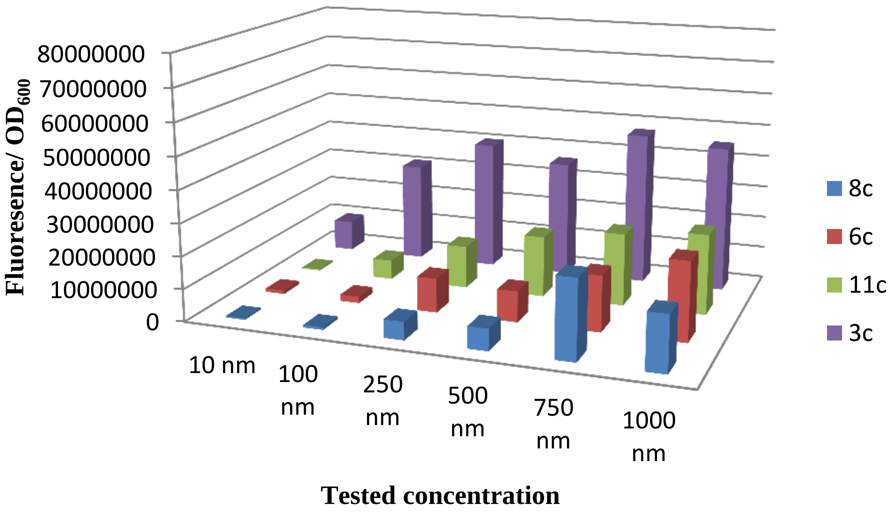

B

C

D

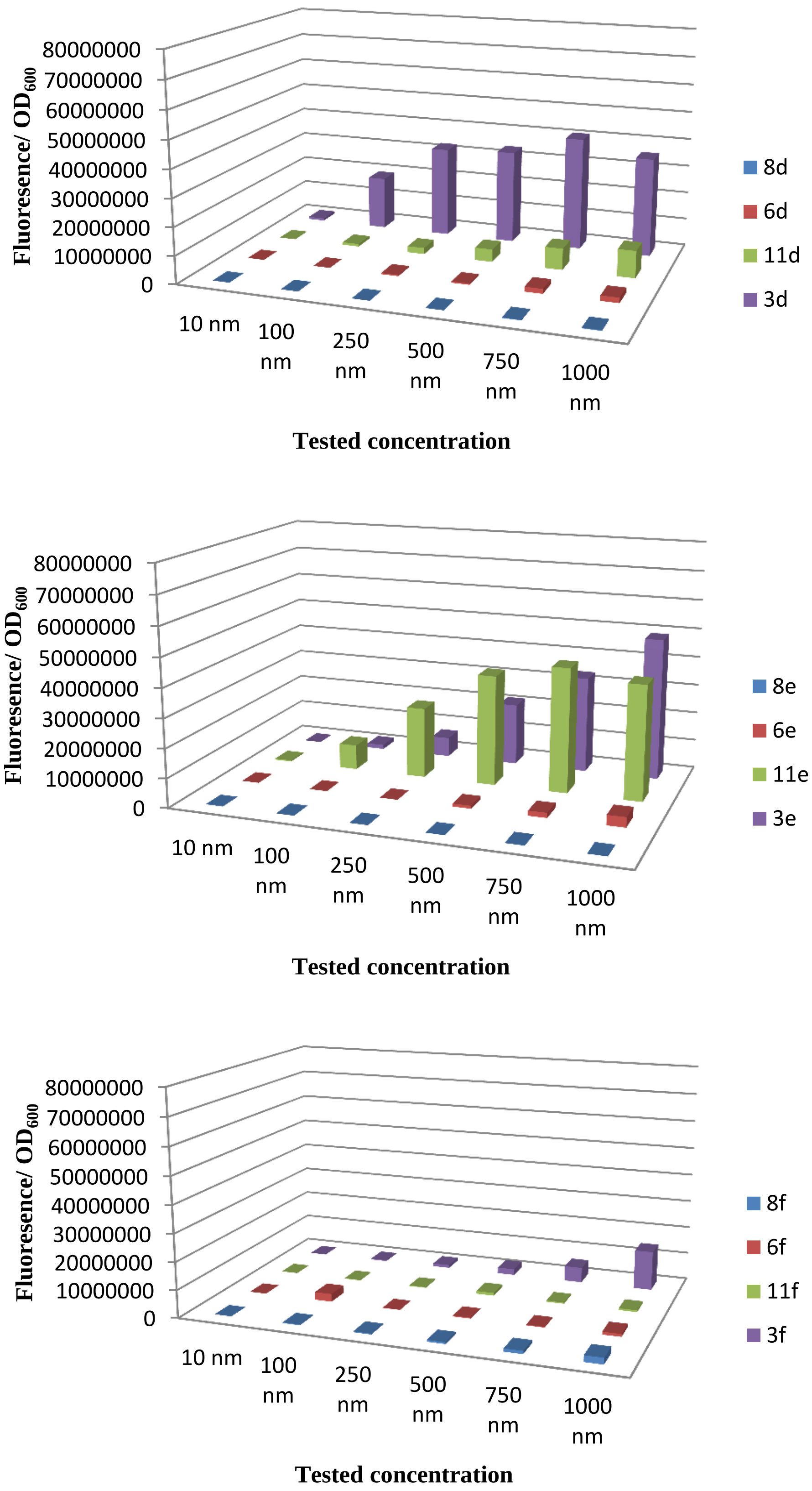

Figure S1: Normalized fluorescence values for natural AHLs **3c–d** and α-haloacylated analogues **6**, **8** and **11** tested in various concentrations. A; comparison of AHLs with chain length of **8** carbons. B; comparison of AHLs with chain length of **9** carbons. **C**; comparison of AHLs with chain length of **10** carbons **D**; comparison of AHLs with chain length of **12** carbons.
