# Supplementary material for: Synthesis and biological evaluation of novel N-α-haloacylated homoserine lactones as quorum sensing modulators
Source: Beilstein J Org Chem. 2014 Oct 30;10:2539–49. doi: 10.3762/bjoc.10.265 (PMC4222389; doi:10.3762/bjoc.10.265)
Supplement: File 2 — 1H and 13C NMR data and spectra, IR, HRMS, optical rotation and melting point data, chromatographic separation and yields of synthesized compounds. [file Beilstein_J_Org_Chem-10-2539-s002.pdf]

Supporting Information File 2

for

Synthesis and biological evaluation of novel *N*-α-haloacylated homoserine lactones as quorum sensing modulators

Michail Syrpas, Ewout Ruysbergh, Christian V. Stevens, Norbert De Kimpe and Sven Mangelinckx*

Address: [1]Department of Sustainable Organic Chemistry and Technology, Ghent University, Coupure Links 653, B-9000 Ghent, Belgium

Email: Sven Mangelinckx - Sven.Mangelinckx@Ugent.be

* Corresponding author

^{1}H and ^{13}C NMR data and spectra, IR, HRMS, optical rotation and melting point data, chromatographic separation and yields of synthesized compounds.

Characterization of novel compounds

N-(2-Bromoheptanoyl)-(*S*)-homoserine lactone (6b)

(IUPAC: 2-bromo-*N*-[(3*S*)-tetrahydro-2-oxo-3-furanyl]heptanamide)

^{1}H NMR (300 MHz, $CDCl_3$): δ 0.89 (3H, t, J=6.7 Hz, C<u>H</u>$_{3,\text{isomer 1 and 2}}$); 1.24-1.55 (6H, m, (C<u>H</u>$_2$)$_3$CH$_{3,\text{ isomer 1 and 2}}$); 1.94-2.05 (1H, m, C<u>H</u>H'CHBr$_{\text{isomer 1 and 2}}$); 2.05-2.16 (1H, m, CH<u>H</u>'CHBr$_{\text{isomer 1 and 2}}$); 2.16-2.30 (1H, m, OCH$_2$C<u>H</u>H'$_{\text{isomer 1 and 2}}$); 2.79-2.90 (1H, m, OCH$_2$CH<u>H</u>'$_{\text{isomer 1 and 2}}$); 4.27-4.36 (2H, m, C<u>H</u>Br$_{\text{isomer 1 and 2}}$ and OCH<u>H</u>'$_{\text{isomer 1 and 2}}$); 4.48-4.53 (0.5H, m, C<u>H</u>N$_{\text{isomer 1}}$); 4.50 (1H, t, J=8.7 Hz, OC<u>H</u>H'$_{\text{isomer 1 and 2}}$); 4.51-4.60 (0.5H, m, C<u>H</u>N$_{\text{isomer 2}}$); 6.92 (1H, d, J=5.5 Hz, N<u>H</u>$_{\text{isomer 1 and 2}}$). **^{13}C NMR (75 MHz, $CDCl_3$): δ** 14.0 (<u>C</u>H$_{3,\text{ isomer 1 and 2}}$); 22.5; 26.9; 27.0 and 31.0 ((<u>C</u>H$_2$)$_3$CH$_{3,\text{ isomer 1 and 2}}$); 30.0 (<u>C</u>H$_2CHN_{\text{isomer 1 and 2}}$); 35.61 (<u>C</u>H$_2$CHBr$_{\text{isomer 1}}$); 35.68 (<u>C</u>H$_2$CHBr$_{\text{isomer 2}}$); 49.8 (<u>C</u>HN$_{\text{isomer 1 and 2}}$); 50.4 (<u>C</u>HBr$_{\text{isomer 1}}$); 50.6 (<u>C</u>HBr$_{\text{isomer 2}}$); 66.12 (<u>C</u>H$_2O_{\text{isomer 1}}$); 66.15 (<u>C</u>H$_2O_{\text{isomer 2}}$); 169.71 (N<u>C</u>=O$_{\text{isomer 1}}$); 169.73 (N<u>C</u>=O$_{\text{isomer 2}}$); 174.75 (O<u>C</u>=O$_{\text{isomer 1}}$); 174.84 (O<u>C</u>=O$_{\text{isomer 2}}$). **MS (ESI): m/z (%):** 292/294 (M+H$^+$, 100). **HRMS mass calculated:** $C_{11}H_{18}BrNO_3H^+$ 292.0537; **obtained:** 292.0536. **IR (cm^{-1}): v_{max}** 1014; 1171 (C-O); 1551 (HN-C=O); 1654 (HN-C=O); 1772 (C=O$_{\text{lactone}}$); 2854 (CH); 2925 (CH); 2952 (CH); 3295 (NH). **Chromatography:** EtOAc:PE 4:1 R_f = 0.49. **Melting Point:** 152°C. White powder. **Y:** 40%.

***N*-(2-Bromooctanoyl)-(*S*)-homoserine lactone (6c)**

(IUPAC: 2-bromo-*N*-[(3*S*)-tetrahydro-2-oxo-3-furanyl]octanamide)

^{1}H NMR (300 MHz, $CDCl_3$): δ 0.88 (3H, t, J=6.9 Hz, C<u>H</u>$_{3,\text{isomer 1 and 2}}$); 1.21-1.54 (8H, m, (C<u>H</u>$_2$)$_4$CH$_{3,\text{ isomer 1 and 2}}$); 1.94-2.06 (1H, m, C<u>H</u>H'CHBr$_{\text{isomer 1 and 2}}$); 2.06-2.15 (1H, m, CH<u>H</u>'CHBr$_{\text{isomer 1 and 2}}$); 2.15-2.29 (1H, m, OCH$_2$C<u>H</u>H'$_{\text{isomer 1 and 2}}$); 2.80-2.91 (1H, m, OCH$_2$CH<u>H</u>'$_{\text{isomer 1 and 2}}$); 4.26-4.36 (2H, m, C<u>H</u>Br$_{\text{isomer 1 and 2}}$ and OCH<u>H</u>'$_{\text{isomer 1 and 2}}$); 4.50 (1H, t, J=8.8 Hz, OC<u>H</u>H'$_{\text{isomer 1 and 2}}$); 4.49-4.60 (1H, m, C<u>H</u>N$_{\text{isomer 1 and 2}}$); 6.83 (1H, d,

J=6.83 Hz, $N\underline{H}_{\text{isomer 1 and 2}}$). **^{13}C NMR (75 MHz, $CDCl_3$): δ** 14.1 ($\underline{C}H_{3,\text{ isomer 1 and 2}}$); 22.6; 27.1; 27.2; 28.5 and 31.6 ($(\underline{C}H_2)_4CH_{3,\text{isomer 1 and 2}}$); 30.1 ($\underline{C}H_2CHN_{\text{isomer 1 and 2}}$); 35.67 ($\underline{C}H_2CHBr_{\text{isomer 1}}$); 35.73 ($\underline{C}H_2CHBr_{\text{isomer 2}}$); 49.8 ($\underline{C}HN_{\text{isomer 1 and 2}}$); 50.5 ($\underline{C}HBr_{\text{isomer 1}}$); 50.7 ($\underline{C}HBr_{\text{isomer 2}}$); 66.12 ($\underline{C}H_2O_{\text{isomer 1}}$); 66.15 ($\underline{C}H_2O_{\text{isomer 2}}$); 169.7 ($N\underline{C}{=}O_{\text{isomer 1}}$); 169.8 ($N\underline{C}{=}O_{\text{isomer 2}}$); 174.7 ($O\underline{C}{=}O_{\text{isomer 1}}$); 174.8 ($O\underline{C}{=}O_{\text{isomer 2}}$). **MS (ESI): m/z (%):** 306/308 ($M+H^+$, 100). **HRMS mass calculated:** $C_{12}H_{20}BrNO_3H^+$ 306.0693; **obtained:** 306.0691. **IR (cm^{-1}): ν_{max}** 1016; 1181 (C-O); 1552 (HN-C=O); 1654 (HN-C=O); 1770 ($C{=}O_{\text{lactone}}$); 2854 (CH); 2924 (CH); 2955 (CH); 3295 (NH). **Chromatography:** EtOAc:PE 4:1 R_f = 0.51. **Melting Point:** 154°C. White powder. **Y:** 44%.

***N*-(2-Bromononanoyl)-(*S*)-homoserine lactone (6d)**

(IUPAC: 2-bromo-*N*-[(3*S*)-tetrahydro-2-oxo-3-furanyl]nonanamide)

^{1}H NMR (300 MHz, $CDCl_3$): δ 0.88 (3H, t, J=6.9 Hz, $C\underline{H}_{3,\text{isomer 1 and 2}}$); 1.20-1.53 (10H, m, $(C\underline{H}_2)_5CH_{3,\text{isomer 1 and 2}}$); 1.94-2.06 (1H, m, $C\underline{H}H'CHBr_{\text{ isomer 1 and 2}}$); 2.06-2.15 (1H, m, $CH\underline{H}'CHBr_{\text{isomer 1 and 2}}$); 2.15-2.30 (1H, m, $OCH_2C\underline{H}H'_{\text{isomer 1 and 2}}$); 2.79-2.90 (1H, m, $OCH_2CH\underline{H}'_{\text{isomer 1 and 2}}$); 4.26-4.36 (2H, m, $C\underline{H}Br_{\text{isomer 1 and 2}}$ and $OCH\underline{H}'_{\text{isomer 1 and 2}}$); 4.50 (1H, t, J=9.2 Hz, $OC\underline{H}H'_{\text{isomer 1 and 2}}$); 4.52-4.61 (1H, m, $C\underline{H}N_{\text{isomer 1 and 2}}$) 6.95 (1H, d, J=5.5 Hz, $N\underline{H}_{\text{isomer 1 and 2}}$). **^{13}C NMR (75 MHz, $CDCl_3$): δ** 14.2 ($\underline{C}H_{3,\text{ isomer 1 and 2}}$); 22.7; 27.25; 27.31; 28.8; 29.1 and 31.8 ($(\underline{C}H_2)_5CH_{3,\text{ isomer 1 and 2}}$); 30.0 ($\underline{C}H_2CHN_{\text{isomer 1 and 2}}$); 35.6 ($\underline{C}H_2CHBr_{\text{isomer 1}}$); 35.7 ($\underline{C}H_2CHBr_{\text{isomer 2}}$); 49.8 ($\underline{C}HN_{\text{isomer 1 and 2}}$); 50.4 ($\underline{C}HBr_{\text{isomer 1}}$); 50.5 ($\underline{C}HBr_{\text{isomer 2}}$); 66.17 ($\underline{C}H_2O_{\text{isomer 1}}$); 66.20 ($\underline{C}H_2O_{\text{isomer 2}}$); 169.8 ($N\underline{C}{=}O_{\text{isomer 1}}$); 169.9 ($N\underline{C}{=}O_{\text{isomer 2}}$); 174.8 ($O\underline{C}{=}O_{\text{isomer 1}}$); 174.9 ($O\underline{C}{=}O_{\text{isomer 2}}$). **MS (ESI): m/z (%):** 320/322

(M+H$^+$, 100). **HRMS mass calculated:** $C_{13}H_{22}BrNO_3H^+$ 320.0850; **obtained:** 320.0848. **IR (cm^{-1})**: **v_{max}** 1014; 1180 (C-O); 1551 (HN-C=O); 1654 (HN-C=O); 1770 (C=$O_{lactone}$); 2852 (CH); 2922 (CH); 2954 (CH); 3304 (NH). **Chromatography:** EtOAc:PE 4:1 R_f = 0.54. **Melting Point:** 156°C. White powder. **Y:** 43%.

***N*-(2-Bromodecanoyl)-(*S*)-homoserine lactone (6e)**

(IUPAC: 2-bromo-*N*-[(3*S*)-tetrahydro-2-oxo-3-furanyl]decanamide)

^{1}H NMR (300 MHz, CDCl$_3$): δ 0.88 (3H, t, J=6.6 Hz, C$\underline{H}_{3,isomer\ 1\ and\ 2}$); 1.20-1.56 (12H, m, (C$\underline{H}_2$)$_6CH_{3,isomer\ 1\ and\ 2}$); 1.94-2.07 (1H, m, C$\underline{H}$H'CHBr$_{isomer\ 1\ and\ 2}$); 2.07-2.15 (1H, m, CH$\underline{H}$'CHBr$_{isomer\ 1\ and\ 2}$); 2.15-2.30 (1H, m, OCH$_2C\underline{H}H'_{isomer\ 1\ and\ 2}$); 2.79-2.91 (1H, m, OCH$_2CH\underline{H}$'$_{isomer\ 1\ and\ 2}$); 4.24-4.36 (2H, m, C$\underline{H}Br_{isomer\ 1\ and\ 2}$ and OCH$\underline{H}$'$_{isomer\ 1\ and\ 2}$); 4.50 (1H, t, J=9.2 Hz, OC$\underline{H}$H'$_{isomer\ 1\ and\ 2}$); 4.52-4.60 (1H, m, C$\underline{H}$N$_{isomer\ 1\ and\ 2}$); 6.93 (1H, d, J=5.5 Hz, N$\underline{H}_{isomer\ 1\ and\ 2}$). **^{13}C NMR (75 MHz, CDCl$_3$): δ** 14.2 ($\underline{C}$H$_{3,\ isomer\ 1\ and\ 2}$); 22.7; 27.20; 27.25; 28.9; 29.2 and 31.9 (($\underline{C}$H$_2$)$_6$CH$_{3,\ isomer\ 1\ and\ 2}$); 30.0 ($\underline{C}$H$_2$CHN$_{isomer\ 1\ and\ 2}$); 35.65 ($\underline{C}$H$_2$CHBr$_{isomer\ 1}$); 35.71 ($\underline{C}$H$_2$CHBr$_{isomer\ 2}$); 49.8 ($\underline{C}$HN$_{isomer\ 1\ and\ 2}$); 50.4 ($\underline{C}$HBr$_{isomer\ 1}$); 50.5 ($\underline{C}$HBr$_{isomer\ 2}$); 66.15 ($\underline{C}$H$_2$O$_{isomer\ 1}$); 66.18 ($\underline{C}$H$_2$O$_{isomer\ 2}$); 169.8 (N$\underline{C}$=O$_{isomer\ 1}$); 169.9 (N$\underline{C}$=O$_{isomer\ 2}$); 174.8 (O$\underline{C}$=O$_{isomer\ 1}$); 174.9 (O$\underline{C}$=O$_{isomer\ 2}$). **MS (ESI): m/z (%):** 334/336 (M+H$^+$, 100). **HRMS mass calculated:** $C_{14}H_{24}BrNO_3H^+$ 334.1006; **obtained:** 334.1006. **IR (cm^{-1})**: **v_{max}** 1016; 1178 (C-O); 1550 (HN-C=O); 1659 (HN-C=O); 1771 (C=$O_{lactone}$); 2851 (CH); 2920 (CH); 2955 (CH); 3303 (NH). **Chromatography:** EtOAc:PE 4:1 R_f = 0.56. **Melting Point:** 154°C. White powder. **Y:** 39%.

***N*-(2-Bromododecanoyl)-(*S*)-homoserine lactone (6f)**

(IUPAC: 2-bromo-*N*-[(3*S*)-tetrahydro-2-oxo-3-furanyl]dodecanamide)

^{1}H NMR (300 MHz, $CDCl_3$): δ 0.88 (3H, t, J=6.6 Hz, C$\underline{H}_{3,isomer\ 1\ and\ 2}$); 1.20-1.54 (16H, m, (C$\underline{H}_2$)$_8CH_{3,isomer\ 1\ and\ 2}$); 1.94-2.14 (2H, m, C$\underline{H}_2$CHBr,$_{isomer\ 1\ and\ 2}$); 2.14-2.29 (1H, m, OCH$_2C\underline{H}H'_{isomer\ 1\ and\ 2}$); 2.80-2.91 (1H, m, OCH$_2CH\underline{H}$'$_{isomer\ 1\ and\ 2}$); 4.21-4.36 (2H, m, C$\underline{H}Br_{isomer\ 1\ and\ 2}$ and OCH$\underline{H}$'$_{isomer\ 1\ and\ 2}$); 4.50 (1H, t, J=9.2 Hz, OC$\underline{H}$H'$_{isomer\ 1\ and\ 2}$); 4.51-4.60 (1H, m, C$\underline{H}$N$_{isomer\ 1\ and\ 2}$); 6.92 (1H, d, J=6.1 Hz, N$\underline{H}_{isomer\ 1\ and\ 2}$). **^{13}C NMR (75 MHz, $CDCl_3$): δ** 14.3 ($\underline{C}$H$_{3,\ isomer\ 1\ and\ 2}$); 22.8; 27.26; 27.31; 28.9; 29.4; 29.59; 29.63 and 32.0 (($\underline{C}$H$_2$)$_8$CH$_{3,isomer\ 1\ and\ 2}$); 30.0 ($\underline{C}$H$_2$CHN$_{isomer\ 1\ and\ 2}$); 35.5 ($\underline{C}$H$_2$CHBr$_{isomer\ 1\ and\ 2}$); 49.9 ($\underline{C}$HN$_{isomer\ 1\ and\ 2}$); 50.5 ($\underline{C}$HBr$_{isomer\ 1}$); 50.6 ($\underline{C}$HBr$_{isomer\ 2}$); 66.2 ($\underline{C}$H$_2$O$_{isomer\ 1}$); 66.3 ($\underline{C}$H$_2$O$_{isomer\ 2}$); 169.88 (N$\underline{C}$=O$_{isomer\ 1}$); 169.94 (N$\underline{C}$=O$_{isomer\ 1}$); 174.86 (O$\underline{C}$=O$_{isomer\ 1}$); 174.94 (O$\underline{C}$=O$_{isomer\ 2}$). **MS (ESI): m/z (%):** 362/364 (M+H$^+$, 100). **HRMS mass calculated:** $C_{16}H_{28}BrNO_3H^+$ 362.1319; **obtained:** 362.1319 **IR (cm^{-1})**: **v_{max}** 1016; 1179 (C-O); 1550 (HN-C=O); 1652 (HN-C=O); 1771 (C=O$_{lactone}$); 2851 (CH); 2920 (CH); 2954 (CH); 3302 (NH). **Chromatography:** EtOAc:PE 4:1 R_f = 0.58. **Melting Point:** 148°C. White powder. **Y:** 38%.

***N*-(2-Iodoheptanoyl)-(*S*)-homoserine lactone (8b)**

(IUPAC: 2-iodo-*N*-[(3*S*)-tetrahydro-2-oxo-3-furanyl]heptanamide)

^{1}H NMR (300 MHz, $CDCl_3$): δ 0.89 (3H, t, J=7.2 Hz, C$\underline{H}_{3,\ isomer\ 1\ and\ 2}$); 1.23-1.48 (6H, m, (C$\underline{H}_2$)$_3CH_{3,\ isomer\ 1\ and\ 2}$); 1.99 (2H, q, J=7.3 Hz, C$\underline{H}_2$CHI$_{isomer\ 1\ and\ 2}$); 2.14-2.29 (1H, m, OCH$_2$C$\underline{H}$H'$_{isomer\ 1\ and\ 2}$); 2.80-2.93 (1H, m, OCH$_2$CH$\underline{H}$'$_{isomer\ 1\ and\ 2}$); 4.27-4.36 (1H, m, OCH$\underline{H}$'$_{isomer\ 1\ and\ 2}$); 4.32 (1H, t, J=7.2 Hz, C$\underline{H}$I$_{isomer\ 1\ and\ 2}$); 4.51 (1H, t, J=8.8 Hz, OC$\underline{H}$H'$_{isomer\ 1\ and\ 2}$); 4.49-4.56 (0.5H, m, C$\underline{H}$N$_{isomer\ 1}$); 4.62 (0.5H, ddd, J=11.7 Hz, 8.7 Hz, 6.1 Hz, C$\underline{H}$N$_{isomer\ 2}$); 6.55 (0.5H, d, J=6.1 Hz, N$\underline{H}_{isomer\ 1}$); 6.63 (0.5H, d, J=6.1 Hz,

N$\underline{H}_{isomer\ 2}$). **^{13}C NMR (75 MHz, $CDCl_3$): δ** 14.0 ($\underline{C}H_{3,\ isomer\ 1\ and\ 2}$); 22.5; 29.1 and 30.9 (($\underline{C}H_2)_3CH_{3,\ isomer\ 1\ and\ 2}$); 24.97 ($\underline{C}HI_{isomer\ 1}$); 25.02 ($\underline{C}HI_{isomer\ 2}$); 29.7 ($\underline{C}H_2CHN_{isomer\ 1}$); 29.8 ($\underline{C}H_2CHN_{isomer\ 2}$); 36.4 ($\underline{C}H_2CHI_{isomer\ 1}$); 36.6 ($\underline{C}H_2CHI_{isomer\ 2}$); 49.6 ($\underline{C}HN_{isomer\ 1}$); 49.8 ($\underline{C}HN_{isomer\ 2}$); 66.2 ($\underline{C}H_2O_{isomer\ 1\ and\ 2}$); 171.3 (N$\underline{C}$=$O_{isomer\ 1}$); 171.5 (N$\underline{C}$=$O_{isomer\ 2}$); 175.2 (O$\underline{C}$=$O_{isomer\ 1}$); 175.3 (O$\underline{C}$=$O_{isomer\ 2}$). **MS (ESI): m/z (%):** 340 (M+H$^+$, 100). **HRMS mass calculated:** $C_{11}H_{18}INO_3H^+$ 340.0409; **obtained:** 340.0405. **IR (cm^{-1}): ν_{max}** 1010; 1184 (C-O); 1557 (HN-C=O); 1641 (HN-C=O); 1765 (C=$O_{lactone}$); 2854 (CH); 2925 (CH); 2953 (CH); 3298 (NH). **Chromatography:** EtOAc:PE 4:1 R_f = 0.58. **Melting Point:** 150°C. Yellowish powder. **Y:** 66%.

N-(2-Iodooctanoyl)-(*S*)-homoserine lactone (8c)

(IUPAC: 2-iodo-*N*-[(3*S*)-tetrahydro-2-oxo-3-furanyl]octanamide)

^{1}H NMR (300 MHz, $CDCl_3$): δ 0.88 (3H, t, J=7.2 Hz, $C\underline{H}_{3,\ isomer\ 1\ and\ 2}$); 1.18-1.51 (8H, m, ($C\underline{H}_2)_4CH_{3,\ isomer\ 1\ and\ 2}$); 1.99 (2H, q, J=7.2 Hz, $C\underline{H}_2CHI_{isomer\ 1\ and\ 2}$); 2.10-2.27 (1H, m, $OCH_2C\underline{H}H'_{isomer\ 1\ and\ 2}$); 2.83-2.94 (1H, m, $OCH_2CH\underline{H}'_{isomer\ 1\ and\ 2}$); 4.26-4.35 (1H, m, $OCH\underline{H}'_{isomer\ 1\ and\ 2}$); 4.30 (1H, t, J=7.2 Hz, $C\underline{H}I_{isomer\ 1\ and\ 2}$); 4.43-4.53 (0.5H, m, $C\underline{H}N_{isomer\ 1}$); 4.51 (1H, t, J=8.8 Hz, $OC\underline{H}H'_{isomer\ 1\ and\ 2}$); 4.59 (0.5H, ddd, J=11.6 Hz, 8.8 Hz, 6.1 Hz, $C\underline{H}N_{isomer\ 2}$); 6.36 (0.5H, d, J=6.1 Hz, $N\underline{H}_{isomer\ 1}$); 6.43 (0.5H, d, J=6.1 Hz, $N\underline{H}_{isomer\ 2}$). **^{13}C NMR (75 MHz, $CDCl_3$): δ** 14.1 ($\underline{C}H_{3,\ isomer\ 1\ and\ 2}$); 22.6; 28.5; 29.4 and 31.6 (($\underline{C}H_2)_4CH_{3,\ isomer\ 1\ and\ 2}$); 24.97 ($\underline{C}HI_{isomer\ 1}$); 25.01 ($\underline{C}HI_{isomer\ 2}$); 29.6 ($\underline{C}H_2CHN_{isomer\ 1}$); 29.7 ($\underline{C}H_2CHN_{isomer\ 2}$); 36.4 ($\underline{C}H_2CHI_{isomer\ 1}$); 36.6 ($\underline{C}H_2CHI_{isomer\ 2}$); 49.6 ($\underline{C}HN_{isomer\ 1}$); 49.8 ($\underline{C}HN_{isomer\ 2}$); 66.3 ($\underline{C}H_2O_{isomer\ 1\ and\ 2}$); 171.4 (N$\underline{C}$=$O_{isomer\ 1}$); 171.5 (N$\underline{C}$=$O_{isomer\ 2}$); 175.3

(O$\underline{C}$=$O_{isomer\ 1}$); 175.4 (O$\underline{C}$=$O_{isomer\ 2}$). **MS (ESI): m/z (%):** 354 (M+H^+, 100). **HRMS mass calculated:** $C_{12}H_{20}INO_3H^+$ 354.0566; **obtained**: 354.0559. **IR (cm^{-1}): ν_{max}** 1016; 1177 (C-O); 1547 (HN-C=O); 1650 (HN-C=O); 1771 (C=$O_{lactone}$); 2854 (CH); 2923 (CH); 2956 (CH); 3292 (NH). **Chromatography:** EtOAc:PE 4:1 R_f = 0.54. **Melting Point:** 148°C. Yellowish powder. **Y:** 44%.

***N*-(2-Iodononanoyl)-(*S*)-homoserine lactone (8d)**

(IUPAC: 2-iodo-*N*-[(3*S*)-tetrahydro-2-oxo-3-furanyl]nonanamide)

^{1}H NMR (300 MHz, $CDCl_3$): δ 0.88 (3H, t, J=7.2 Hz, C$\underline{H}_{3,\ isomer\ 1\ and\ 2}$); 1.16-1.46 (10H, m, (C$\underline{H}_2$)$_5$$CH_{3,isomer\ 1\ and\ 2}$); 1.99 (2H, q, J=7.2 Hz, C$\underline{H}_2$CHI,$_{isomer\ 1\ and\ 2}$); 2.13-2.30 (1H, m, OCH_2C$\underline{H}$H',$_{isomer\ 1\ and\ 2}$); 2.78-2.91 (1H, m, OCH_2CH$\underline{H}$',$_{isomer\ 1\ and\ 2}$); 4.27-4.37 (1H, m, OCH$\underline{H}$',$_{isomer\ 1\ and\ 2}$); 4.32 (1H, t, J=7.2 Hz, C$\underline{H}$$I_{isomer\ 1\ and\ 2}$); 4.49-4.57 (0.5H, m, C$\underline{H}$$N_{isomer\ 1}$); 4.51 (1H, t, J=8.8 Hz, OC$\underline{H}$$H'_{isomer\ 1\ and\ 2}$); 4.63 (0.5H, ddd, J=11.8 Hz, 8.5 Hz, 6.1 Hz, C$\underline{H}$$N_{isomer\ 2}$); 6.65 (0.5H, d, J=6.1 Hz, N$\underline{H}_{isomer\ 1}$); 6.73 (0.5H, d, J=6.1 Hz, N$\underline{H}_{isomer\ 2}$). **^{13}C NMR (75 MHz, $CDCl_3$): δ** 14.2 ($\underline{C}H_{3,\ isomer\ 1\ and\ 2}$); 22.7; 28.8; 29.1; 29.5 and 31.8 (($\underline{C}H_2$)$_5$$CH_{3,\ isomer\ 1\ and\ 2}$); 25.00 ($\underline{C}$$HI_{isomer\ 1}$); 25.01 ($\underline{C}$$HI_{isomer\ 2}$); 29.7 ($\underline{C}H_2$$CHN_{isomer\ 1}$); 29.8 ($\underline{C}H_2$$CHN_{isomer\ 2}$); 36.4 ($\underline{C}H_2CH_2$$CHI_{isomer\ 1}$); 36.6 ($\underline{C}H_2CH_2$$CHI_{isomer\ 2}$); 49.7 ($\underline{C}$$HN_{isomer\ 1}$); 49.8 ($\underline{C}$$HN_{isomer\ 2}$); 66.3 ($\underline{C}H_2$$O_{isomer\ 1\ and\ 2}$); 171.3 (N$\underline{C}$=$O_{isomer\ 1}$); 171.5 (N$\underline{C}$=$O_{isomer\ 2}$); 175.2 (O$\underline{C}$=$O_{isomer\ 1}$); 175.3 (O$\underline{C}$=$O_{isomer\ 2}$). **MS (ESI): m/z (%):** 368 (M+H^+, 100). **HRMS mass calculated:** $C_{13}H_{22}INO_3H^+$ 368.0722; **obtained:** 368.0719. **IR (cm^{-1})**: **ν_{max}** 1014; 1178 (C-O); 1546 (HN-C=O); 1643 (HN-C=O); 1770 (C=$O_{lactone}$);

2852 (CH); 2923 (CH); 2953 (CH); 3289 (NH). **Chromatography:** EtOAc:PE 4:1 R_f = 0.58. **Melting Point:** 138°C. Yellowish powder. **Y:** 48%.

***N*-(2-Iododecanoyl)-(*S*)-homoserine lactone (8e)**

(IUPAC: 2-iodo-*N*-[(3*S*)-tetrahydro-2-oxo-3-furanyl]decanamide)

^{1}H NMR (300 MHz, $CDCl_3$): δ 0.88 (3H, t, J=7.2 Hz, $C\underline{H}_{3,\ isomer\ 1\ and\ 2}$); 1.18-1.46 (12H, m, $(C\underline{H}_2)_6CH_{3,\ isomer\ 1\ and}$); 1.99 (2H, q, J=7.2 Hz, $C\underline{H}_2CHI_{isomer\ 1\ and\ 2}$); 2.11-2.28 (1H, m, $OCH_2C\underline{H}H'_{isomer\ 1\ and\ 2}$); 2.81-2.94 (1H, m, $OCH_2CH\underline{H}'_{isomer\ 1\ and\ 2}$); 4.27-4.36 (1H, m, $OCH\underline{H}'_{isomer\ 1\ and\ 2}$); 4.31 (1H, t, J=7.4 Hz, $C\underline{H}I_{isomer\ 1\ and\ 2}$); 4.46-4.55 (0.5H, m, $C\underline{H}N_{isomer\ 1}$); 4.51 (1H, t, J=8.8 Hz, $OC\underline{H}H'_{isomer\ 1\ and\ 2}$); 4.60 (0.5H, ddd, J=11.6 Hz, 8.8 Hz, 6.1 Hz, $C\underline{H}N_{isomer\ 2}$); 6.45 (0.5H, d, J=6.1 Hz, $N\underline{H}_{isomer\ 1}$); 6.53 (0.5H, d, J=6.1 Hz, $N\underline{H}_{isomer\ 2}$). **^{13}C NMR (75 MHz, $CDCl_3$): δ** 14.2 ($\underline{C}H_{3,\ isomer\ 1\ and\ 2}$); 22.7; 28.81; 28.84; 29.4; 29.5 and 31.9 ($(\underline{C}H_2)_6CH_{3,\ isomer\ 1\ and\ 2}$); 24.9 ($\underline{C}HI_{isomer\ 1}$); 25.0 ($\underline{C}HI_{isomer\ 2}$); 29.8 ($\underline{C}H_2CHN_{isomer\ 1}$); 29.9 ($\underline{C}H_2CHN_{isomer\ 2}$); 36.5 ($\underline{C}H_2CHI_{isomer\ 1}$); 36.6 ($\underline{C}H_2CHI_{isomer\ 2}$); 49.7 ($\underline{C}HN_{isomer\ 1}$); 49.9 ($\underline{C}HN_{isomer\ 2}$); 66.2 ($\underline{C}H_2O_{isomer\ 1\ and\ 2}$); 171.3 ($N\underline{C}{=}O_{isomer\ 1}$); 171.4 ($N\underline{C}{=}O_{isomer\ 2}$); 175.1 ($O\underline{C}{=}O_{isomer\ 1}$); 175.2 ($O\underline{C}{=}O_{isomer\ 2}$). **MS (ESI): m/z (%):** 382 ($M+H^+$, 100). **HRMS mass calculated:** $C_{14}H_{24}INO_3H^+$ 382.0879; **obtained:** 382.0870. **IR (cm^{-1})**: $\mathbf{v_{max}}$ 1185 (C-O); 1542 (HN-C=O); 1644 (HN-C=O); 1769 ($C{=}O_{lactone}$); 2850 (CH); 2919 (CH); 2956 (CH); 3286 (NH). **Chromatography:** EtOAc:PE 3:2 R_f = 0.64. **Melting Point:** 142°C. Yellowish powder. **Y:** 59%.

***N*-(2-Iodododecanoyl)-(*S*)-homoserine lactone (8f)**

(IUPAC: 2-iodo-*N*-[(3*S*)-tetrahydro-2-oxo-3-furanyl]dodecanamide)

^{1}H NMR (300 MHz, $CDCl_3$): δ 0.88 (3H, t, J=7.2 Hz, C<u>H</u>$_{3, \text{isomer 1 and 2}}$); 1.16-1.47 (16H, m, $(C\underline{H}_2)_8CH3_{\text{, isomer 1 and 2}}$); 1.99 (2H, q, J=7.2 Hz, $C\underline{H}_2CHI_{\text{isomer 1 and 2}}$); 2.09-2.26 (1H, m, $OCH_2C\underline{H}H'_{\text{isomer 1 and 2}}$); 2.83-2.96 (1H, m, $OCH_2CH\underline{H}'_{\text{isomer 1 and 2}}$); 4.24-4.35 (1H, m, $OCH\underline{H}'_{\text{isomer 1 and 2}}$); 4.28 (1H, t, J=7.2 Hz, $C\underline{H}I_{\text{isomer 1 and 2}}$); 4.43-4.53 (0.5H, m, $C\underline{H}N_{\text{isomer 1}}$); 4.50 (1H, t, J=8.8 Hz, $OC\underline{H}H'_{\text{isomer 1 and 2}}$); 4.57 (0.5H, ddd, J=11.6 Hz, 8.8 Hz, 6.1 Hz, $C\underline{H}N_{\text{isomer 2}}$); 6.27 (0.5H, d, J=6.1 Hz, $N\underline{H}_{\text{isomer 1}}$); 6.34 (0.5H, d, J=6.1 Hz, $N\underline{H}_{\text{isomer 2}}$). **^{13}C NMR (75 MHz, $CDCl_3$): δ** 14.2 ($\underline{C}H_{3,\text{ isomer 1 and 2}}$); 22.8; 28.8; 29.4; 29.5; 29.58; 29.63; 29.8 and 32.0 ($(\underline{C}H_2)_8CH_{3,\text{ isomer 1 and 2}}$); 24.9 ($\underline{C}HI_{\text{isomer 1}}$); 25.0 ($\underline{C}HI_{\text{isomer 2}}$); 30.0 ($\underline{C}H_2CHN_{\text{isomer 1}}$); 30.1 ($\underline{C}H_2CHN_{\text{isomer 2}}$); 36.5 ($\underline{C}H_2CHI_{\text{isomer 1}}$); 36.7 ($\underline{C}H_2CHI_{\text{isomer 2}}$); 49.8 ($\underline{C}HN_{\text{isomer 1}}$); 50.0 ($\underline{C}HN_{\text{isomer 2}}$); 66.2 ($\underline{C}H_2O_{\text{isomer 1 and 2}}$); 171.1 ($N\underline{C}{=}O_{\text{isomer 1}}$); 171.3 ($N\underline{C}{=}O_{\text{isomer 2}}$); 175.1 ($O\underline{C}{=}O_{\text{isomer 1 and 2}}$). **MS (ESI): m/z (%):** 410 ($M+H^+$, 100). **HRMS mass calculated:** $C_{16}H_{28}INO_3H^+$ 410.1192; **obtained:** 410.1183. **IR (cm^{-1})**: **v_{max}** 1181 (C-O); 1542 (HN-C=O); 1643 (HN-C=O); 1770 ($C{=}O_{\text{lactone}}$); 2850 (CH); 2919 (CH); 2955 (CH) 3287 (NH). **Chromatography:** EtOAc:PE 3:2 R_f = 0.61. **Melting Point:** 143°C. Yellowish powder. **Y:** 12%.

***N*-(2-Chloroheptanoyl)-(*S*)-homoserine lactone (11b)**

(IUPAC: 2-chloro-*N*-[(3*S*)-tetrahydro-2-oxo-3-furanyl]heptanamide)

^{1}H NMR (300 MHz, $CDCl_3$): δ 0.90 (3H, t, J=7.2 Hz, $C\underline{H}_{3,\text{isomer 1 and 2}}$); 1.26-1.35 (4H, m, $(C\underline{H}_2)_2CH_{3,\text{ isomer 1 and 2}}$); 1.43-1.54 (2H, m, $C\underline{H}_2(CH_2)_2CH_{3,\text{isomer 1 and 2}}$); 1.88-2.02 (1H, m, $C\underline{H}H'CHCl_{\text{isomer 1 and 2}}$); 2.05-2.16 (1H, m, $CH\underline{H}'CHCl_{\text{isomer 1 and 2}}$); 2.21 (1H, dddd, J=11.8 Hz, 11.8 Hz, 11.7 Hz, 8.8 Hz, $OCH_2C\underline{H}H'_{\text{isomer 1 and 2}}$); 2.80-2.89 (1H, m, $OCH_2CH\underline{H}'_{\text{isomer 1 and 2}}$); 4.26-4.40 (2H, m, $C\underline{H}Cl_{\text{isomer 1 and 2}}$ and $OCH\underline{H}'_{\text{isomer 1 and 2}}$); 4.48-4.53 (0.5H, m,

$C\underline{H}N_{isomer\ 1}$); 4.51 (1H, t, J=8.8 Hz, $OC\underline{H}H'_{isomer\ 1\ and\ 2}$); 4.56 (0.5H, ddd, J=11.3 Hz, 8.8 Hz, 6.1 Hz, $C\underline{H}N_{isomer\ 2}$); 7.05 (0.5H, d, J=6.1 Hz, $N\underline{H}_{isomer\ 1}$); 7.09 (0.5H, d, J=6.1 Hz, $N\underline{H}_{isomer\ 2}$). **^{13}C NMR (75 MHz, $CDCl_3$): δ** 14.0 ($\underline{C}H_{3,\ isomer\ 1\ and\ 2}$); 22.5; 25.5; 25.6 and 31.0 ($(\underline{C}H_2)_3CH_{3,isomer\ 1\ and\ 2}$); 29.8 ($\underline{C}H_2CHN_{isomer\ 1\ and\ 2}$); 35.4 ($\underline{C}H_2CHCl_{isomer\ 1\ and\ 2}$); 49.49 ($\underline{C}HN_{isomer\ 1}$); 49.54 ($\underline{C}HN_{isomer\ 2}$); 60.58 ($\underline{C}HCl_{isomer\ 1}$); 60.64 ($\underline{C}HCl_{isomer\ 2}$); 66.06 ($\underline{C}H_2O_{isomer\ 1}$); 66.11 ($\underline{C}H_2O_{isomer\ 2}$); 169.96 ($N\underline{C}{=}O_{isomer\ 1}$); 170.00 ($N\underline{C}{=}O_{isomer\ 2}$); 174.7 ($O\underline{C}{=}O_{isomer\ 1}$); 174.8 ($O\underline{C}{=}O_{isomer\ 2}$). **MS (ESI): m/z (%):** 248/250 ($M+H^+$, 100). **HRMS mass calculated:** $C_{11}H_{18}ClNO_3H^+$ 248.1053; **obtained:** 248.1045. **IR (cm^{-1})**: **ν_{max}** 1015; 1174 (C-O); 1549 (HN-C=O); 1652 (HN-C=O); 1770 ($C{=}O_{lactone}$); 2859 (CH); 2928 (CH); 2955 (CH); 3296 (NH). **Chromatography:** EtOAc:PE 4:1 R_f = 0.47. **Melting Point:** 148°C. White powder. **Y:** 44%.

***N*-(2-Chlorooctanoyl)-(*S*)-homoserine lactone (11c)**

(IUPAC: 2-chloro-*N*-[(3*S*)-tetrahydro-2-oxo-3-furanyl]octanamide)

^{1}H NMR (300 MHz, $CDCl_3$): δ 0.88 (3H, t, J=7.2 Hz, $C\underline{H}_{3,isomer\ 1\ and\ 2}$); 1.20-1.41 (6H, m, $(C\underline{H}_2)_3CH_{3,\ isomer\ 1\ and\ 2}$); 1.42-1.55 (2H, m, $C\underline{H}_2(CH_2)_3CH_{3,isomer\ 1\ and\ 2}$); 1.87-2.01 (1H, m, $C\underline{H}H'CHCl_{isomer\ 1\ and\ 2}$); 2.05-2.14 (1H, m, $CH\underline{H}'CHCl_{isomer\ 1\ and\ 2}$); 2.22 (1H, dddd, J=11.8 Hz, 11.8 Hz, 11.7 Hz, 8.8 Hz, $OCH_2C\underline{H}H'_{isomer\ 1\ and\ 2}$); 2.79-2.88 (1H, m, $OCH_2CH\underline{H}'_{isomer\ 1\ and\ 2}$); 4.27-4.40 (2H, m, $C\underline{H}Cl_{isomer\ 1\ and\ 2}$ and $OCH\underline{H}'_{isomer\ 1\ and\ 2}$); 4.50 (1H, t, J=8.8 Hz, $OC\underline{H}H'_{isomer\ 1\ and\ 2}$); 4.53-4.62 (1H, m, $C\underline{H}N_{isomer\ 1\ and\ 2}$); 7.08 (0.5H, d, J=6.1 Hz, $N\underline{H}_{isomer\ 1}$); 7.12 (0.5H, d, J=6.1 Hz, $N\underline{H}_{isomer\ 2}$). **^{13}C NMR (75 MHz, $CDCl_3$): δ** 14.1 ($\underline{C}H_{3,\ isomer\ 1\ and\ 2}$); 22.6; 25.77; 25.84; 28.5 and 31.5 ($(\underline{C}H_2)_4CH_{3,isomer\ 1\ and\ 2}$); 29.9 ($\underline{C}H_2CHN_{isomer\ 1}$); 30.0 ($\underline{C}H_2CHN_{isomer\ 2}$); 35.5 ($\underline{C}H_2CHCl_{isomer\ 1\ and\ 2}$); 49.5 ($\underline{C}HN_{isomer\ 1}$); 49.6 ($\underline{C}HN_{isomer\ 2}$);

60.6 ($\underline{C}HCl_{isomer\ 1}$); 60.7 ($\underline{C}HCl_{isomer\ 2}$); 66.07 ($\underline{C}H_2O_{isomer\ 1}$); 66.12 ($\underline{C}H_2O_{isomer\ 2}$); 170.1 ($N\underline{C}=O_{isomer\ 1\ and\ 2}$); 174.8 ($O\underline{C}=O_{isomer\ 1\ and\ 2}$). **MS (ESI): m/z (%):** 262/264 ($M+H^+$, 100). **HRMS mass calculated:** $C_{12}H_{20}ClNO_3H^+$ 262.1210; **obtained:** 262.1206. **IR (cm^{-1}): v_{max}** 1008; 1173 (C-O); 1552 (HN-C=O); 1656 (HN-C=O); 1770 ($C=O_{lactone}$); 2857 (CH); 2922 (CH); 2955 (CH); 3300 (NH). **Chromatography:** EtOAc:PE 4:1 R_f = 0.51. **Melting Point:** 146°C. White powder. **Y:** 35%.

***N*-(2-Chlorononanoyl)-(*S*)-homoserine lactone (11d)**

(IUPAC: 2-chloro-*N*-[(3*S*)-tetrahydro-2-oxo-3-furanyl]nonanamide)

^{1}H NMR (300 MHz, $CDCl_3$): δ 0.88 (3H, t, J=7.2 Hz, $C\underline{H}_{3,\ isomer\ 1\ and\ 2}$); 1.19-1.39 (8H, m, $(C\underline{H}_2)_4CH_{3,\ isomer\ 1\ and\ 2}$); 1.40-1.55 (2H, m, $C\underline{H}_2(CH_2)_4CH_{3,\ isomer\ 1\ and\ 2}$); 1.87-2.01 (1H, m, $C\underline{H}H'CHCl_{\ isomer\ 1\ and\ 2}$); 2.05-2.16 (1H, m, $CH\underline{H}'CHCl_{isomer\ 1\ and\ 2}$); 2.21 (1H, dddd, J=11.8 Hz, 11.8 Hz, 11.7 Hz, 8.8 Hz, $OCH_2C\underline{H}H'_{isomer\ 1\ and\ 2}$); 2.80-2.89 (1H, m, $OCH_2CH\underline{H}'_{isomer\ 1\ and\ 2}$); 4.26-4.40 (2H, m, $C\underline{H}Cl_{isomer\ 1\ and\ 2}$ and $OCH\underline{H}'_{isomer\ 1\ and\ 2}$); 4.49-4.61 (1H, m, $C\underline{H}N_{isomer\ 1\ and\ 2}$); 4.50 (1H, t, J=8.8 Hz, $OC\underline{H}H'_{isomer\ 1\ and\ 2}$); 7.05 (0.5H, d, J=6.1 Hz, $N\underline{H}_{isomer\ 1}$); 7.09 (0.5H, d, J=6.1 Hz, $N\underline{H}_{isomer\ 2}$). **^{13}C NMR (75 MHz, $CDCl_3$): δ** 14.1 ($\underline{C}H_{3,\ isomer\ 1\ and\ 2}$); 22.7; 25.8; 25.9; 28.9; 29.1 and 31.8 ($(\underline{C}H_2)_5CH_{3,isomer\ 1\ and\ 2}$); 29.9 ($\underline{C}H_2CHN_{isomer\ 1\ and\ 2}$); 35.5 ($\underline{C}H_2CHCl_{isomer\ 1\ and\ 2}$); 49.5 ($\underline{C}HN_{isomer\ 1}$); 49.6 ($\underline{C}HN_{isomer\ 2}$); 60.6 ($\underline{C}HCl_{isomer\ 1}$); 60.7 ($\underline{C}HCl_{isomer\ 2}$); 66.0 ($\underline{C}H_2O_{isomer\ 1}$); 66.1 ($\underline{C}H_2O_{isomer\ 2}$); 169.96 ($N\underline{C}=O_{isomer\ 1}$); 169.99 ($N\underline{C}=O_{isomer\ 2}$); 174.7 ($O\underline{C}=O_{isomer\ 1}$); 174.8 ($O\underline{C}=O_{isomer\ 2}$). **MS (ESI): m/z (%):** 276/278 ($M+H^+$, 100). **HRMS mass calculated:** $C_{13}H_{22}ClNO_3H^+$ 276.1366; **obtained:** 276.1362. **IR (cm^{-1})**: **v_{max}** 1014; 1173 (C-O); 1553 (HN-C=O); 1660 (HN-C=O); 1770 ($C=O_{lactone}$); 2853 (CH); 2922 (CH); 2954 (CH); 3304 (NH).

Chromatography: EtOAc:PE 4:1 R_f = 0.50. **Melting Point:** 149°C. White powder. **Y:** 36%.

N-(2-Chlorodecanoyl)-(*S*)-homoserine lactone (11e)

(IUPAC: 2-chloro-*N*-[(3*S*)-tetrahydro-2-oxo-3-furanyl]decanamide)

^{1}H NMR (300 MHz, CDCl$_3$): δ 0.88 (3H, t, J=7.2 Hz, C<u>H</u>$_{3,\text{ isomer 1 and 2}}$); 1.19-1.39 (10H, m, (C<u>H</u>$_2$)$_5$CH$_{3,\text{ isomer 1 and 2}}$); 1.40-1.55 (2H, m, C<u>H</u>$_2$(CH$_2$)$_5$CH$_{3,\text{ isomer 1 and 2}}$); 1.87-2.01 (1H, m, C<u>H</u>H'CHCl$_{\text{isomer 1 and 2}}$); 2.05-2.13 (1H, m, CH<u>H</u>'CHCl$_{\text{isomer 1 and 2}}$); 2.20 (1H, dddd, J=11.7 Hz, 11.7 Hz, 11.7 Hz, 8.8 Hz, OCH$_2$C<u>H</u>H'$_{\text{isomer 1 and 2}}$); 2.81-2.90 (1H, m, OCH$_2$CH<u>H</u>'$_{\text{isomer 1 and 2}}$); 4.26-4.40 (2H, m, C<u>H</u>Cl$_{\text{isomer 1 and 2}}$ and OCH<u>H</u>'$_{\text{isomer 1 and 2}}$); 4.50 (1H, t, J=8.8 Hz, OC<u>H</u>H'$_{\text{isomer 1 and 2}}$); 4.52-4.59 (1H, m, C<u>H</u>N$_{\text{isomer 1 and 2}}$); 7.02 (0.5H, d, J=6.1 Hz, N<u>H</u>$_{\text{isomer 1}}$); 7.05 (0.5H, d, J=6.1 Hz, N<u>H</u>$_{\text{isomer 2}}$). **^{13}C NMR (75 MHz, CDCl$_3$): δ** 14.2 (<u>C</u>H$_{3,\text{ isomer 1 and 2}}$); 22.7; 25.8; 25.9; 28.9; 29.4 and 31.9 ((<u>C</u>H$_2$)$_6$CH$_{3,\text{isomer 1 and 2}}$); 29.9 (<u>C</u>H$_2CHN_{\text{isomer 1 and 2}}$); 35.5 (<u>C</u>H$_2$CHCl$_{\text{isomer 1 and 2}}$); 49.5 (<u>C</u>HN$_{\text{isomer 1}}$); 49.6 (<u>C</u>HN$_{\text{isomer 2}}$); 60.6 (<u>C</u>HCl$_{\text{isomer 1}}$); 60.7 (<u>C</u>HCl$_{\text{isomer 2}}$); 66.0 (<u>C</u>H$_2O_{\text{isomer 1}}$); 66.1 (<u>C</u>H$_2O_{\text{isomer 2}}$); 169.95 (N<u>C</u>=O$_{\text{isomer 1}}$); 170.00 (N<u>C</u>=O$_{\text{isomer 2}}$); 174.7 (O<u>C</u>=O$_{\text{isomer 1}}$); 174.8 (O<u>C</u>=O$_{\text{isomer 2}}$). **MS (ESI): m/z (%):** 290/292 (M+H$^+$, 100). **HRMS mass calculated:** $C_{14}H_{24}ClNO_3H^+$ 290.1523; **obtained:** 290.1518. **IR (cm^{-1})**: **ν_{max}** 1008; 1175 (C-O); 1555 (HN-C=O); 1657 (HN-C=O); 1770 (C=O$_{\text{lactone}}$); 2850 (CH); 2920 (CH); 2955 (CH); 3301 (NH). **Chromatography:** EtOAc:PE 4:1 R_f = 0.50. **Melting Point:** 149°C. White powder. **Y:** 40%.

N-(2-Chlorododecanoyl)-(*S*)-homoserine lactone (11f)

(IUPAC: 2-chloro-*N*-[(3*S*)-tetrahydro-2-oxo-3-furanyl]dodecanamide)

^{1}H NMR (300 MHz, $CDCl_3$): δ 0.88 (3H, t, J=7.2 Hz, C$\underline{H}_{3,isomer\ 1\ and\ 2}$); 1.18-1.38 (14H, m, (C$\underline{H}_2$)$_7CH_{3,isomer\ 1\ and\ 2}$); 1.39-1.55 (2H, m, C$\underline{H}_2$(CH$_2$)$_7CH_{3,\ isomer\ 1\ and\ 2}$); 1.87-2.01 (1H, m, C$\underline{H}$H'CHCl$_{isomer\ 1\ and\ 2}$); 2.05-2.14 (1H, m, CH$\underline{H}$'CHCl$_{isomer\ 1\ and\ 2}$); 2.20 (1H, dddd, J=11.8 Hz, 11.8 Hz, 11.7 Hz, 8.8 Hz, OCH$_2$C$\underline{H}$H'$_{isomer\ 1\ and\ 2}$); 2.80-2.90 (1H, m, OCH$_2$CH$\underline{H}$'$_{isomer\ 1\ and\ 2}$); 4.26-4.40 (2H, m, C$\underline{H}$Cl$_{isomer\ 1\ and\ 2}$ and OCH$\underline{H}$'$_{isomer\ 1\ and\ 2}$); 4.50 (1H, t, J=8.8 Hz, OC$\underline{H}$H'$_{isomer\ 1\ and\ 2}$); 4.52-4.60 (1H, m, C$\underline{H}$N$_{isomer\ 1\ and\ 2}$); 7.01 (0.5H, d, J=6.1 Hz, N$\underline{H}_{isomer\ 1}$); 7.05 (0.5H, d, J=6.1 Hz, N$\underline{H}_{isomer\ 2}$). **^{13}C NMR (75 MHz, $CDCl_3$):δ** 14.2 ($\underline{C}$H$_{3,\ isomer\ 1\ and\ 2}$); 22.8; 25.9; 28.9; 29.39; 29.41; 29.60; 29.63 and 32.0 (($\underline{C}$H$_2$)$_8$CH$_{3,isomer\ 1\ and\ 2}$); 29.9 ($\underline{C}$H$_2$CHN$_{isomer\ 1\ and\ 2}$); 35.5 ($\underline{C}$H$_2$CHCl$_{isomer\ 1\ and\ 2}$); 49.5 ($\underline{C}$HN$_{isomer\ 1}$); 49.6 ($\underline{C}$HN$_{isomer\ 2}$); 60.6 ($\underline{C}$HCl$_{isomer\ 1}$); 60.7 ($\underline{C}$HCl$_{isomer\ 2}$); 66.06 ($\underline{C}$H$_2$O$_{isomer\ 1}$); 66.10 ($\underline{C}$H$_2$O$_{isomer\ 2}$); 170.0 (N$\underline{C}$=O$_{isomer\ 1\ and\ 2}$); 174.7 (O$\underline{C}$=O$_{isomer\ 1}$); 174.8 (O$\underline{C}$=O$_{isomer\ 2}$). **MS (ESI): m/z (%):** 318/320 (M+H$^+$, 100). **HRMS mass calculated:** $C_{16}H_{28}ClNO_3H^+$ 318.1836; **obtained:** 318.1827. **IR (cm^{-1})**: **ν_{max}** 1008; 1176 (C-O); 1553 (HN-C=O); 1660 (HN-C=O); 1770 (C=O$_{lactone}$); 2850 (CH); 2919 (CH); 2955 (CH); 3302 (NH). **Chromatography:** EtOAc:PE 4:1 R_f = 0.52. **Melting Point:** 150°C. White powder. **Y:** 39%.

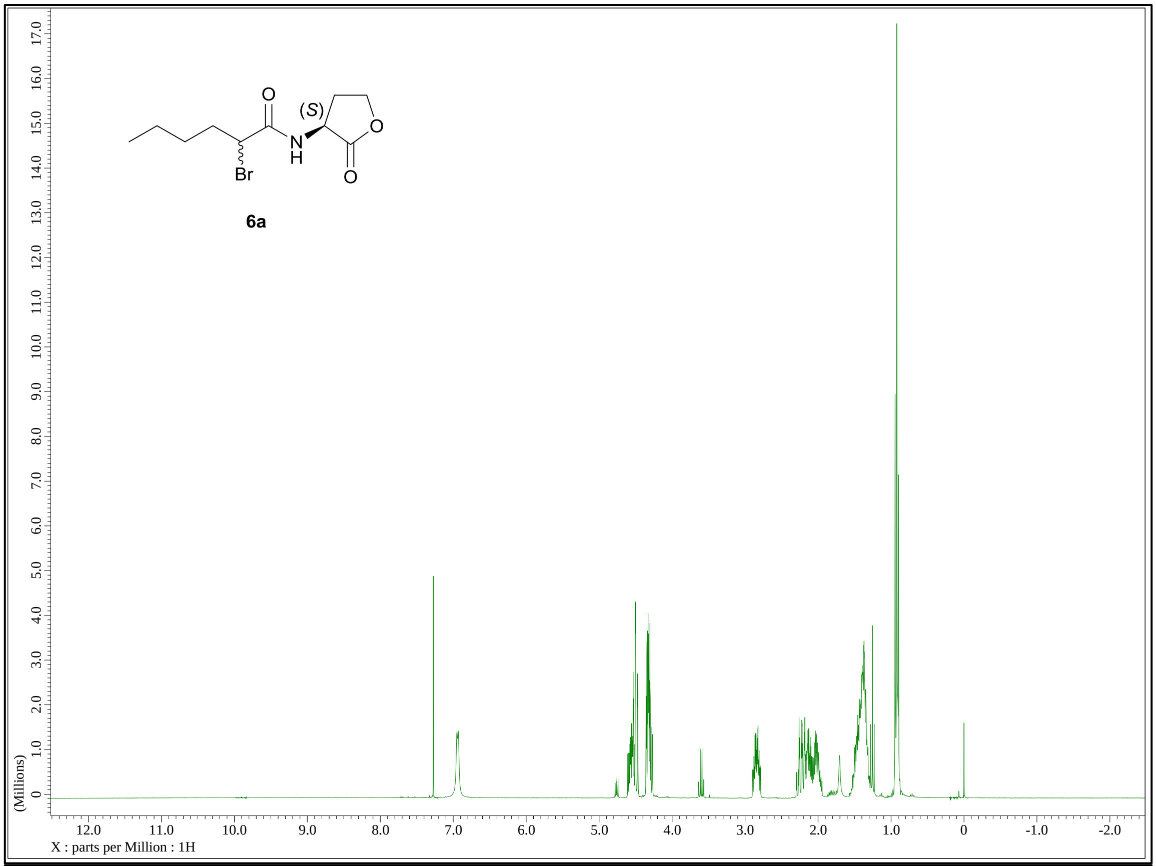

O
(S)
O
N
H
Br
O
6a
(Millions)
X : parts per Million : 1H

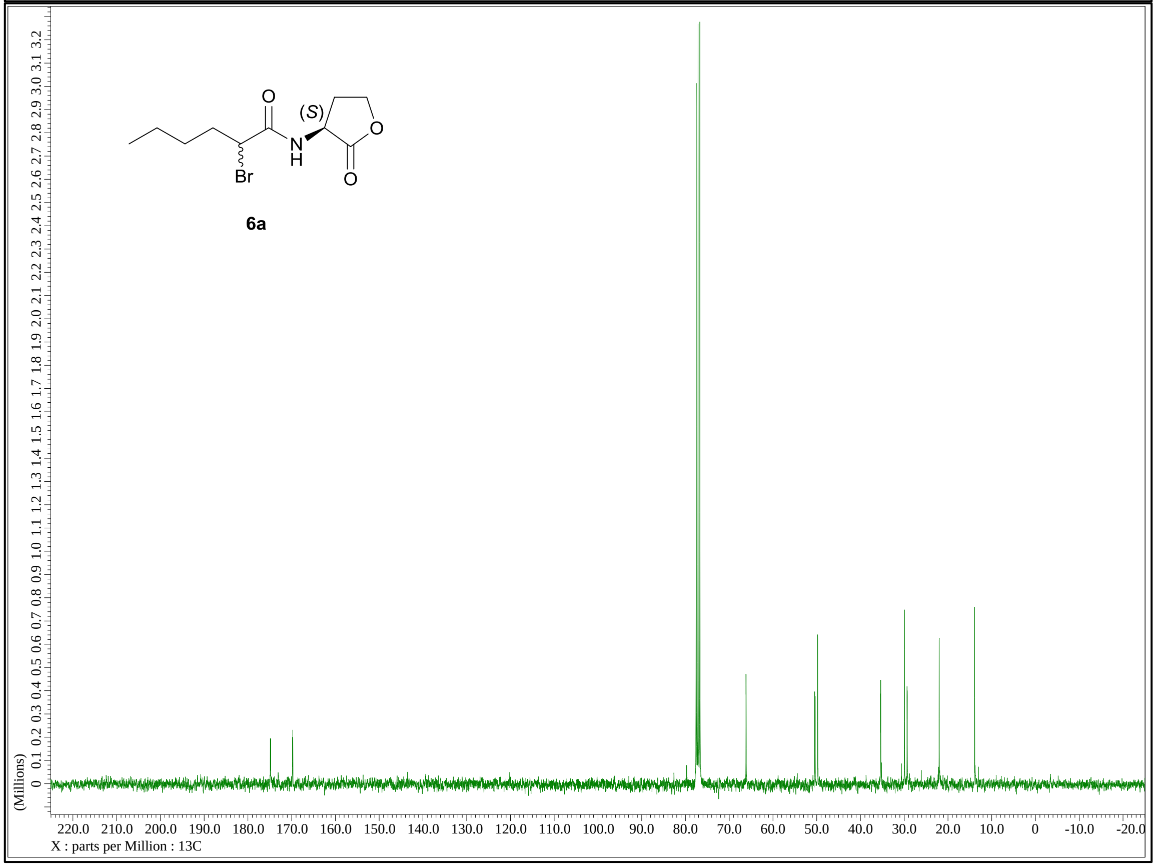

O
(S)
O
N
H
Br
O
6a
(Millions)
X : parts per Million : 13C

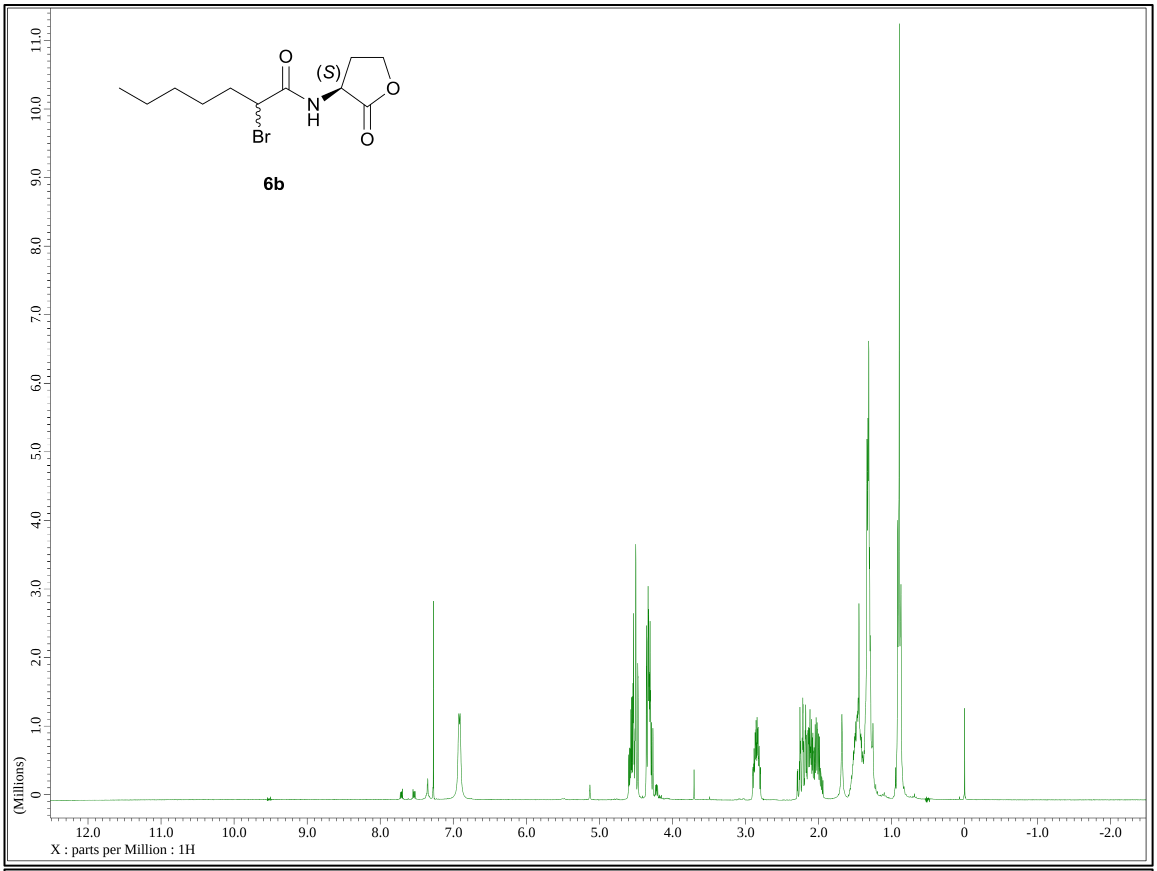
(S)
O
N
H
Br
6b
(Millions)
12.0 11.0 10.0 9.0 8.0 7.0 6.0 5.0 4.0 3.0 2.0 1.0 0 -1.0 -2.0
X : parts per Million : 1H

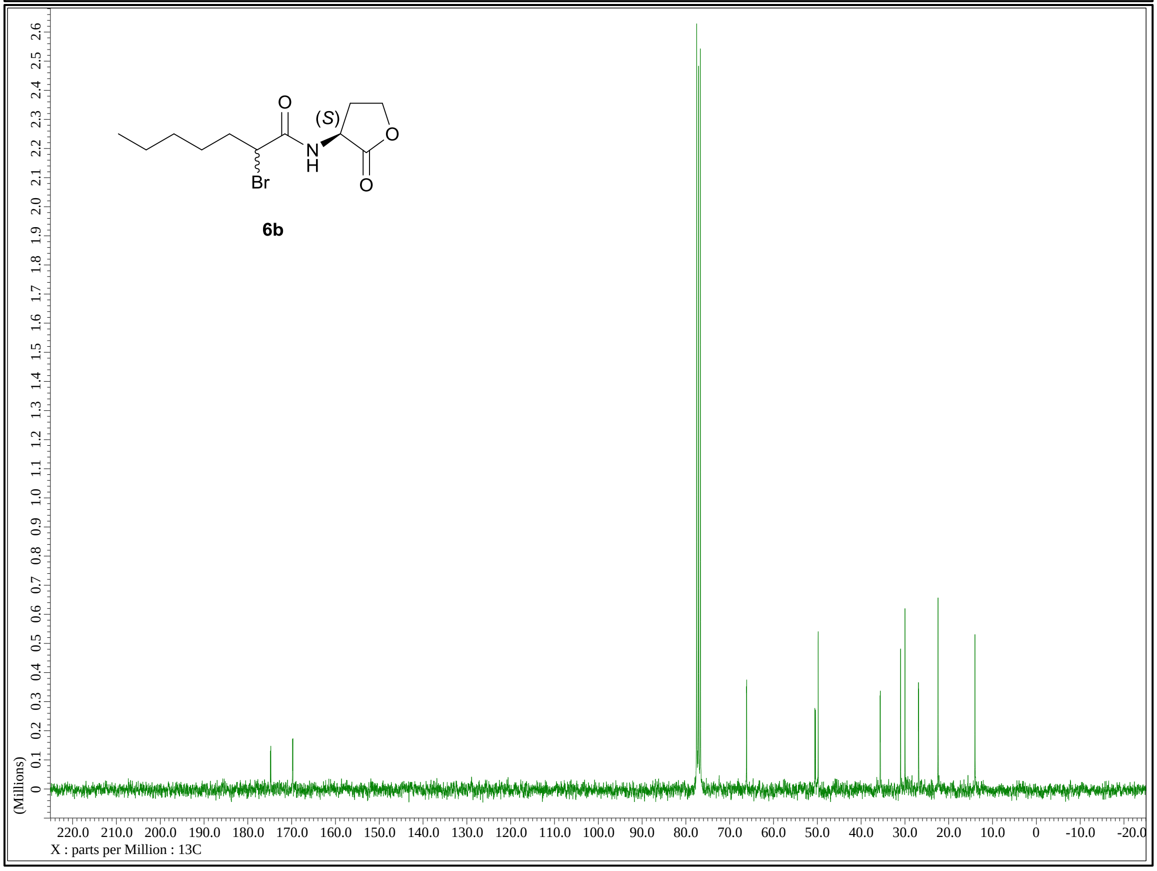
(S)
O
N
H
Br
6b
(Millions)
220.0 210.0 200.0 190.0 180.0 170.0 160.0 150.0 140.0 130.0 120.0 110.0 100.0 90.0 80.0 70.0 60.0 50.0 40.0 30.0 20.0 10.0 0 -10.0 -20.0
X : parts per Million : 13C

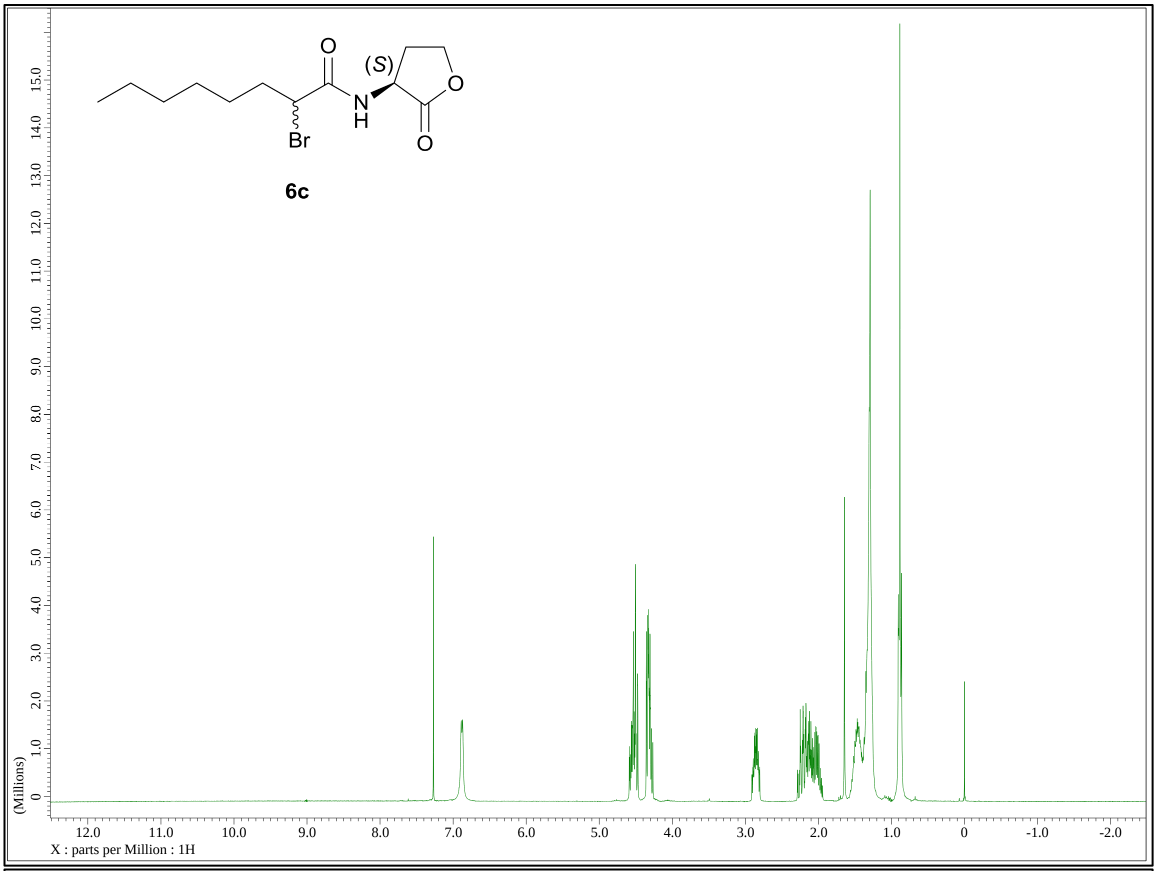
(S)
O
N
H
Br
O
O
6c
(Millions)
X : parts per Million : 1H

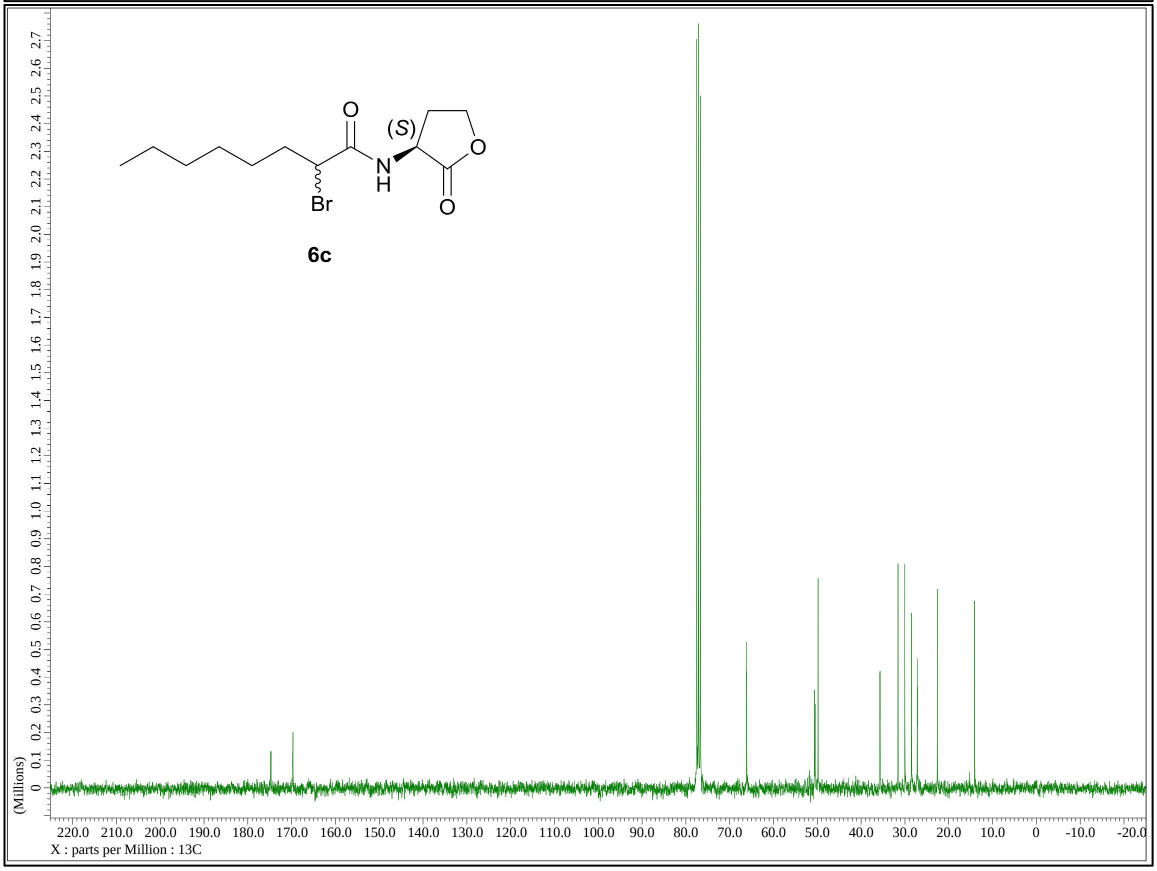
(S)
O
N
H
Br
O
O
6c
(Millions)
X : parts per Million : 13C

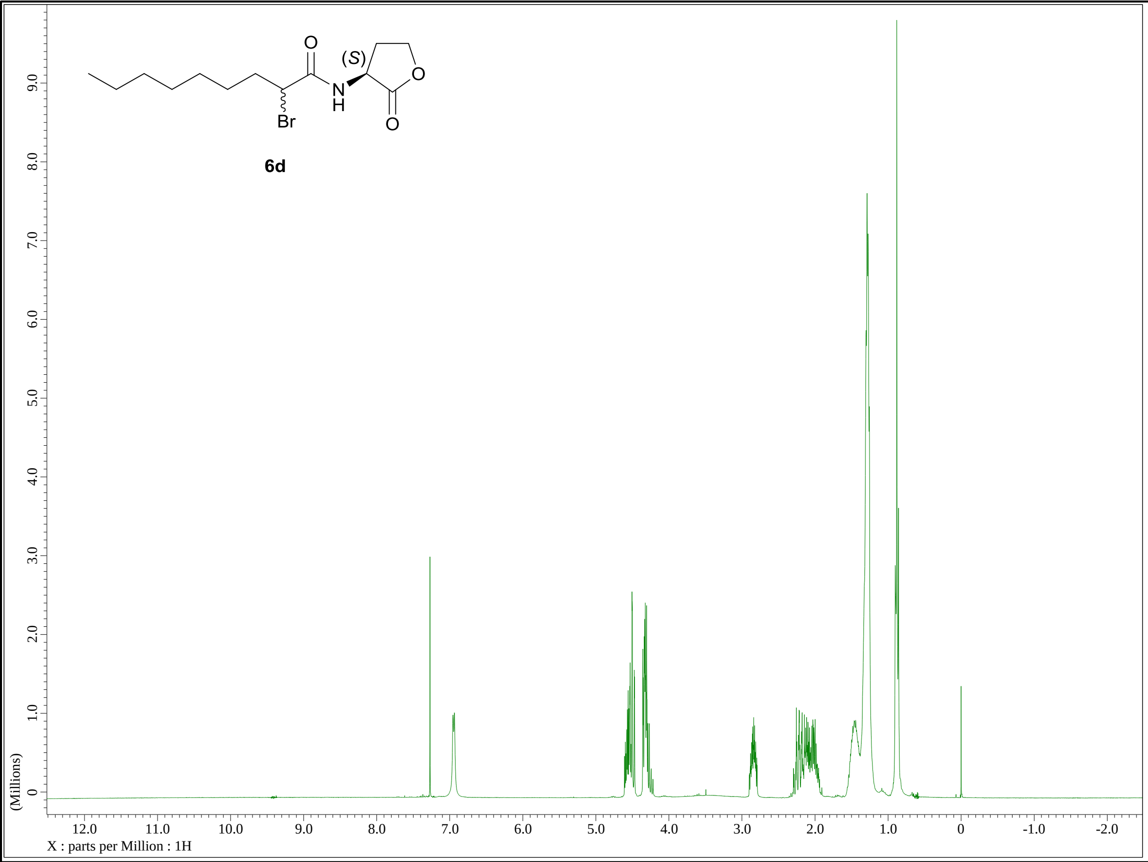

(S)
O
N
H
Br
6d
(Millions)
X : parts per Million : 1H

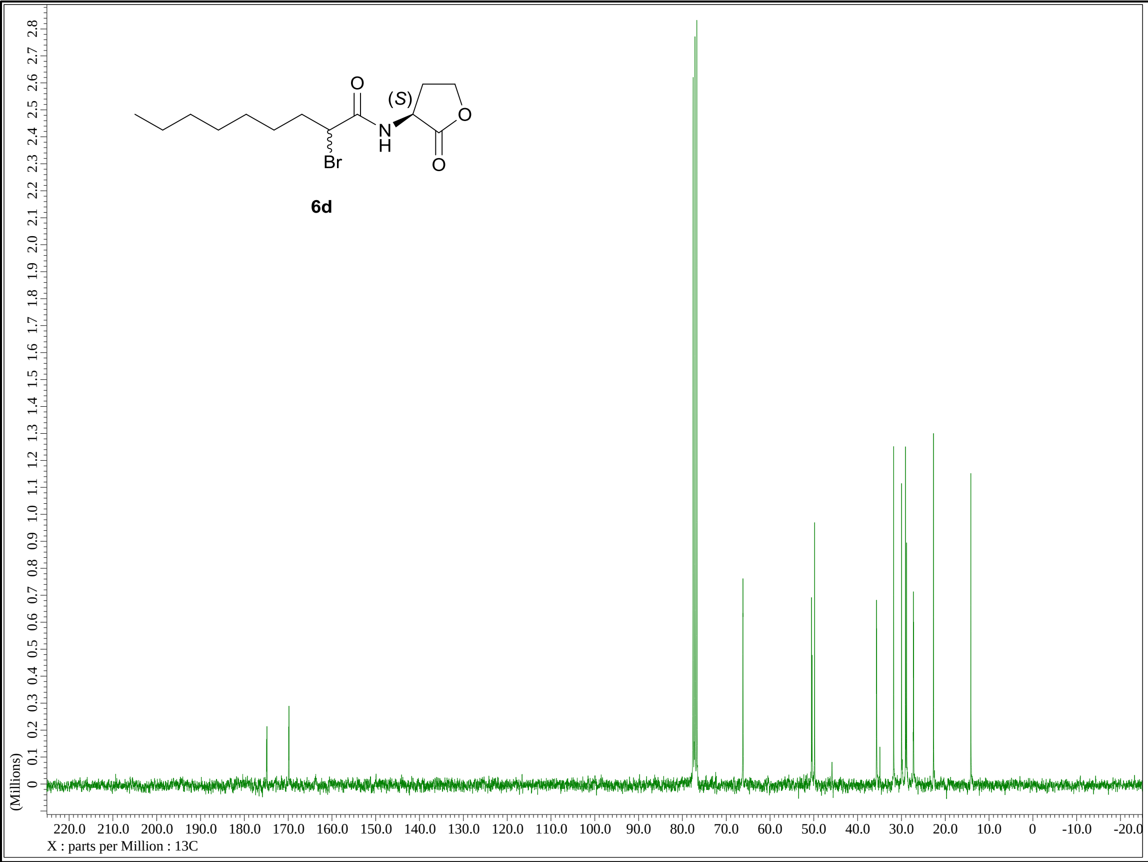

(S)
O
N
H
Br
6d
(Millions)
X : parts per Million : 13C

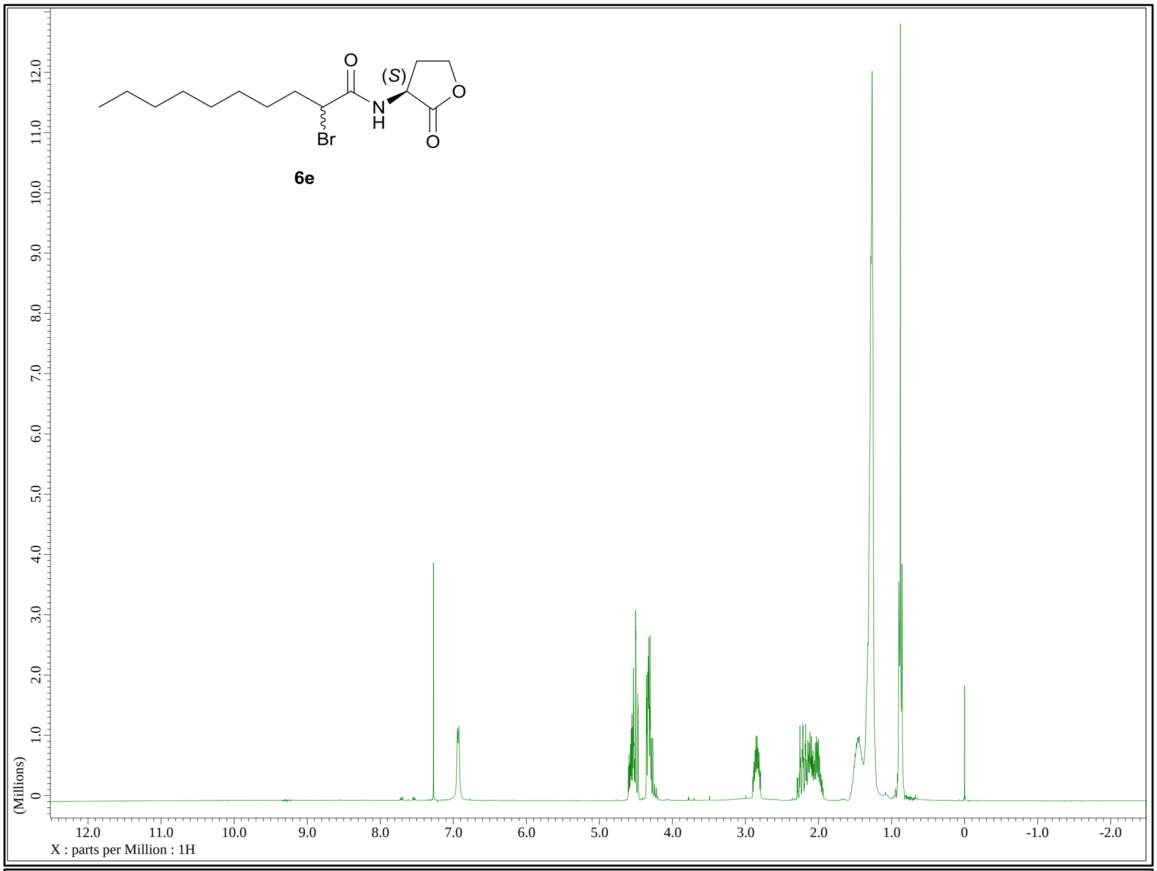

(S)
N
H
O
O
O
Br
6e
(Millions)
X : parts per Million : 1H

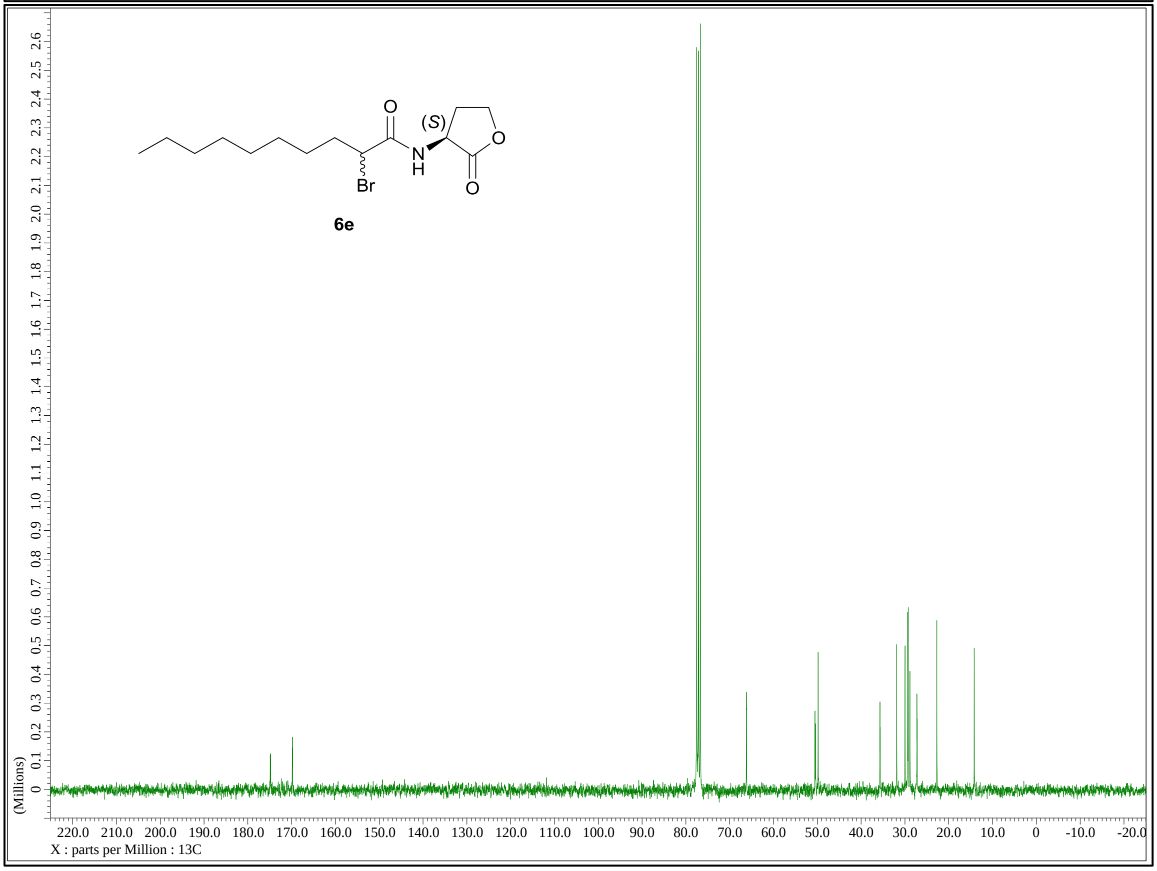

(S)
N
H
O
O
O
Br
6e
(Millions)
X : parts per Million : 13C

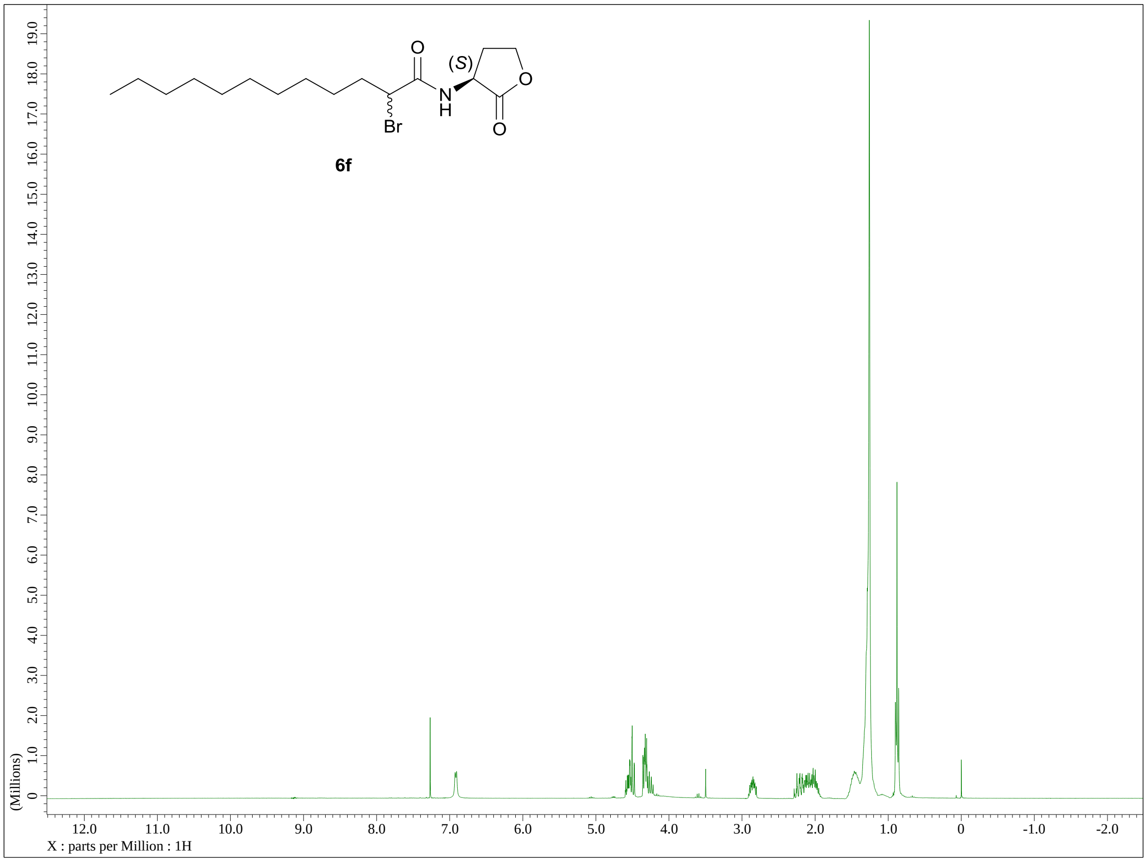
O
(S)
N
H
O
O
Br
6f
19.0 18.0 17.0 16.0 15.0 14.0 13.0 12.0 11.0 10.0 9.0 8.0 7.0 6.0 5.0 4.0 3.0 2.0 1.0 0
(Millions)
12.0 11.0 10.0 9.0 8.0 7.0 6.0 5.0 4.0 3.0 2.0 1.0 0 -1.0 -2.0
X : parts per Million : 1H

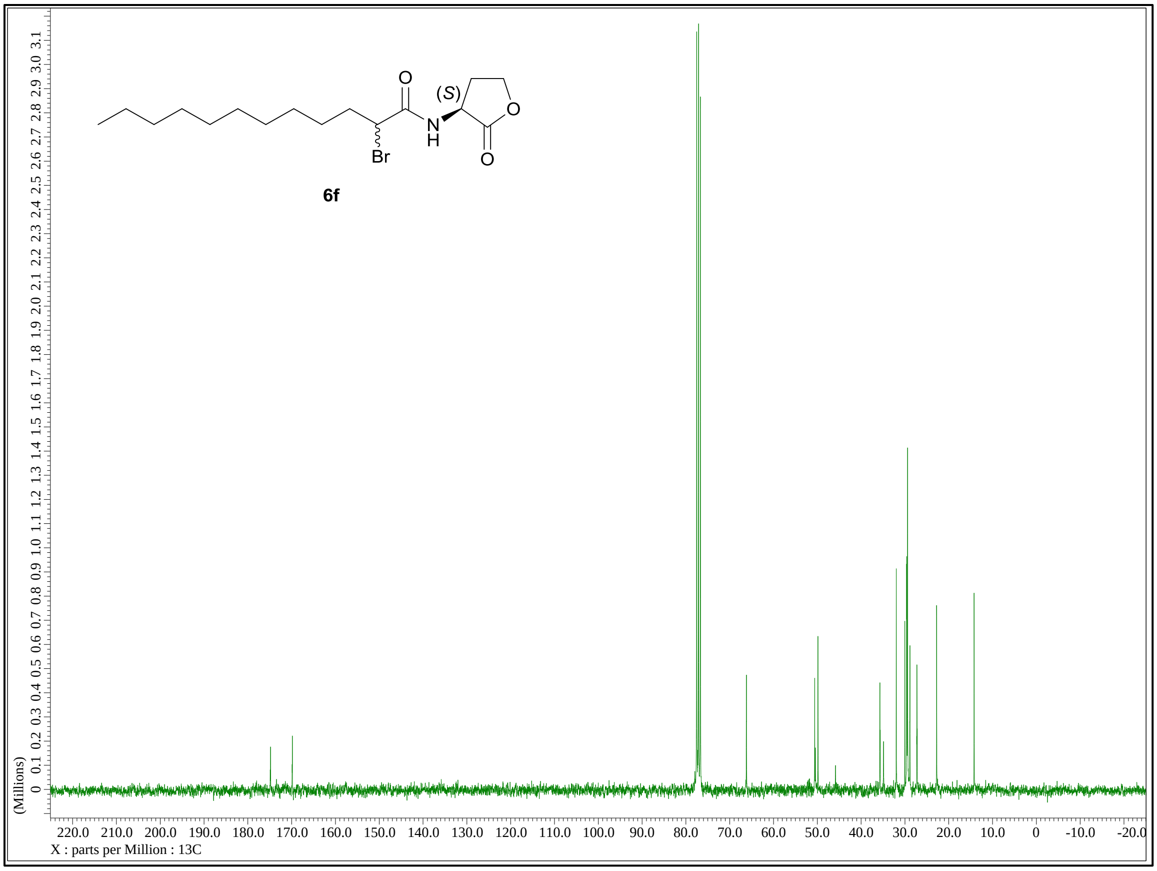
O
(S)
N
H
O
O
Br
6f
3.1 3.0 2.9 2.8 2.7 2.6 2.5 2.4 2.3 2.2 2.1 2.0 1.9 1.8 1.7 1.6 1.5 1.4 1.3 1.2 1.1 1.0 0.9 0.8 0.7 0.6 0.5 0.4 0.3 0.2 0.1 0
(Millions)
220.0 210.0 200.0 190.0 180.0 170.0 160.0 150.0 140.0 130.0 120.0 110.0 100.0 90.0 80.0 70.0 60.0 50.0 40.0 30.0 20.0 10.0 0 -10.0 -20.0
X : parts per Million : 13C

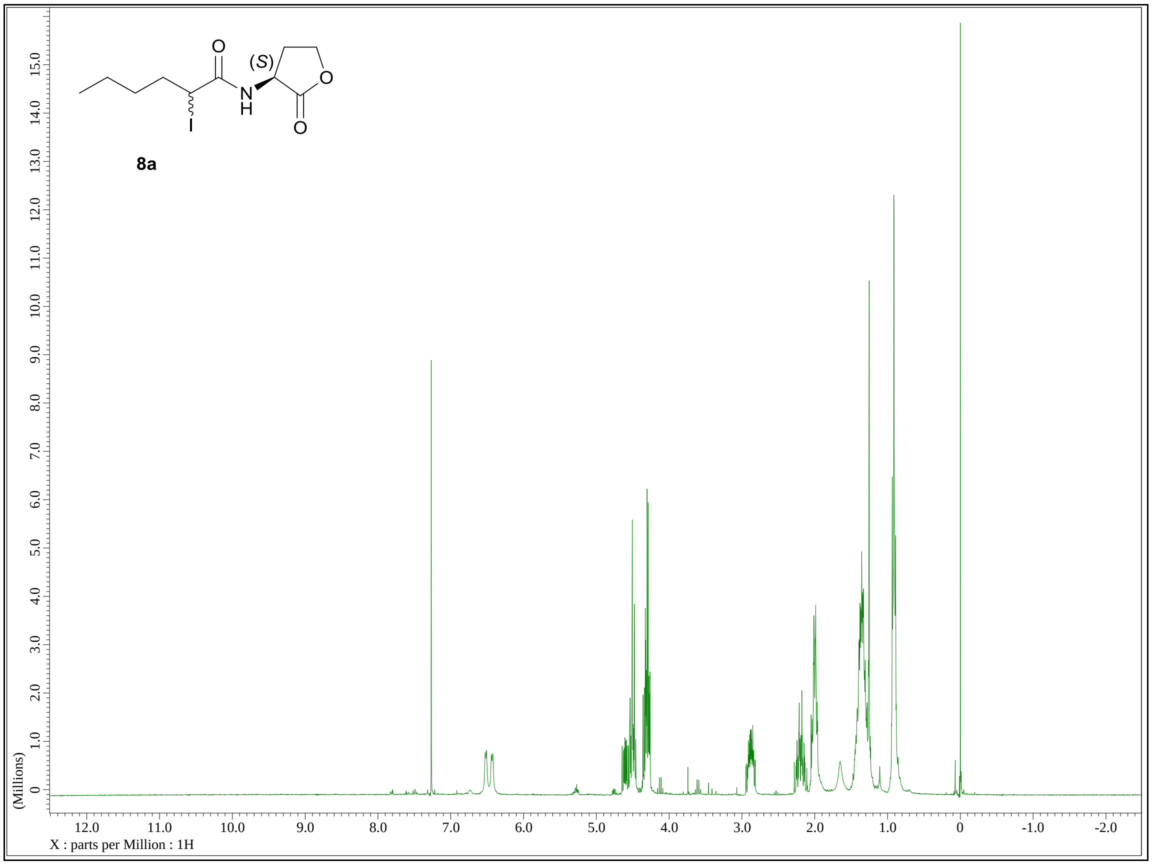
(S)
8a
(Millions)
X : parts per Million : 1H

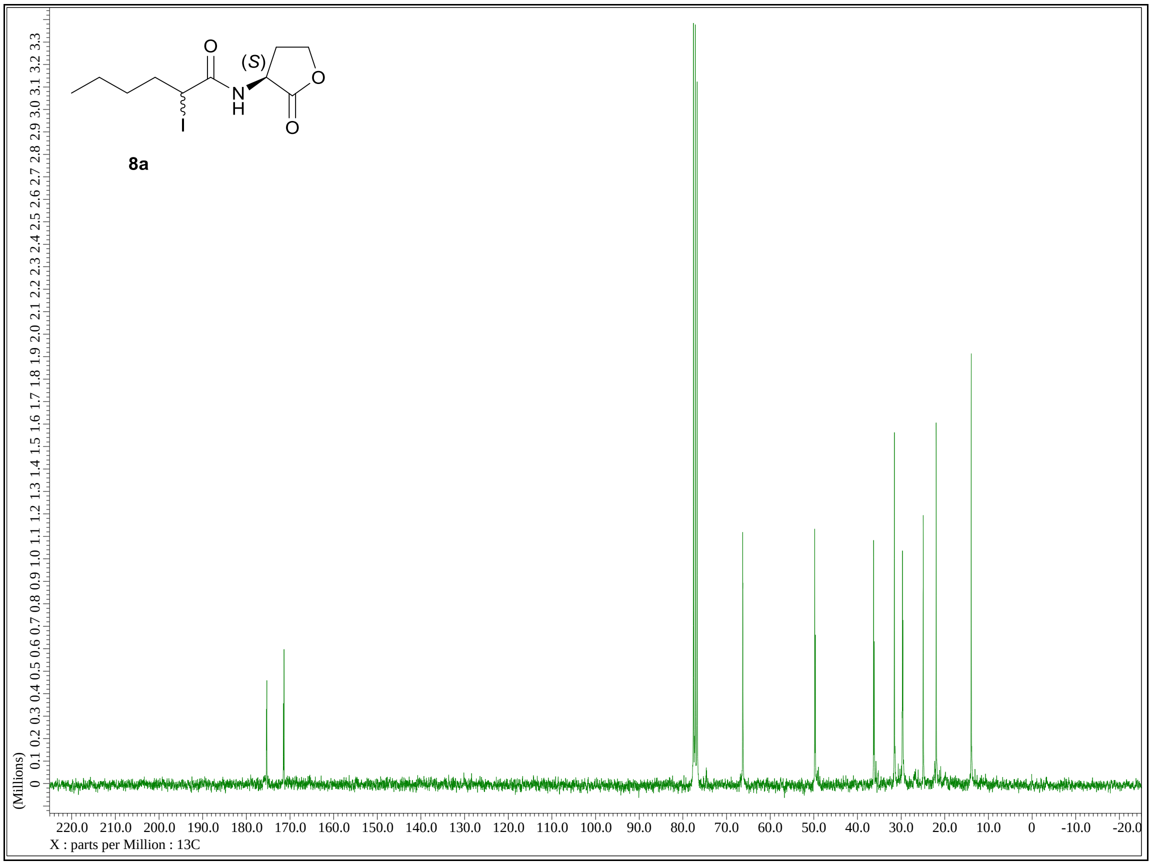
(S)
8a
(Millions)
X : parts per Million : 13C

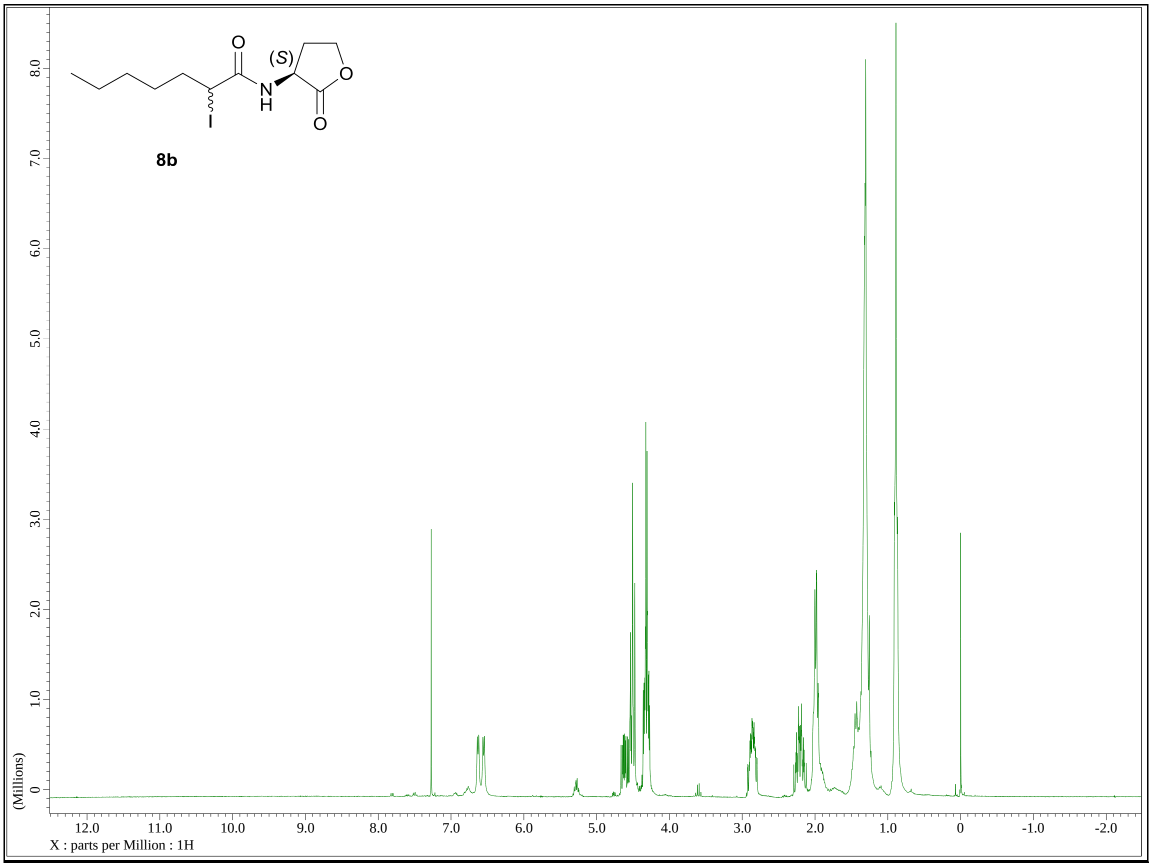

O
(S)
O
N
H
I
O
8b
(Millions)
X : parts per Million : 1H

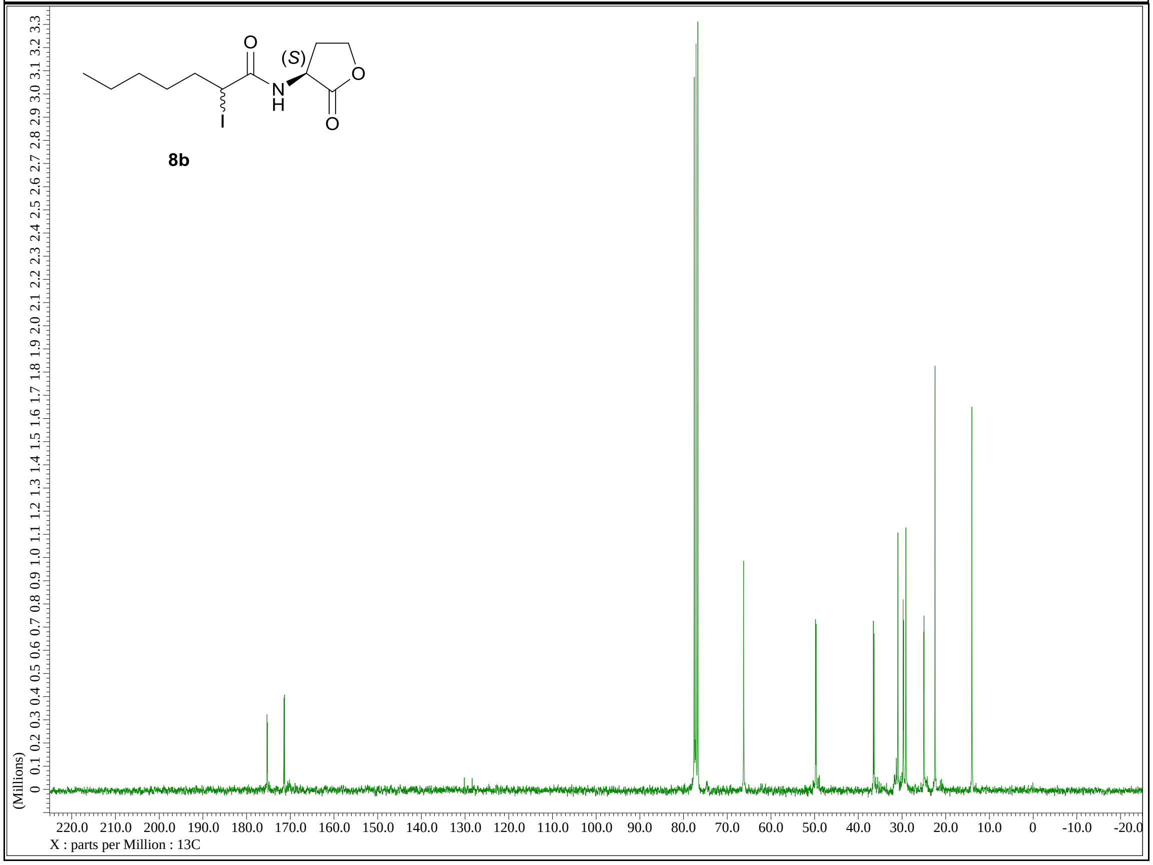

O
(S)
O
N
H
I
O
8b
(Millions)
X : parts per Million : 13C

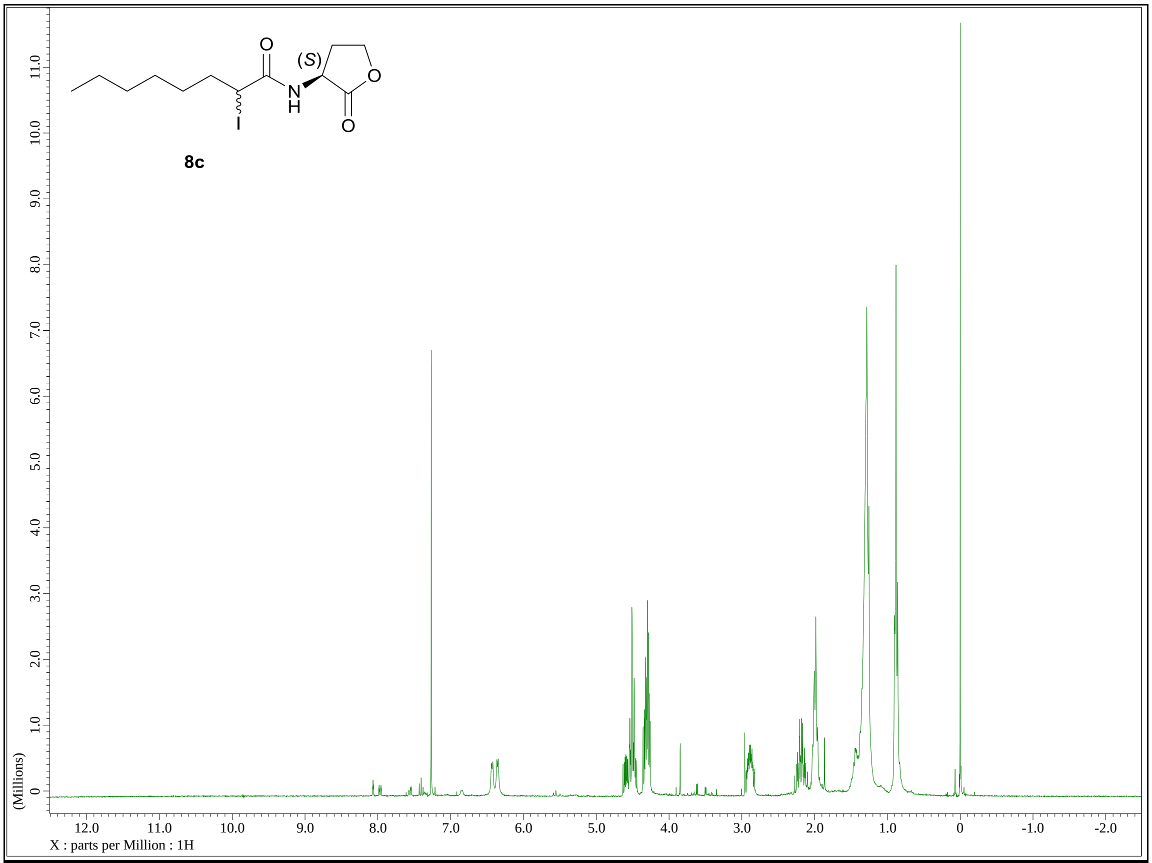
8c
(Millions)
X : parts per Million : 1H

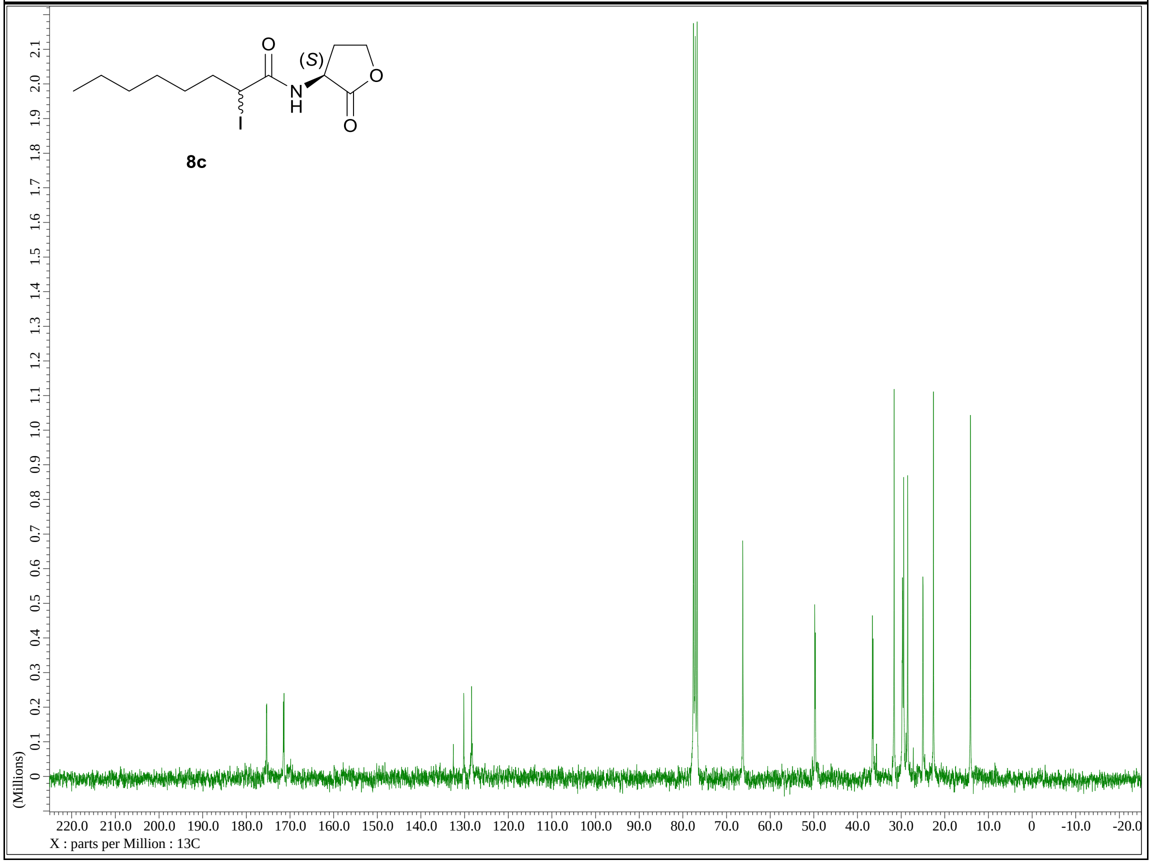
8c
(Millions)
X : parts per Million : 13C

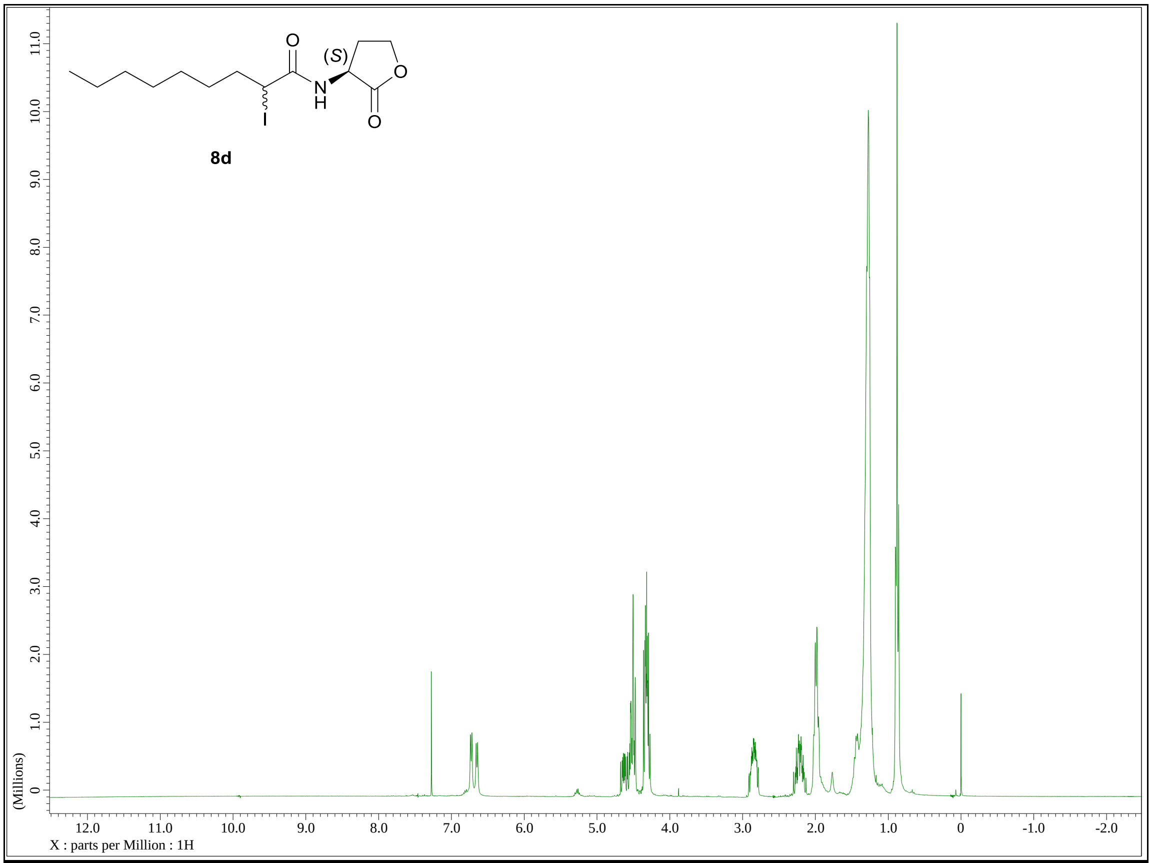
8d
(S)
(Millions)
X : parts per Million : 1H

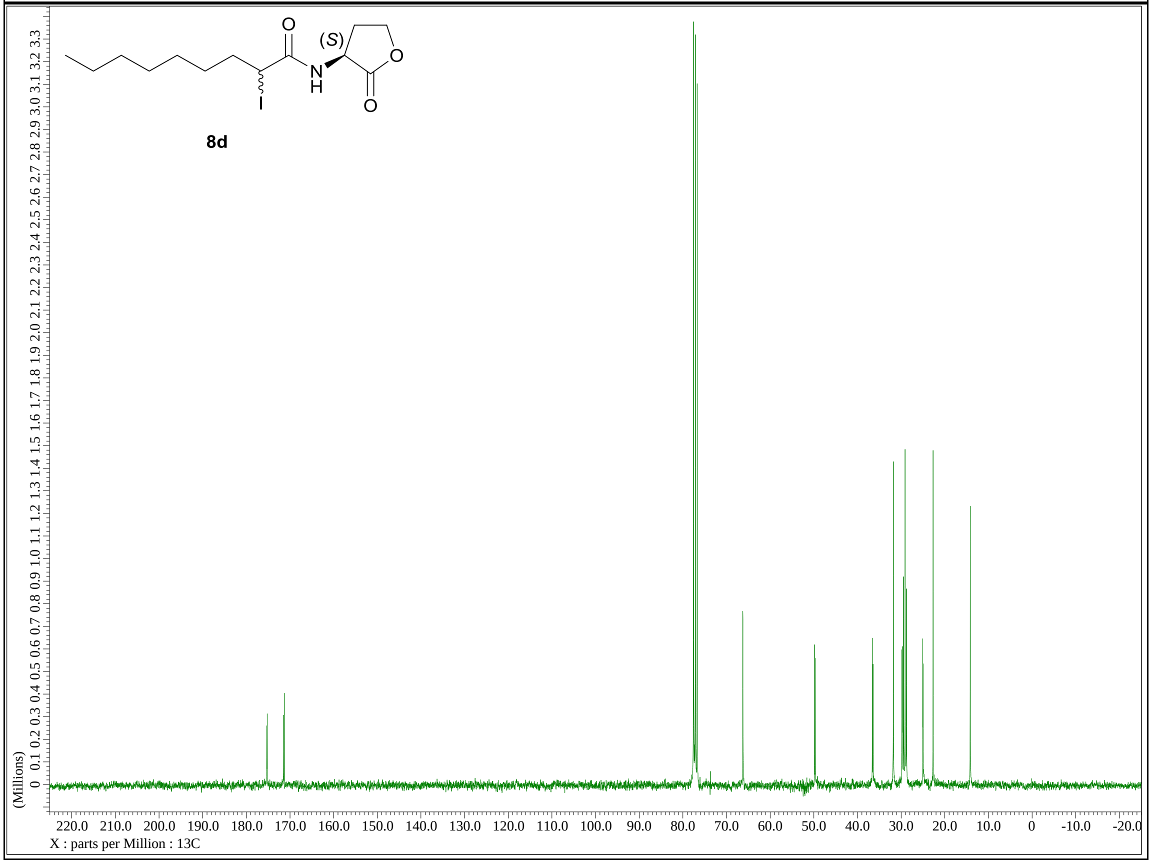
8d
(S)
(Millions)
X : parts per Million : 13C

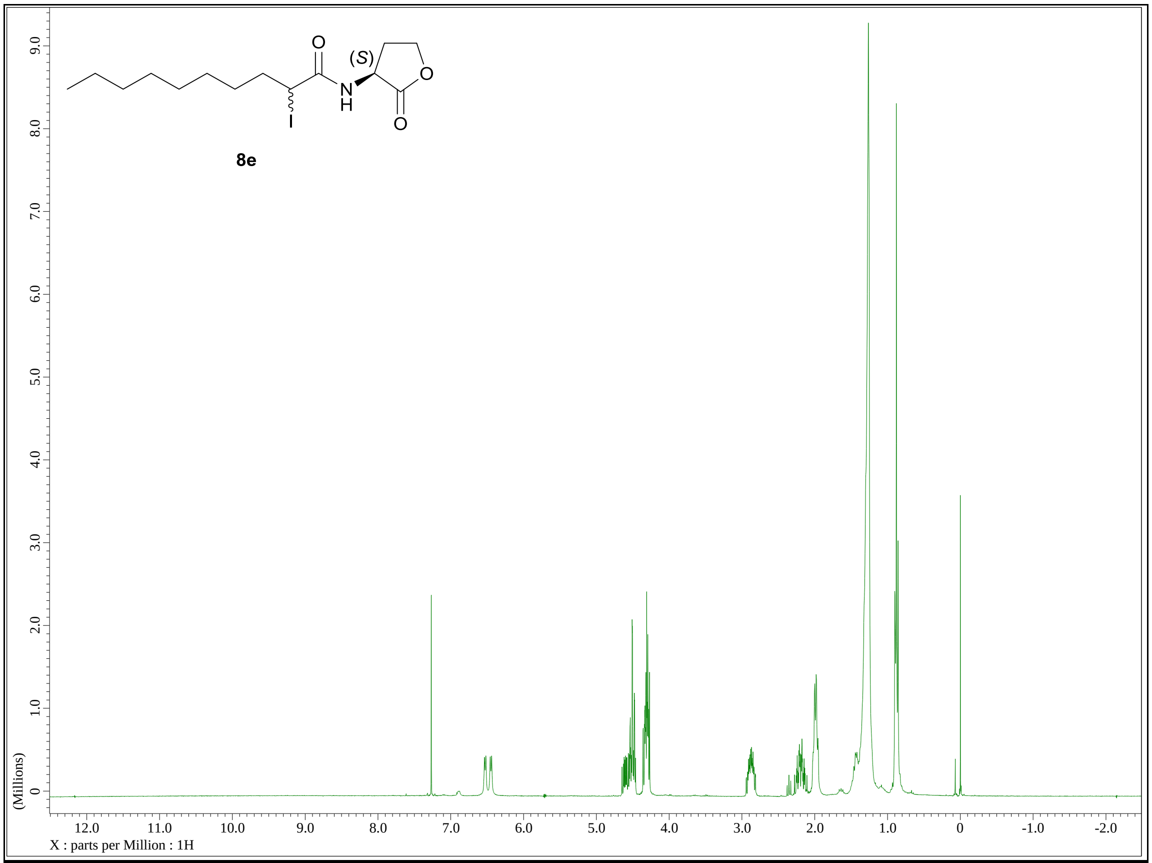

(S)
O
N
H
I
O
O
8e
(Millions)
9.0
8.0
7.0
6.0
5.0
4.0
3.0
2.0
1.0
0
12.0 11.0 10.0 9.0 8.0 7.0 6.0 5.0 4.0 3.0 2.0 1.0 0 -1.0 -2.0
X : parts per Million : 1H

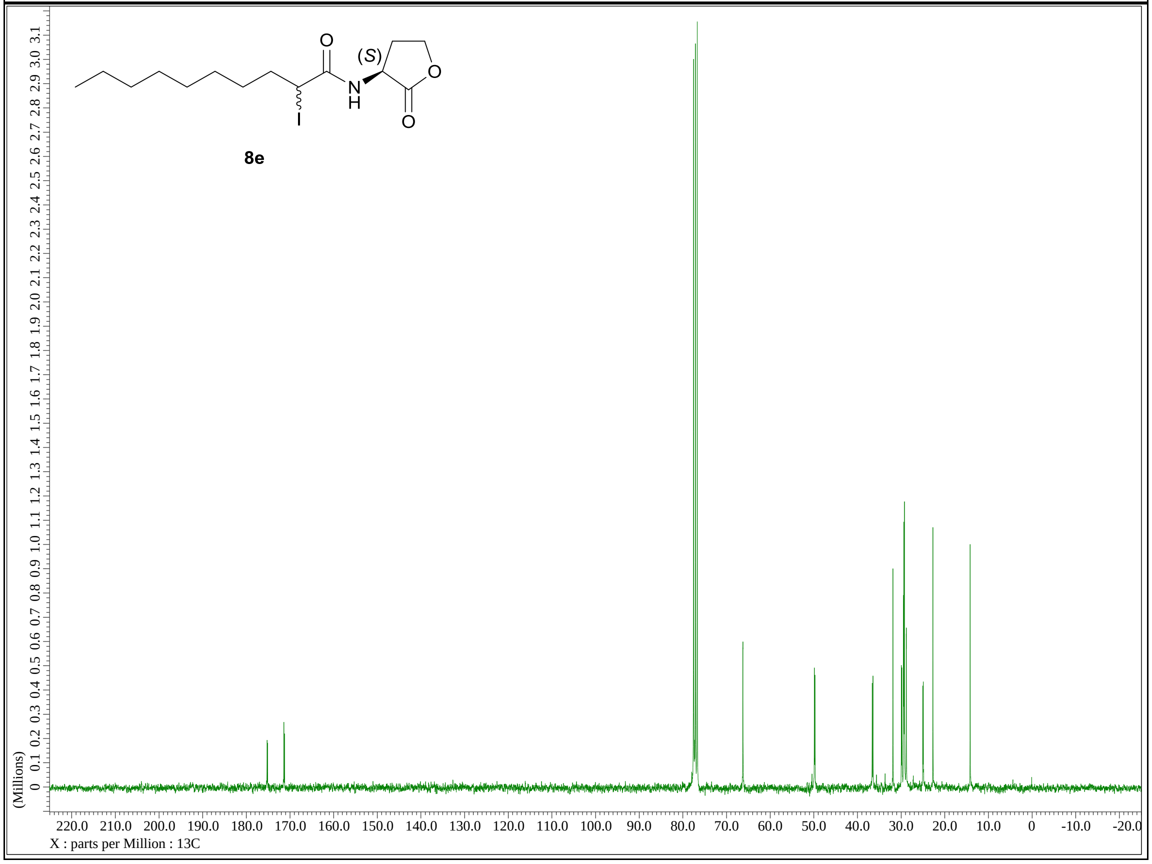

(S)
O
N
H
I
O
O
8e
(Millions)
0 0.1 0.2 0.3 0.4 0.5 0.6 0.7 0.8 0.9 1.0 1.1 1.2 1.3 1.4 1.5 1.6 1.7 1.8 1.9 2.0 2.1 2.2 2.3 2.4 2.5 2.6 2.7 2.8 2.9 3.0 3.1
220.0 210.0 200.0 190.0 180.0 170.0 160.0 150.0 140.0 130.0 120.0 110.0 100.0 90.0 80.0 70.0 60.0 50.0 40.0 30.0 20.0 10.0 0 -10.0 -20.0
X : parts per Million : 13C

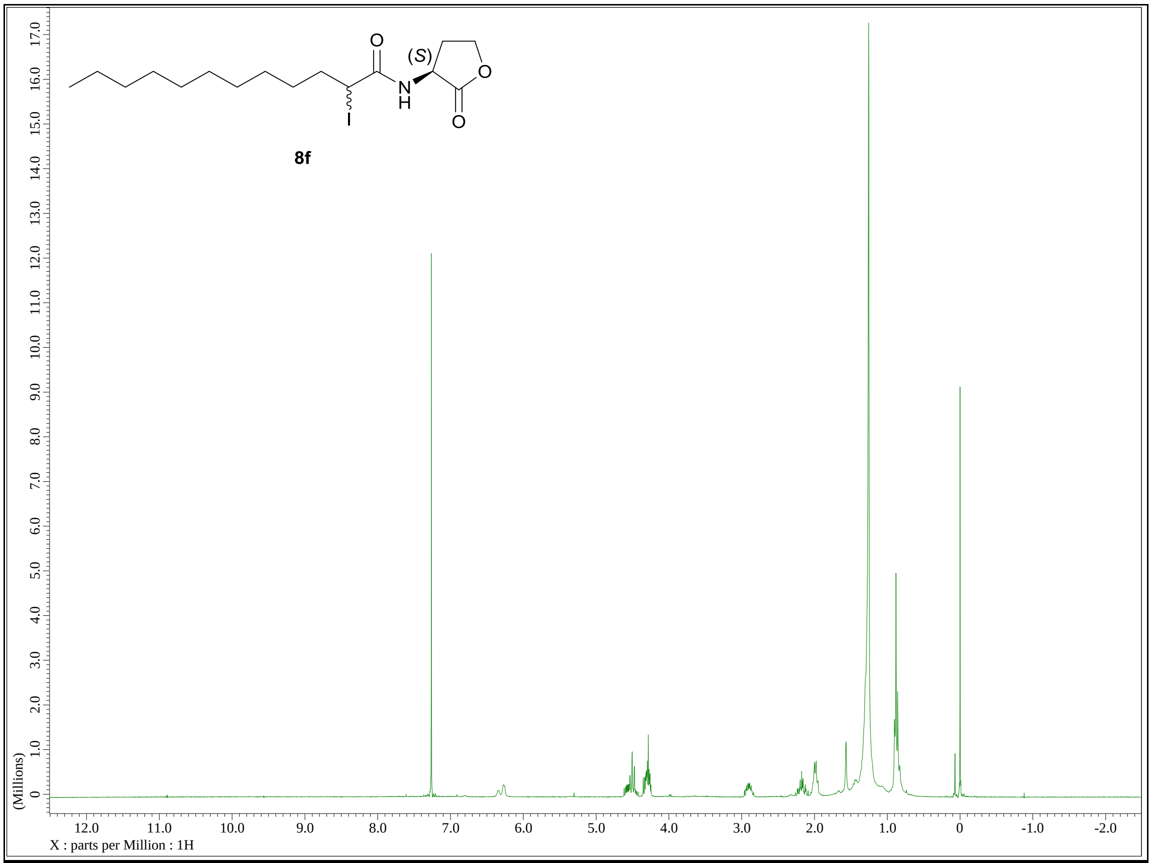
8f
(Millions)
X : parts per Million : 1H

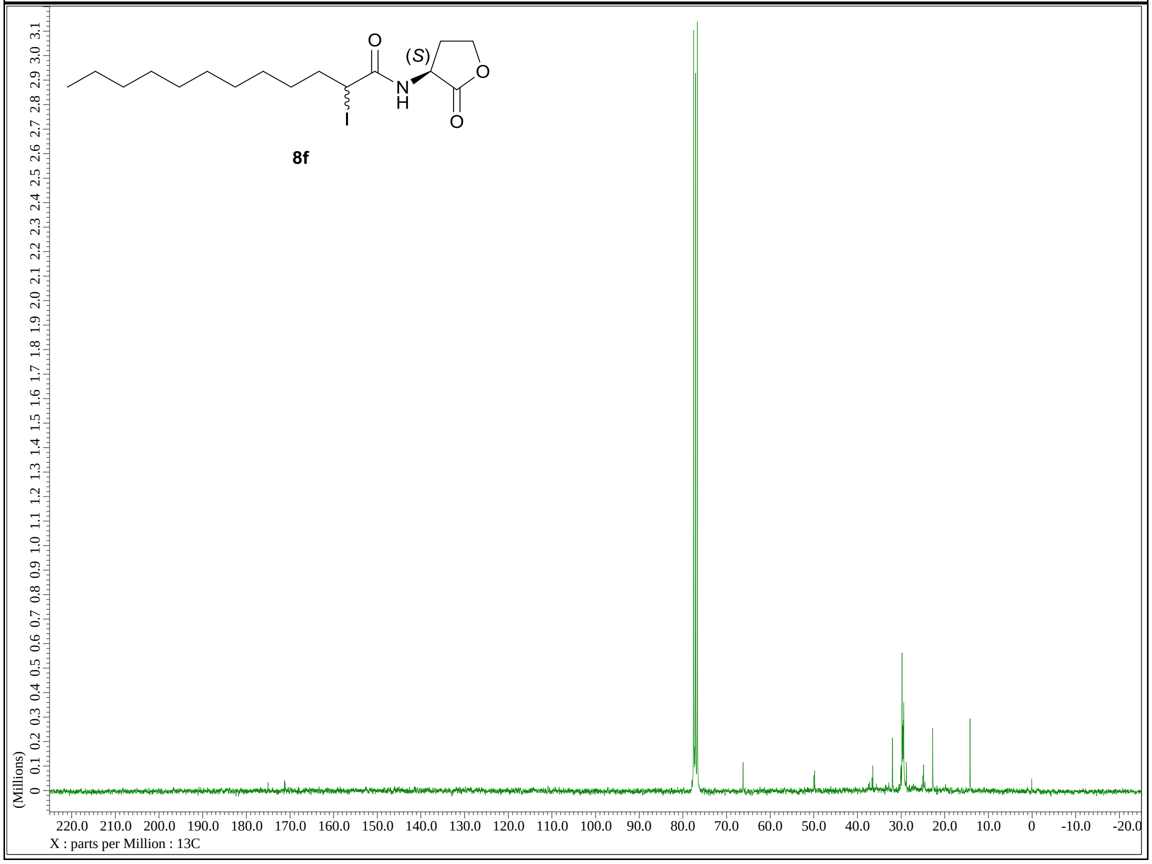
8f
(Millions)
X : parts per Million : 13C

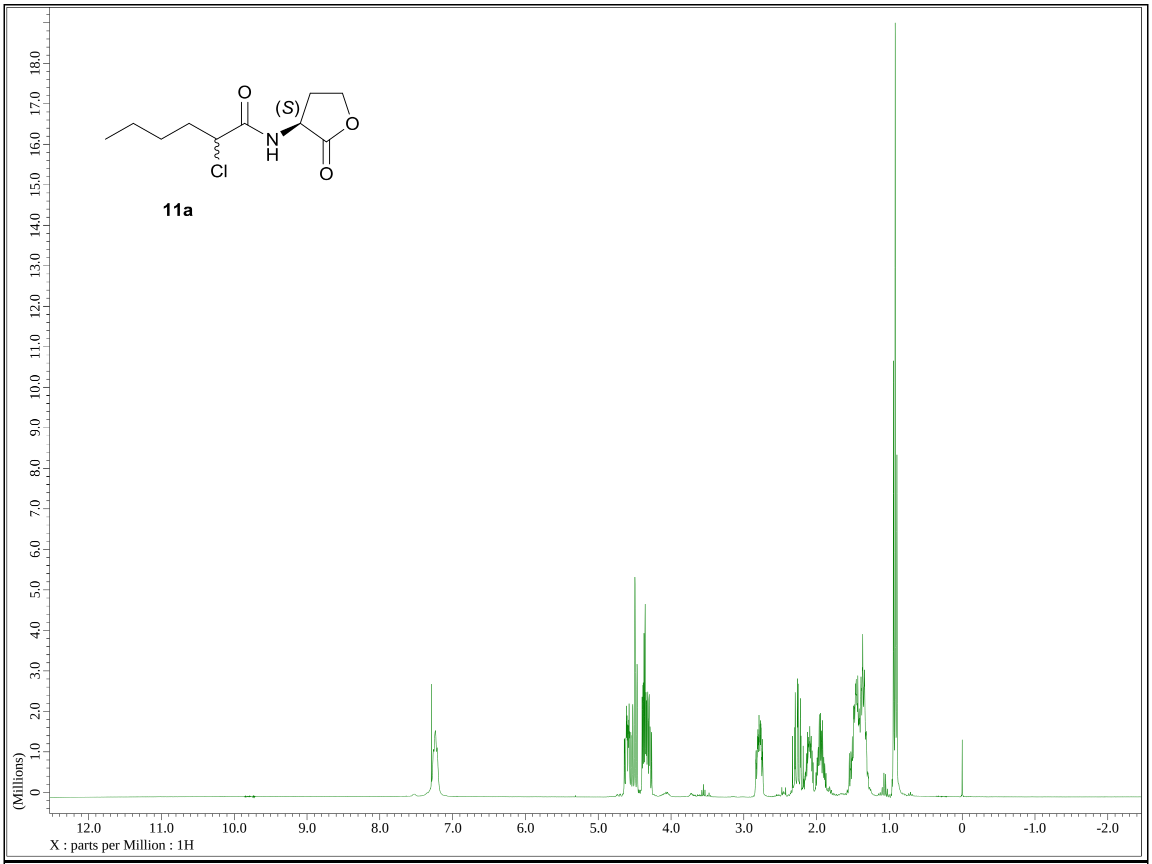
(S)
11a
(Millions)
X : parts per Million : 1H

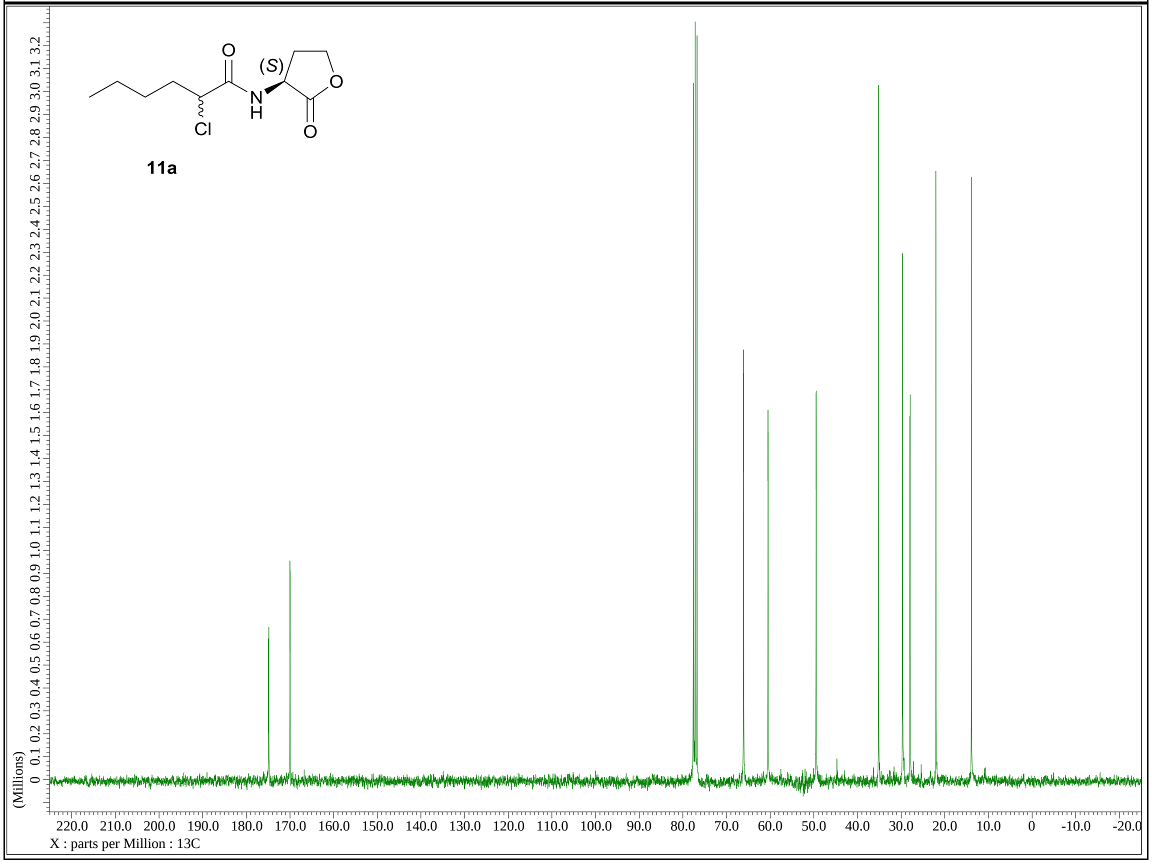
(S)
11a
(Millions)
X : parts per Million : 13C

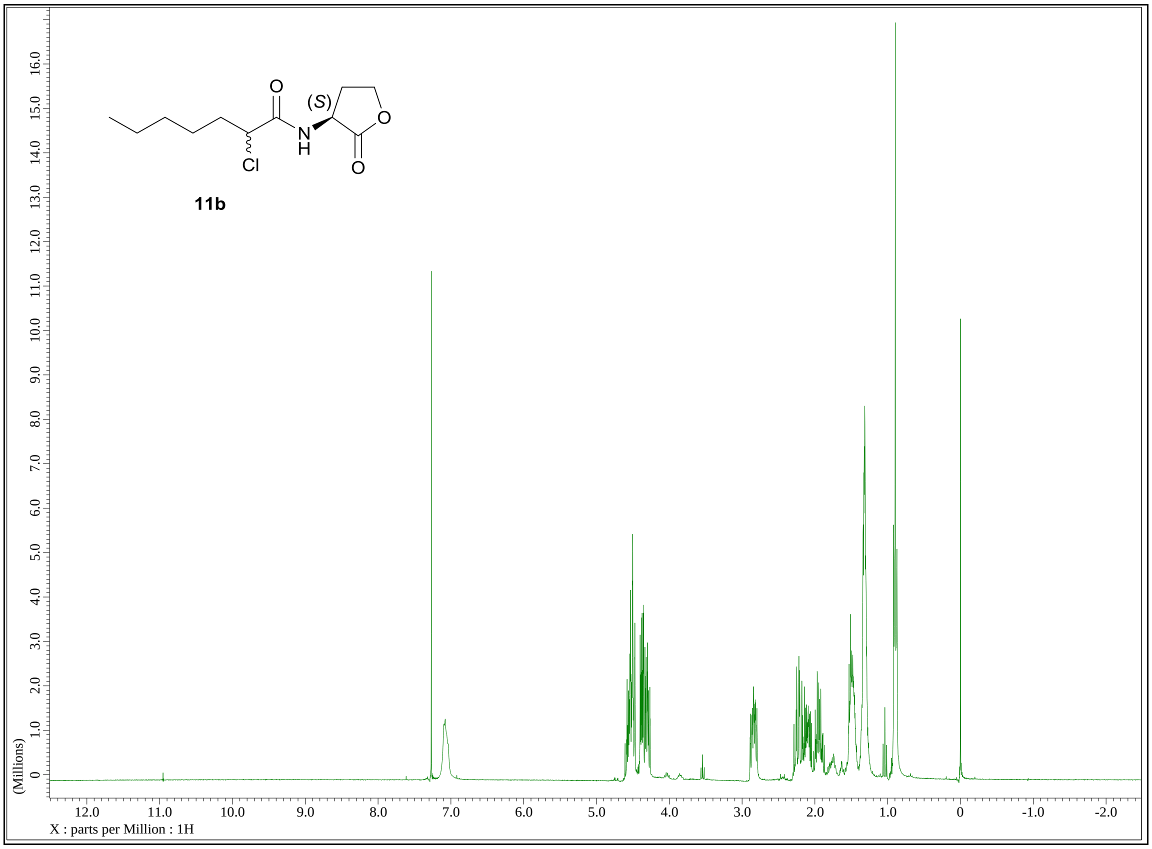

(S)
11b
(Millions)
X : parts per Million : 1H

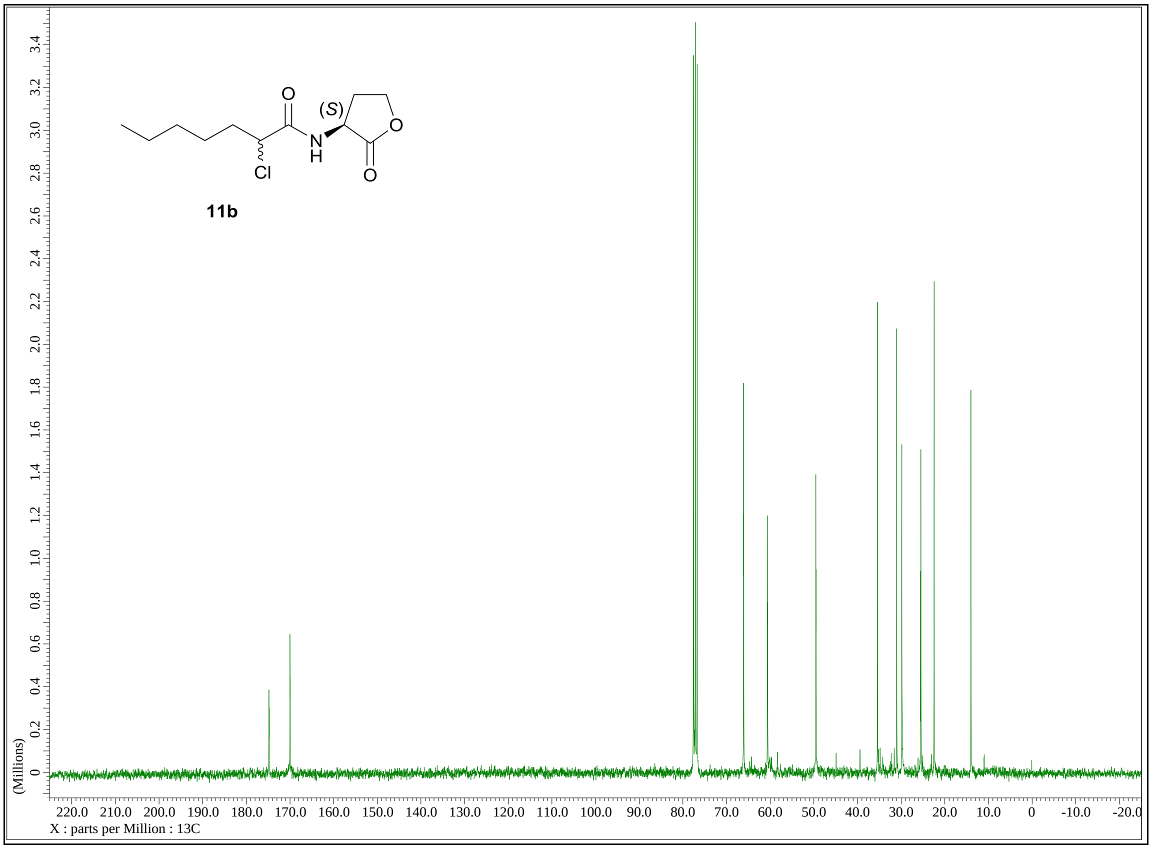

(S)
11b
(Millions)
X : parts per Million : 13C

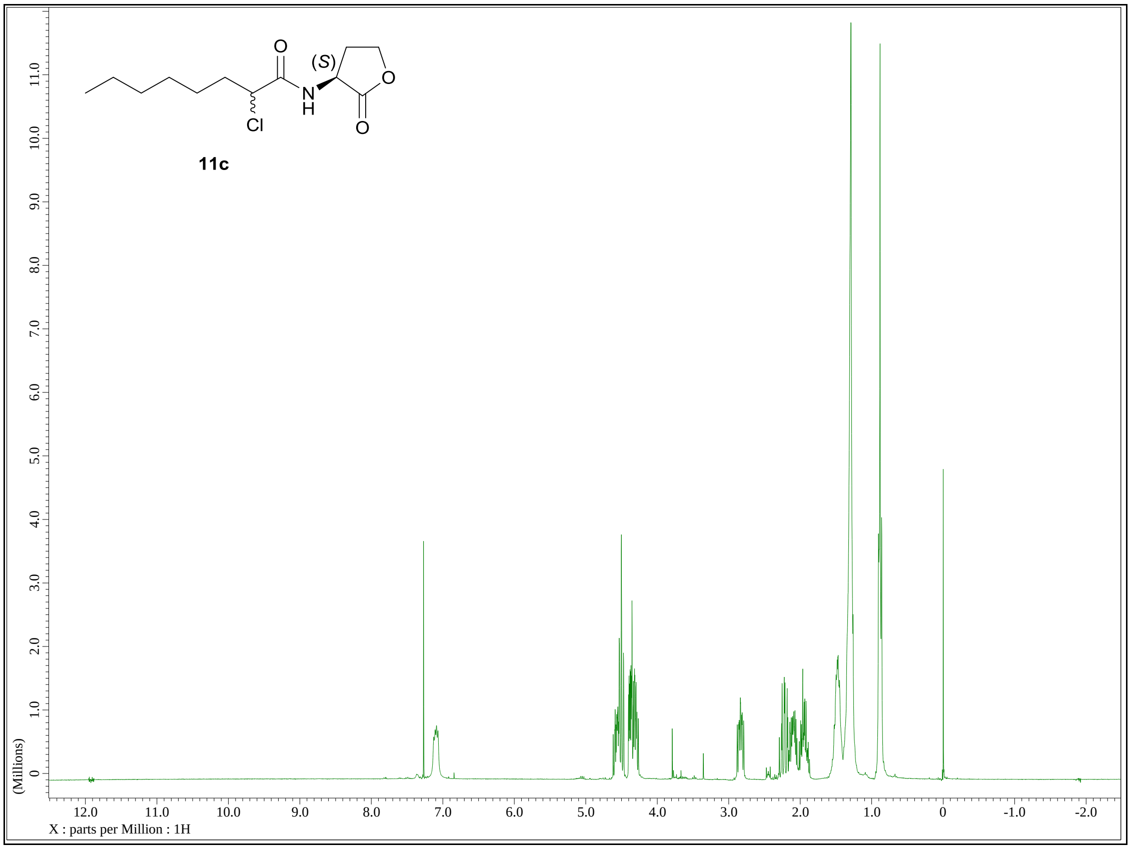
O
(S)
O
N
H
Cl
O
11c
(Millions)
X : parts per Million : 1H

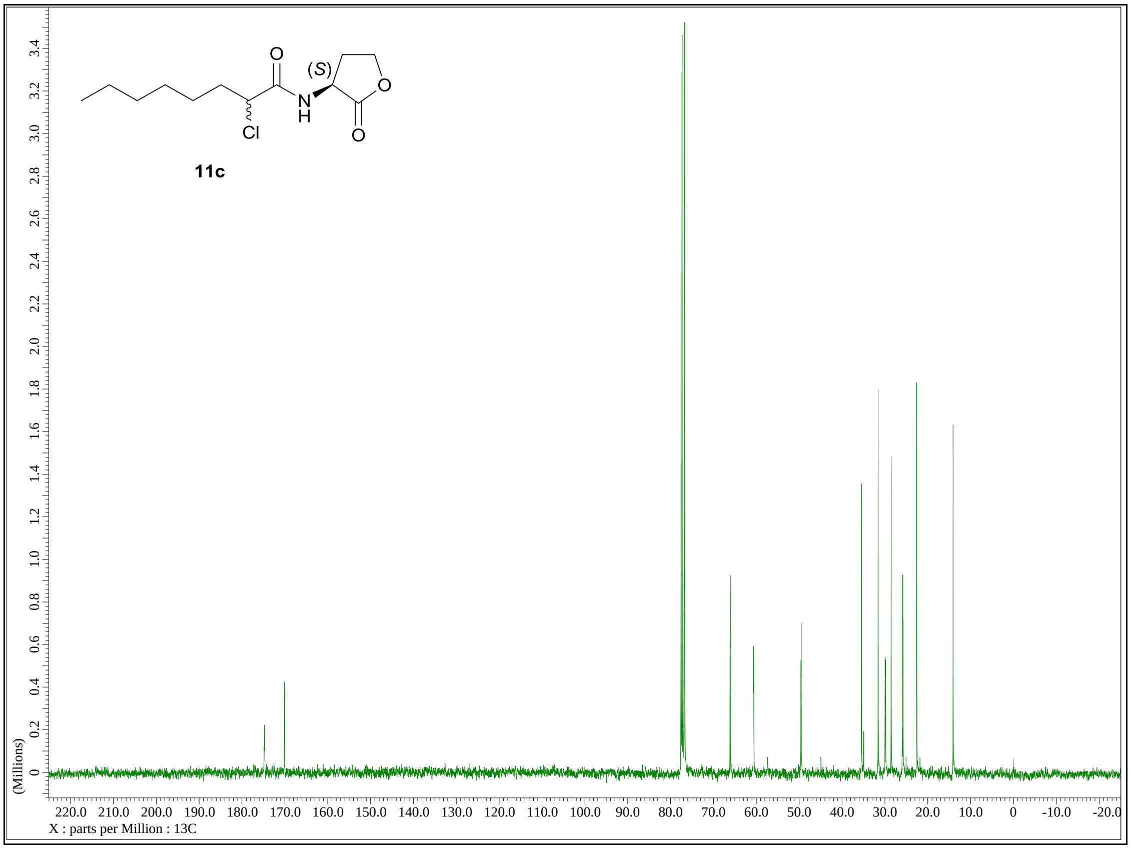
O
(S)
O
N
H
Cl
O
11c
(Millions)
X : parts per Million : 13C

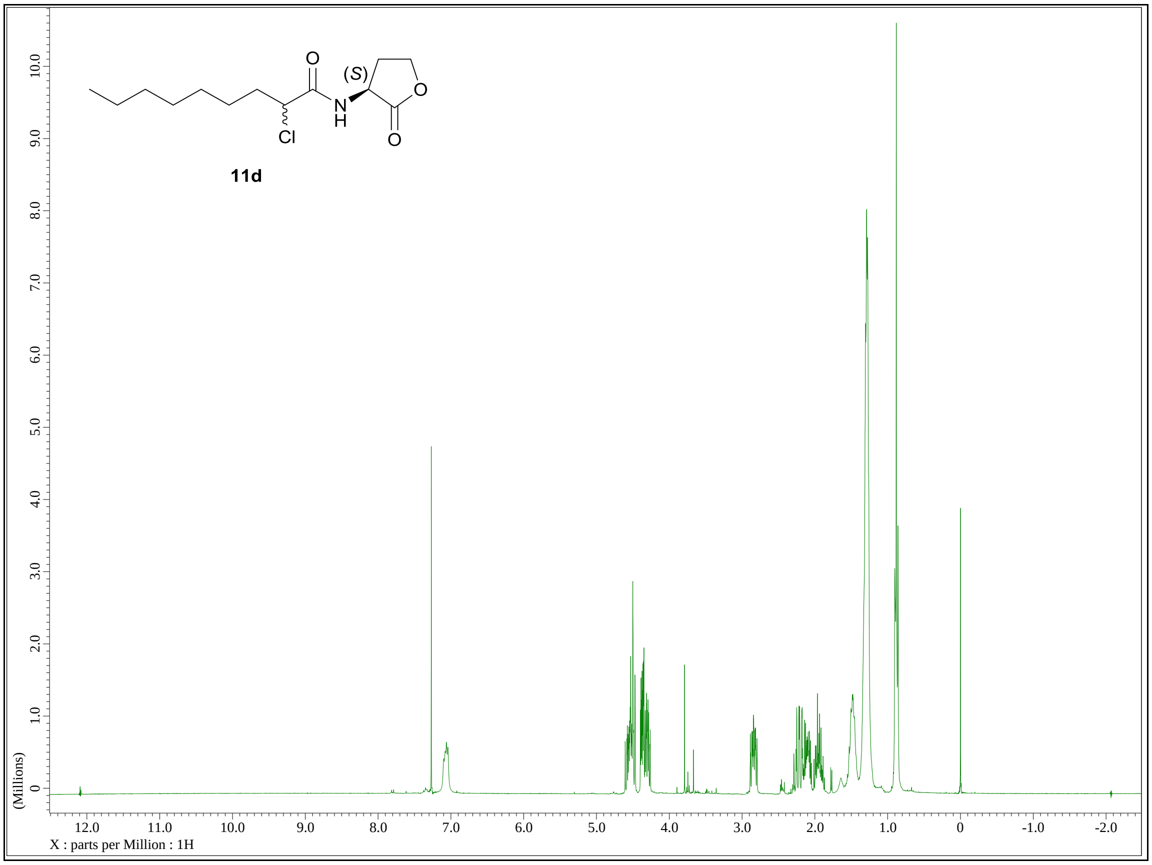
O
(S)
O
N
H
Cl
O
11d
(Millions)
X : parts per Million : 1H

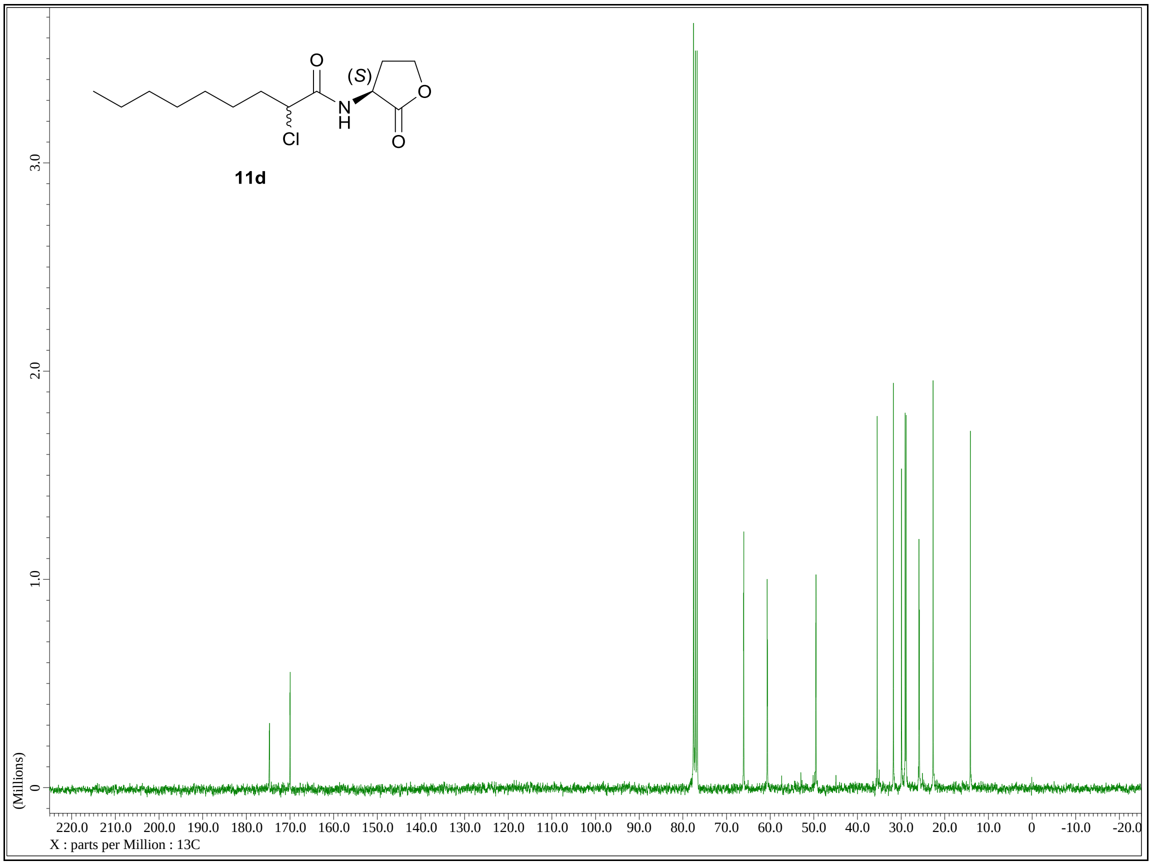
O
(S)
O
N
H
Cl
O
11d
(Millions)
X : parts per Million : 13C

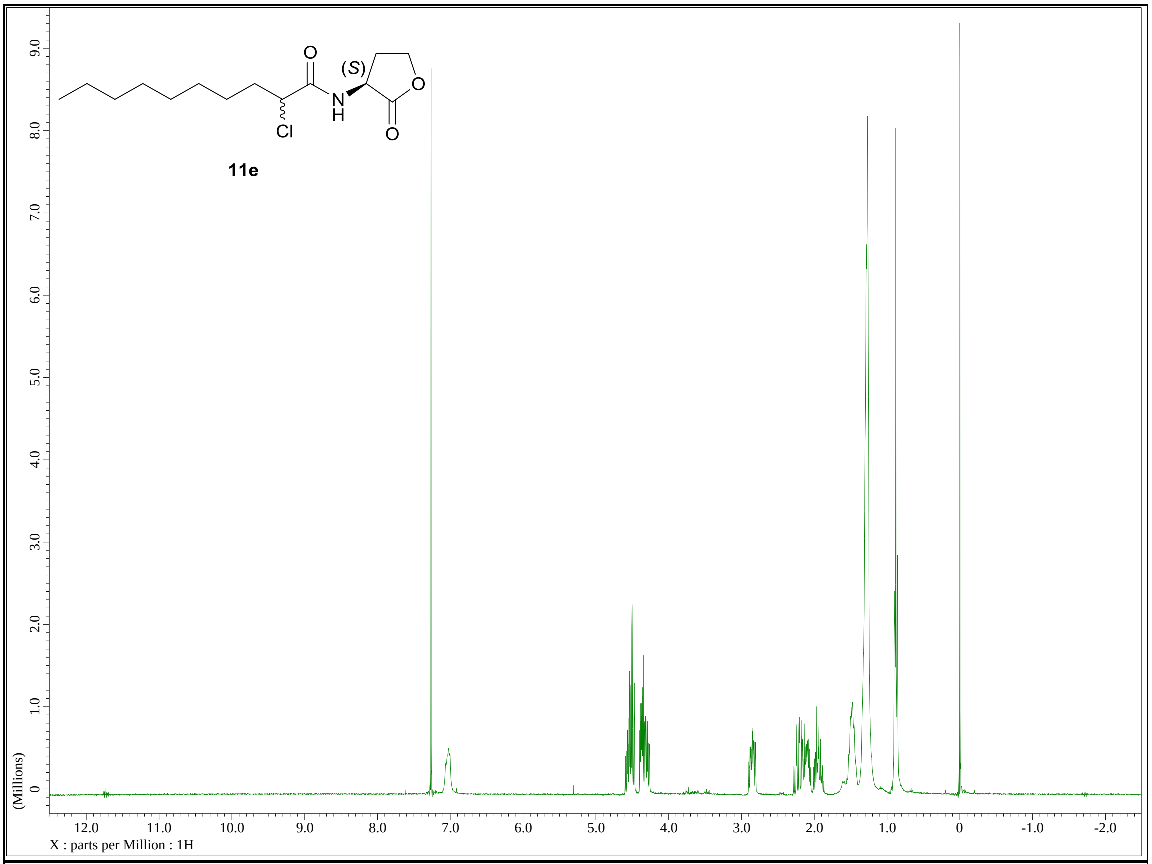
O
(S)
O
N
H
Cl
O
11e
(Millions)
X : parts per Million : 1H

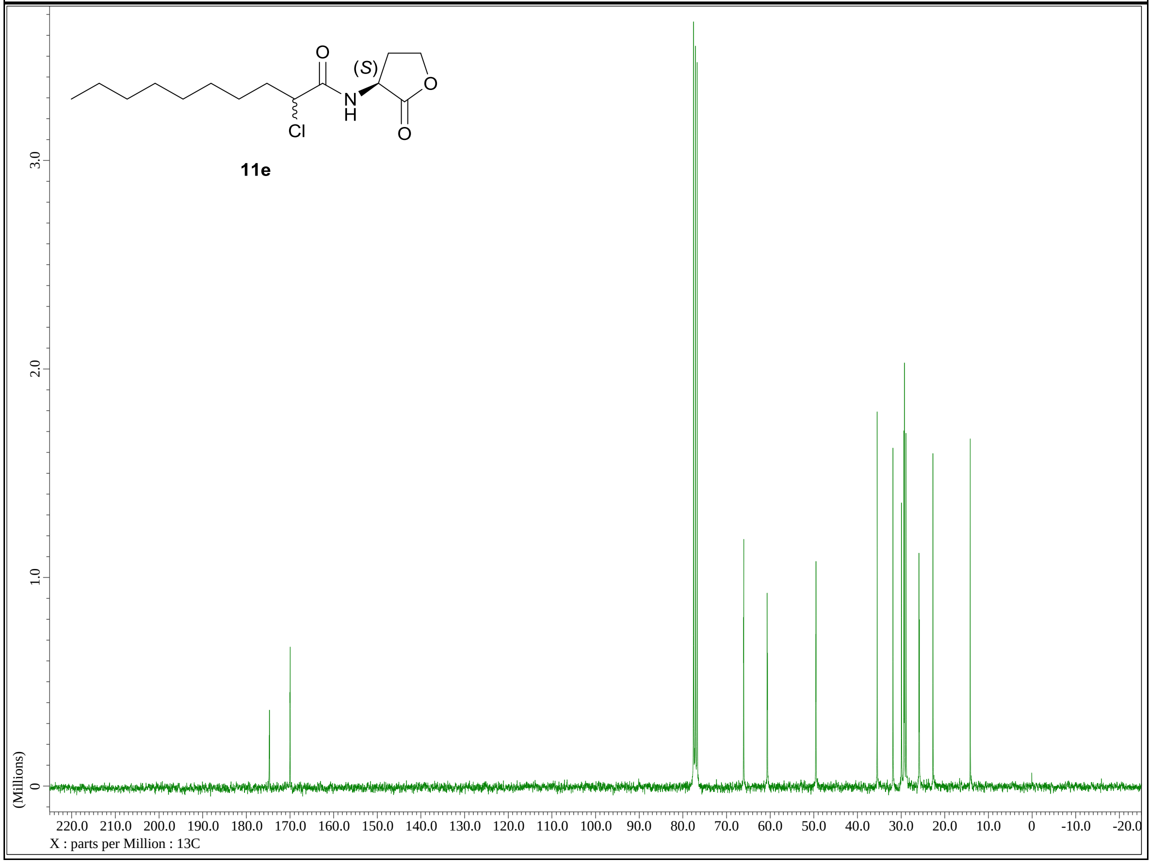
O
(S)
O
N
H
Cl
O
11e
(Millions)
X : parts per Million : 13C

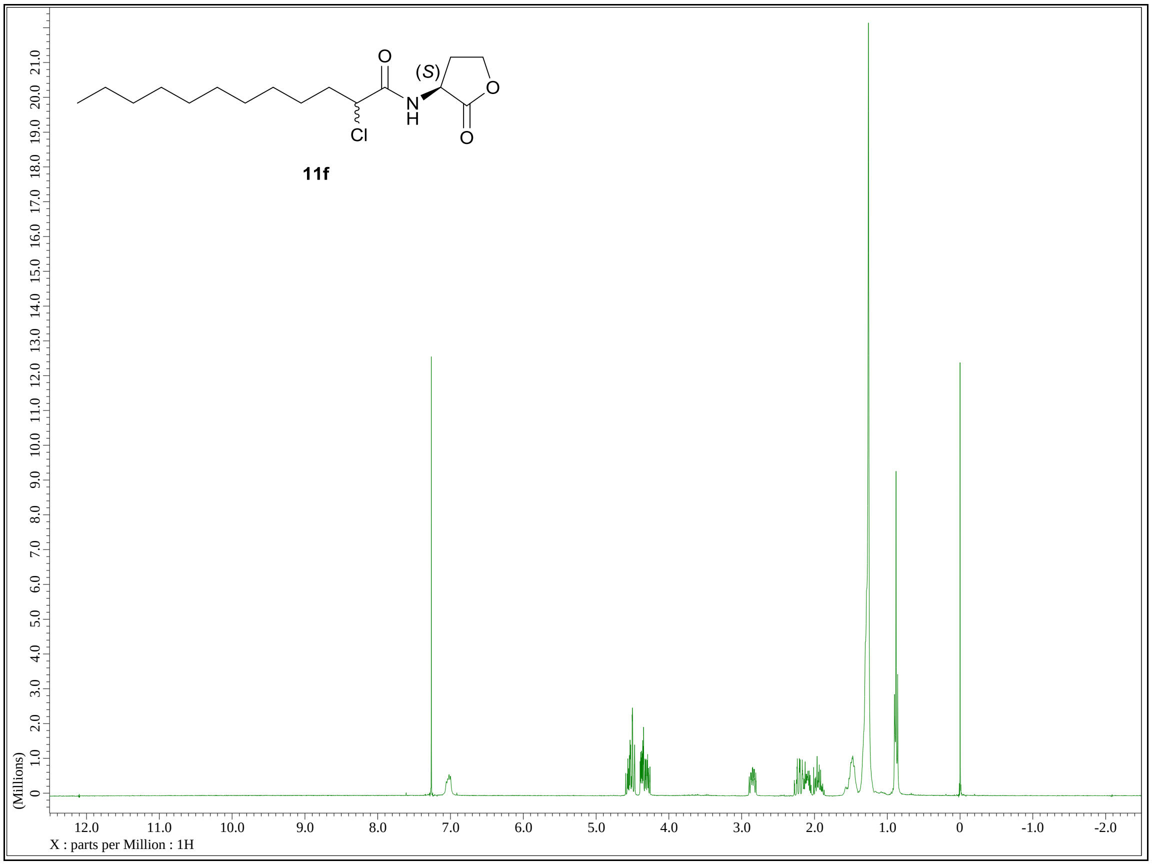

(S)
O
N
H
Cl
O
O
11f
(Millions)
X : parts per Million : 1H

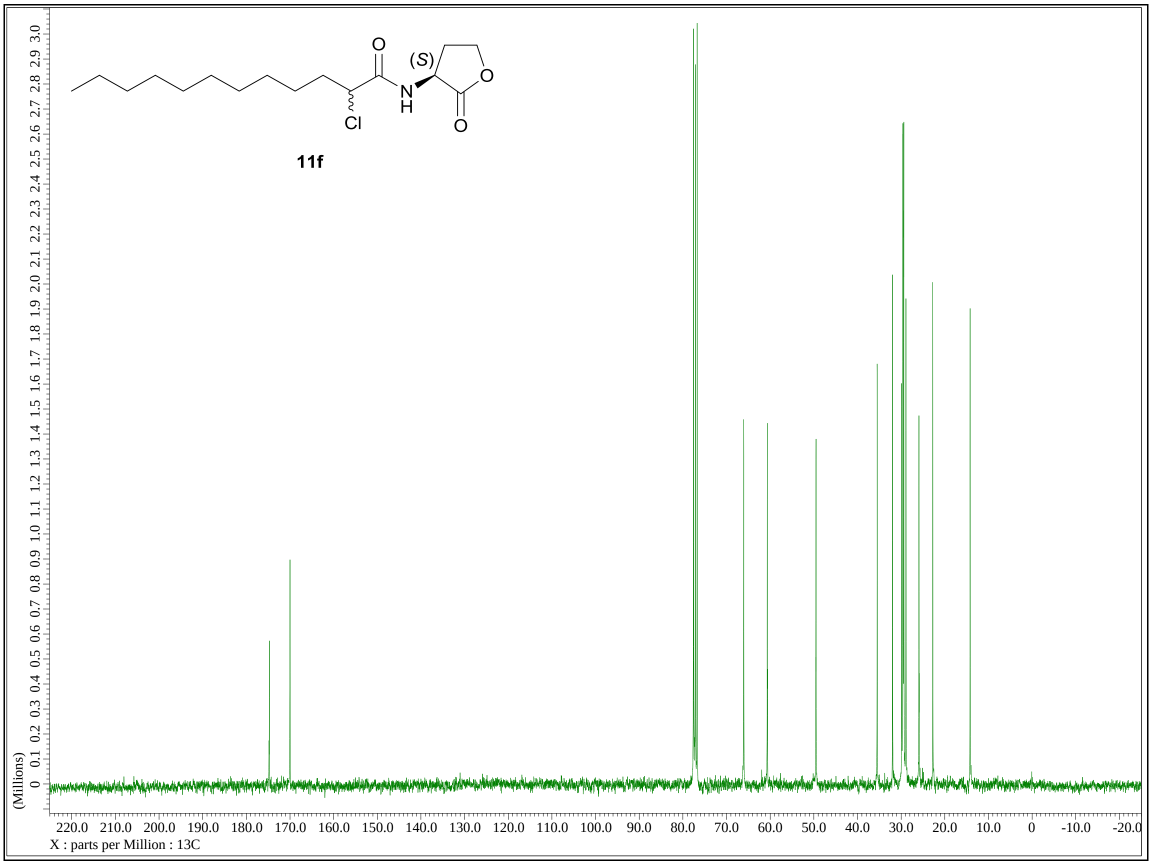

(S)
O
N
H
Cl
O
O
11f
(Millions)
X : parts per Million : 13C
